# Polyoxoplatinates as covalently dynamic electron sponges and molecular electronics materials†

**DOI:** 10.1039/d1na00387a

**Published:** 2021-08-13

**Authors:** Aleksandar Kondinski, Mahdi Ghorbani-Asl

**Affiliations:** Department of Chemical Engineering and Biotechnology, University of Cambridge Philippa Fawcett Dr Cambridge CB3 0AS UK aleksandar@kondinski.com; Institute of Ion Beam Physics and Materials Research Helmholtz-Zentrum Dresden-Rossendorf 01328 Dresden Germany mahdi.ghorbani@hzdr.de

## Abstract

In organic systems, dynamic covalent chemistry provides an adaptive approach (*i.e.*, “covalent dynamics”) where thermodynamic equilibria are used to tailor structural and electronic changes in molecular assemblies. The covalent dynamics finds utility in the design of novel self-healing materials, sensors, and actuators. Herein, using density functional theory (DFT) we explore the structural, electronic and transport properties of the Pt-based polyoxometalate (POM) [Pt^III^_12_O_8_(SO_4_)_12_]^4−^ and its derivatives. The latter POM has six redox responsive {O–Pt–Pt–O} moieties and prospects for storage of up to twelve electrons, thus exemplifying how dynamic covalent chemistry may manifest itself in fully inorganic systems. Simulations of the Au/POM/Au junction show that the electron conduction strongly depends on the redox of the POM but more weakly on its rotations with respect to the Au surface. Moreover, the POM shows promising spin-polarized current behaviour, which can be modulated using bias and gate voltages.

## Abstract 2

DFT studies corroborate that Pt-based polyoxometalates exhibit impressive redox stability and prospects for applications in molecular electronics.

## Graphical abstract

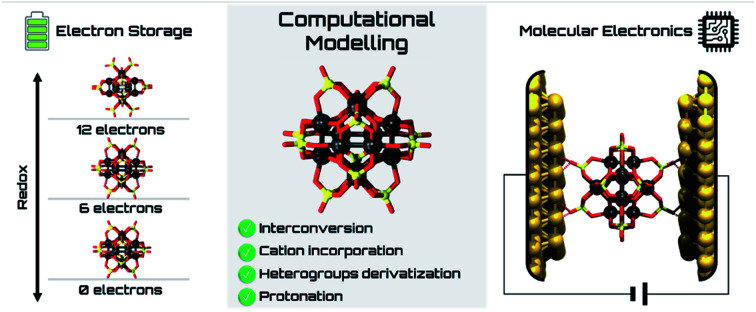

## Introduction

The development of metal oxide nanoclusters has remained an attractive scientific and technological goal for over a century.^1^ Among the different classes of metal oxide nanoclusters, polyoxometalates (POMs) are a well-developed class representative of early transition metals in high oxidation states (V, Nb, Ta, Mo, and W).^2,3^ Many plenary (*i.e.* “saturated”) POMs are often used as catalysts that can operate at broad thermal, pH and electrochemical ranges.^4,5^ Furthermore, POMs exhibit tailored electronic structures and magnetic properties that make them technologically relevant in an umbrella of emerging “POMtronics”^6^ that include applications in photovoltaics,^7,8^ memory devices,^9–12^, electron storage^13–15^ and single POM transistors.^16^

**Fig. 1:**
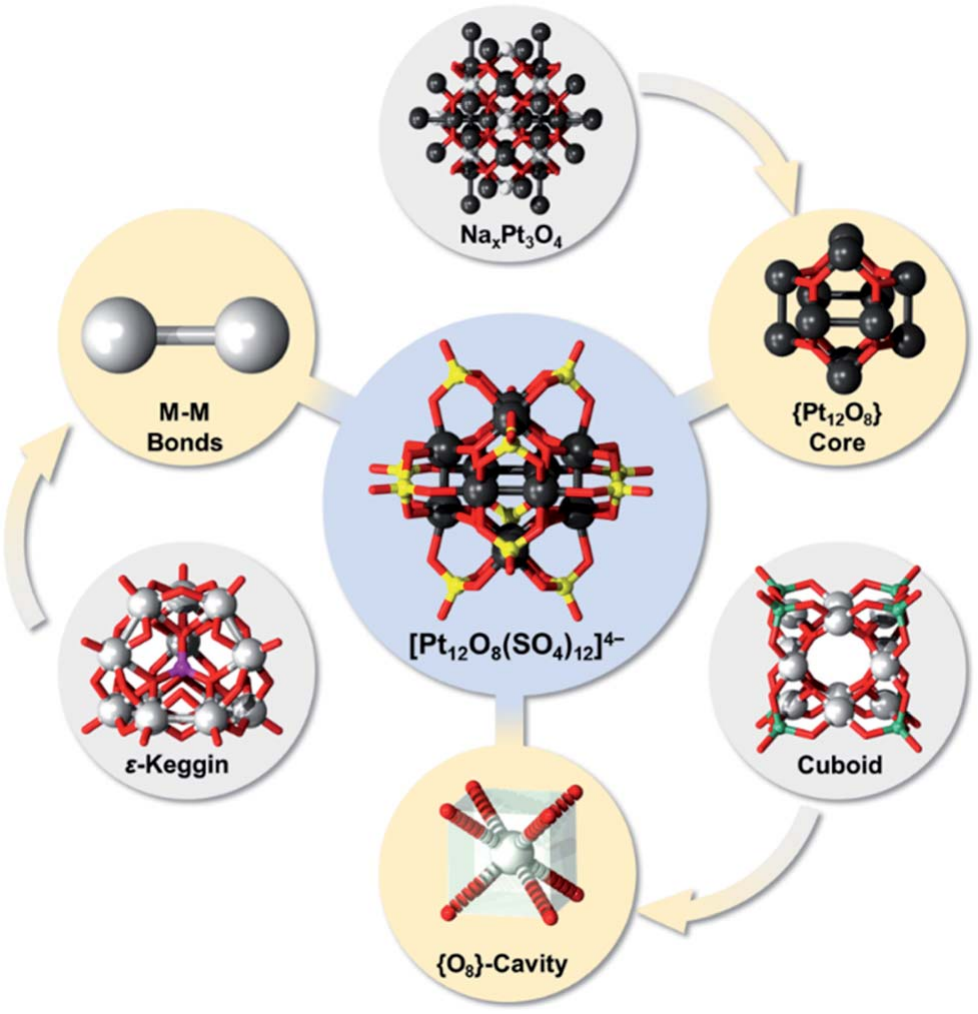
Schematic representation of the [Pt_12_O_8_(SO_4_)_12_]^4−^ structure in the large blue circle centre and the three related structural archetypes in grey circles: waserite Na_*x*_Pt_3_O_4_, ε-Keggin {PMo_12_O_40_} and cuboidal {Pd_12_O_8_(XO_4_)_8_}. The three different features shared with [Pt_12_O_8_(SO_4_)_12_]^4−^ are highlighted in yellow circles. Colour code: Pt = black, O = red, Pd/Mo = grey, P = violet, X = green, Na = white.

Over the past two decades, it has been widely recognized that late transition metals can build POMs. In contrast to the early transition metal-based POMs that exhibit d^0^ (oxidized) or d^1–2^ (reduced) electronic occupancy, late transition metal-based POMs adopt the d^6–9^ configuration of the addendum centres, enabling diverse coordination geometries and structural complexity. Prominent examples of the latter class are polyoxocuprates,^17,18^ polyoxoaurates^15,19,20^ and polyoxopalladates.^21–27^ Polyoxoplatinates or Pt-based POMs are also known, but historically their structural discovery has been challenging owing to the slow reaction kinetics of Pt(ii) in water.^28^ Examples of polyoxoplatinates include Privalov's [(NO_2_)_3_Pt^IV^Pt^II^_3_O_3_(NO_2_)_6_]^5−^ and [Pt^IV^(Pt^II^_3_O_3_(NO_2_)_3_)_2_]^8−^,^29,30^ and Korenev's [PtPt^IV^_4_(μ_3_-OH)_2_(μ_2_-OH)_4_(NO_3_)_10_] and [Pt^IV^_6_(μ_3_-OH)_4_(μ_2_-OH)_6_(NO_3_)_12_]^2+^.^31^ However, Wickleder's [Pt^III^_12_O_8_(SO_4_)_12_]^4−^ discovered in 2004 remains the most prominent polyoxoplatinate known to date (Fig. 1).^32,33^ Although in the original work Wickleder referred to [Pt^III^_12_O_8_(SO_4_)_12_]^4−^ as a cluster ion,^32^ owing to the high oxo-ligand saturation [Pt^III^_12_O_8_(SO_4_)_12_]^4−^ is widely recognized as a POM,^3,34–37^ or more precisely as a form of metal–metal bonded POM.^38^ The polyanion [Pt_12_O_8_(SO_4_)_12_]^4−^ was originally isolated as deep red single crystals of the ammonium salt formed by reacting Pt(NO_3_)_2_ and concentrated sulfuric acid in a sealed glass ampoule at 350 °C for three days followed by slow cooling.^32^ The latter conditions are highly oxidising and suitable for formation of POMs or extended metal oxides,^39,40^ but are rather uncommon for the formation of metal or alloy type cluster materials with carbonyl or thiol protecting ligands.^41–44^

The polyanion [Pt^III^_12_O_8_(SO_4_)_12_]^4−^ exhibits an ideal pyritohedral *T*_h_ point group symmetry reminiscent of a volleyball. Wickleder's [Pt^III^_12_O_8_(SO_4_)_12_]^4−^ can be virtually deconvoluted as a central {O_8_}-cube whose faces are capped by six {Pt–Pt} bonded moieties, forming a virtual {Pt_12_O_8_} structural motif. The {Pt_12_O_8_} structural motif is then encapsulated by twelve peripheral μ_3_-(SO_4_) groups. Each heterogroup has one free terminal oxo ligand and three oxo-bridging ligands. Two of the bridging oxo ligands bind to a single {Pt–Pt} moiety in an equatorial fashion while the one remaining oxo-ligand connects a single Pt^III^ centre from a neighbouring {Pt–Pt} moiety axially (see Fig. 1).

In recent years, there has been increasing interest in the synthesis and theoretical description of oxygen-deficient metal-oxo prisms with cluster-like character and cubic aromaticity.^45–47^ Classical POMs normally do not exhibit such cluster-like or metal–metal bonding character; however, only very recently was it unveiled that, although rare and not always well described, metal–metal bonding in POMs can be very useful towards the development of advanced POM functionalities (*e.g.* covalent dynamics) and their application in electron storage materials, sustainable technologies and “POMtronics”.^38^ Along these lines, [Pt^III^_12_O_8_(SO_4_)_12_]^4−^ is also an example of a metal–metal bonded POM. Structurally, [Pt^III^_12_O_8_(SO_4_)_12_]^4−^ is a “saturated” (*i.e.*, plenary) POM characterized by high thermal stability^32,33^ and high symmetry that endow this POM with a “Keplerate” architecture.^48^ The {Pt_12_O_8_} core of [Pt^III^_12_O_8_(SO_4_)_12_]^4−^ resembles the ordering of Pt and O centres in Adams' catalyst,^49^ which is the waserite-type bronze with formula Na_*x*_Pt_3_O_4_ (where 0 ≤ *x* ≤ 1).^50–52^ The central cuboidal {O_8_} cavity in [Pt^III^_12__8_(SO_4_)_12_]^4−^ is reminiscent of those of Pd-based cuboidal POMs, which are capable of incorporating magnetic heterometals.^21–27^ Similarly to the ε-Keggin, [Pt^III^_12_O_8_(SO_4_)_12_]^4−^ also exhibits a large number of multiple metal–metal bonded moieties, which is an unusual feature in POM chemistry (Fig. 1).

In 2007, the Musaev group proposed covalent dynamics involving Rh–Rh bonds in POMs based on isomerisation principles and DFT calculations.^53^ In 2016, it was reported that particular Pt-containing POMs could undergo redox responsive covalent dynamics, relevant for the design of nanosensors.^54^ The [Pt^II^_2_(PW_11_O_39_)_2_]^10−^ POM exhibits two square planar Pt^II^ centres that can undergo a two-electron oxidation process, forming [Pt^III^_2_(OH_2_)_2_(PW_11_O_39_)_2_]^8−^ species with a core {O–Pt–Pt–O} functional moiety.^54^ Upon two-electron reduction, the {O–Pt–Pt–O} can be dissociated while the system shifts between two different electronic states.^54^ Such covalent changes are well studied in organic chemistry, leading to many functional dynamic covalent systems.^55,56^ However, in many classical POMs, the bonding is established exclusively by metal–oxygen bonds. In such systems, covalent dynamics would imply reversible irreversible disintegration, which is not desirable. Nevertheless, designing POMs that can undergo covalent dynamics can be technologically important. In the context of electron storage, the Keggin structure α-[PMo_12_O_40_]^3−^ has been reported to undergo 24 electron reduction, where the storage of the electrons is enabled by the formation of Mo–Mo bonded triangles.^57,58^ Covalent dynamics and formation of Se–Se bonds in the Wells–Dawson archetype [Mo_18_O_54_(Se^IV^O_3_)_2_]^4−^ is another example where this concept has led to the discovery of memristive POM systems.^9^

In recent years, the deposition of POMs on conducting surfaces and their characterisation with SEM or AFM has become ubiquitous.^59^ Typically, POMs exhibit electron trapping properties, while their contact with metal substrates can affect the tunnelling mechanism in a way that leads to voltage plateaus and charge hysteresis.^60^ Despite the experimental effort, simulation of the electron transport properties, including spin-polarized transport in single POM-based junctions, has remained challenging.^61–65^ In an STM setup, a change of the electric field or the STM-tips can alter the electronic and spin states of molecular-based systems. In this regard, POMs as electronic or spintronic components may provide several advantages, among which the alteration of the overall charging of the POM and the oxidation state of individual metal centres with the help of the gate voltage is the most important. POMs that are spin polarised in their ground state can influence the transport without the need to employ external magnetic fields, while POMs built of nuclear-spin-free units and diluting magnetic centres can act as quantum computing units (*i.e.* qubits) with reduced quantum decoherence.

In the present article, we apply density functional theory (DFT) calculations to describe the structural, electronic and reactivity properties of [Pt_12_O_8_(SO_4_)_12_]^4−^, its adducts and derivatives. The studied model systems are carefully chosen to provide stability and reactivity trends and insights into the prospects of synthetically expanding this area. Our work also features modelling and DFT calculations of electron and spin-polarised transport in {Pt^III^_12_O_8_(SO_4_)_12_}-based junctions in the presence of applied bias and gate voltages.

## Models and methods

### Discrete polyanion models

The structure and the electronic properties of all polyanions were studied by taking into account relativistic effects, as implemented in the ADF 2018 code.^66,67^ The geometry optimization of all these polyanions was conducted at the BP86/TZP/ZORA/COSMO-water level. The BP86 functional has been proven to provide an accurate structure estimation for Au and Pd based POMs,^19–22,27,68^ and for Pt-containing POMs,^54^, and it is one of the most commonly applied functionals in the geometry study of classical POMs.^69^ To ensure the optimization energy minimum and provide a description of the IR spectrum of [Pt_12_O_8_(SO_4_)_12_]^4−^, we performed subsequent geometry optimization using level “good” Becke numerical integration as implemented in the ADF program.^70,71^ The geometry optimization of key structures obtained at the BP86 level can also be reproduced using the PBE functional level (see ESI Table 7†). However, consistent with previous systematic work on POMs,^69^ analyses of the electronic structure and the bonding energies were performed based on single-point calculations at the B3LYP/TZP/ZORA/COSMO-water level.

Wickleder's XRD studies of the crystalline (NH_4_)_4_[Pt_12_O_8_(SO_4_)_12_] indicated that the core [Pt_12_O_8_(SO_4_)_12_]^4−^ motif exhibits an *S*_6_ point group symmetry which may have been enforced by the interaction of two NH_4_^+^ cations in the solid state.^54^ In the absence of cations, [Pt_12_O_8_(SO_4_)_12_]^4−^ adopts an ideal *T*_h_ symmetry which we could confirm using the Symmol program as implemented in ADF. However, as *T*_h_ is not among the ADF-programmed symmetry point groups, the calculations are performed using the *D*_2h_ symmetry point group. This inevitable “symmetry-break” implies discrepancies in geometry parameters. However, our geometry calculations showed a negligible difference of only 0.001 Å discrepancy between the calculated interatomic *d*(O⋯O) distances that define the inner {O_8_} cuboid.

To verify the presence of different bonds, for a selected set of structures we have also calculated the Mayer bond multiplicity indices.^72,73^ Calculations of various bold multiplicity indices are readily available in the ADF software and have been previously applied to study a wide variety of inorganic complexes^74^ and POMs.^75^

### Electron transport calculations

The spin-polarized quantum transport calculations were performed using the non-equilibrium Green's function (NEGF) technique^76^ within the Keldysh formalism as implemented in the Atomistix ToolKit (ATK) package.^77^ The calculations have been done using the GGA/PBE functional^78^ with single-zeta plus polarization numerical basis sets. The performance of this functional compared to GGA/BP86 has also been probed and is shown in Fig. S5.† This level of analysis is quite common for electron transportation calculations along with different sets of molecular junction models.^79^ The kinetic energy cut-off of 150 Ryd was set for the self-consistent calculations. The Brillouin zone of the two-probe system was sampled using a 3 × 3 × 100 Monkhorst–Pack grid.^80^ The Poisson–Schrödinger equation of the system was self-consistently solved using a Fast Fourier Transform (FFT) solver. The transport models were treated as two-probe systems with the central scattering region sandwiched between the semi-infinite source (left) and drain (right) gold electrodes. In order to avoid any artificial effects from the contact between the electrode and the molecule, a small part of the electrodes, including a Au (111) slab with four metal layers, was considered in the channel region as a buffer. The central scattering part contains 368–376 atoms corresponding to a channel length of *ca.* 32 Å (see Table S7†). The energy window for the calculation of the transport properties was chosen in the range of (−2, 2) eV, within 101 points.

The whole central part was fully relaxed using the Broyden–Fletcher–Goldfarb–Shanno method, with the maximum force on each atom being less than 0.05 eV Å^−1^.

The spin-polarized current through the molecular junction under a finite bias voltage is calculated using the Landauer formula:^58^
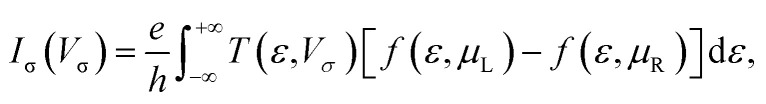
where *T*(*ε*,*V*_*σ*_) represents the spin-, energy- and voltage-resolved transmission function, *f* is the Fermi–Dirac distribution function, and *μ*_L,R_ = *E*_F_ ± *eV*_Bias_/2 stands for the chemical potentials of the left and right electrodes. The transmission function, which describes the probability of the carriers from the left to the right electrode, can be calculated *via* a self-consistent procedure.^81^ The influence of gate voltage is calculated by shifting the Hamiltonian elements of the channel region *via* a conversion into electrostatic potential energy. This assumes that the gate electrode generates an external voltage in the channel area. The transport calculations were performed at *T* = 300 K.

## Results and discussion

### Geometric properties and comparative analysis

**Fig. 2:**
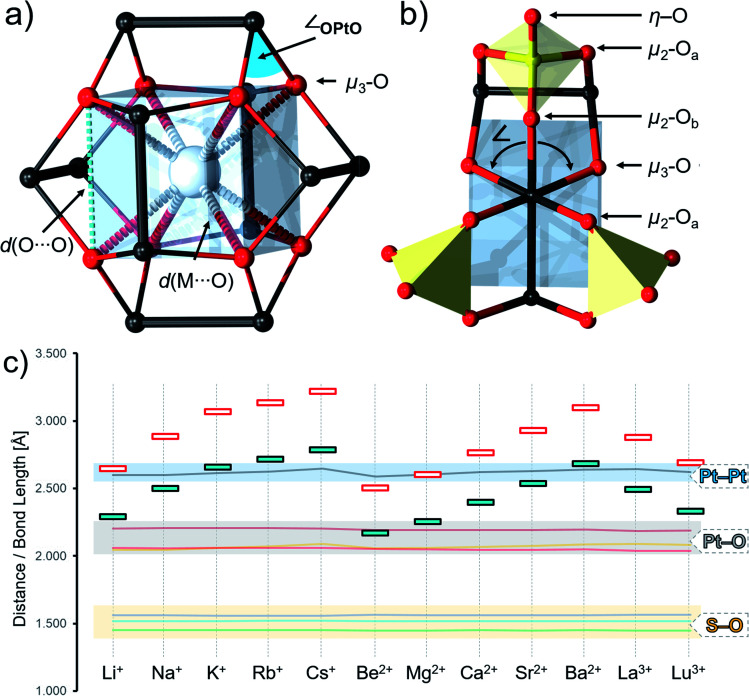
(a) The {Pt_12_O_8_}-core with markings of important interatomic distances. (b) Fragment of [Pt_12_O_8_(SO_4_)_12_]^4−^ depicting the structurally inequivalent oxo ligands. (c) Diagram showing the S–O, Pt–O, and Pt–Pt bond lengths and the interatomic *d*(O–O) and *d*(M–O) distances in [MPt_12_O_8_(SO_4_)_12_]^(4−*n*)−^ species where M^*n*^ is any of the listed metal cations. Combined ball and stick representation where Pt = black, O = red, S = yellow and M = grey spheres and {SO_4_} = yellow tetrahedra and {MO_8_} = blue cube.

The metal oxo skeletons of POMs often relate to fragments of extended solids and thus, they can be described as intermediate species in the metal oxide/hydroxide formation.^82–84^ For instance, many cuboidal and kegginoidal POMs relate to metal oxo bronzes, while the Anderson structure can relate to layered metal oxides/hydroxides.^1,17^ In the present article, to understand how virtual nanoconfinement affects the structure of [Pt_12_O_8_(SO_4_)_12_]^4−^, we compare the structural properties of [Pt_12_O_8_(SO_4_)_12_]^4−^ to those of the electron-conducting platinate bronze Na_*x*_Pt_3_O_4_. The crystallographically elucidated {Pt_12_O_8_} cores found in Na_*x*_Pt_3_O_4_ and [Pt_12_O_8_(SO_4_)_12_]^4−^ have some profound geometrical differences. In Na_*x*_Pt_3_O_4_, the Pt centres form {Pt⋯Pt···}_*n*_ chains with a closest interatomic *d*(Pt⋯Pt) distance of 2.844 Å. This distance also equals the interatomic distance between two μ_3_-O corners defining a single edge of the virtual {O_8_} cube (Fig. 2a and Table S1†). On the other hand, the interatomic *d*(Pt⋯Pt) distance in [Pt_12_O_8_(SO_4_)_12_]^4−^ is 2.532 Å, which is representative of the Pt^III^–Pt^III^ bond. In Na_*x*_Pt_3_O_4_, all Pt–O bond lengths are equivalent and equidistant, that is, *d*(Pt–O) = 2.011 Å. In [Pt_12_O_8_(SO_4_)_12_]^4−^, there are three structurally inequivalent O ligands and thus three different Pt–O bond lengths. Each Pt^III^ centre connects to two pairs of μ_3_-O ligands and μ_2_-O_*a*_ ligands originating from the central {O_8_} cube and two {SO_4_} units with a Pt–O bond length of 2.000 Å. Besides, each Pt^III^ axially connects to a single μ_2_-O_*b*_ ligand originating from a sulfate {SO_4_} unit through a Pt–O bond with a length of 2.016 Å (Fig. 2b). In Na_*x*_Pt_3_O_4_, the μ_3_-O ligands formulate perfectly trigonal planar nodes that connect to neighbouring Pt, virtually forming {Pt_3_O} moieties. The high-ordering also demands right angles between each Pt centre and each pair of neighbouring μ_3_-O ligands (*i.e.* ∠_in_ = 90.0°). In [Pt_12_O_8_(SO_4_)_12_]^4−^, on the other hand, the values of ∠_in_ increase to 92.3° while simultaneously displacing each of the eight μ_3_-O ligands away from the centre of the cube. Therefore, in [Pt_12_O_8_(SO_4_)_12_]^4−^, the μ_3_-O lay some 0.104 Å above the basal plane formed by the three neighbouring Pt^III^ centres, making the {Pt_3_O} “blunt” trigonal pyramids.

The geometry optimization of [Pt_12_O_8_(SO_4_)_12_]^4−^ (*D*_2h_) provided a reasonable estimation of the Pt–O bond lengths, which is only a minor overestimation of 0.07 Å for the equatorial and 0.03 Å for the axial Pt–O. Similarly, the optimization showed reliable estimation of the Pt^III^–Pt^III^ bond lengths at 2.582 Å and the three structurally inequivalent S–O bond lengths in the range of 1.453 Å to 1.553 Å. In this regard, the calculated ∠_in_ = 97.6° and consequently, μ_3_-O as one of the most basic sites with a high propensity for the solvent was calculated some 0.259 Å above the basal plane of neighbouring {Pt_3_} centres. When modelling a single Na^+^ within the central cavity of [Pt_12_O_8_(SO_4_)_12_]^4−^, the eight μ_3_-O ligands come close to the basal plane, ultimately leading to ∠_in_ = 89.7° and *d*(O⋯O) = 2.885 Å.

The conformational changes are geometrically significant, which motivated us to explore how the structure of [Pt_12_O_8_(SO_4_)_12_]^4−^ is altered when incorporating metal cations that differ in size and charge and how that compares to other classical cuboidal systems. In this regard, we have modelled alkali (Li^+^, Na^+^, K^+^, Rb^+^ and Cs^+^), alkaline earth (Be^2+^, Mg^2+^, Ca^2+^, Sr^2+^ and Ba^2+^) and lanthanide cations (La^3+^ and Lu^3+^) within the cavity of [Pt_12_O_8_(SO_4_)_12_]^4−^. The incorporated cations had a profound effect on *d*(O⋯O), *d*(M⋯O) and ∠_in_ = 89.7°, which are also mutually linearly correlated. These are lowest for Be^2+^ and largest for Cs^+^ and vary in the range of *d*(O⋯O) = 2.503–3.217 Å and ∠_in_ = 75.1°–100.8°. The incorporation of different cations neither affects the Pt–Pt bond length (0.06 Å deviation within the whole series), nor the Pt–O and S–O bonds (Fig. 2c). The largest contraction, as in the case of Be^2+^, reverses the orientation of the μ_3_-O, and it points towards the interior of the structure. Similar results and trends are obtained when substituting the sulphate groups with phosphate groups.

### Electronic properties and reactivity of the ground state

To understand the relevance of the structural and electronic changes as a function of the incorporated cations, we make inter-archetypal and intra-archetypal structural comparisons (see Scheme S1†). The optimized cuboidal structures [Pt_12_O_8_(SO_4_)_8_]^8−^ and [Pt_12_O_8_(PO_4_)_8_]^16−^ provide an opportunity for inter-archetypal comparisons. The calculated Pt^II^–O bond lengths in [Pt_12_O_8_(SO_4_)_8_]^8−^ are in the range of 1.976–2.134 Å, which compare well with the related cuboidal polyoxopalladates,^34^ while the calculated S–O bond lengths are in the range of 1.474–1.594 Å, in agreement with those in the [Pt_12_O_8_(SO_4_)_12_]^4−^ systems discussed above. Incorporation of the same set of metal cations in [Pt_12_O_8_(SO_4_)_8_]^8−^ showed *d*(O⋯O) = 2.531–3.079 Å and ∠_in_ = 79.5°–99.7°. Both ranges are significantly narrower than in [Pt_12_O_8_(SO_4_)_12_]^4−^; however, their interdependency remains linear. The lower range may be rationalized by the difference in the coordination of the μ_3_-O ligands and the coordination of the Pt centres. The related [Pt_12_O_8_(PO_4_)_8_]^16−^ showed very similar topological properties to [Pt_12_O_8_(SO_4_)_8_]^8−^ (Table S2†).

**Fig. 3:**
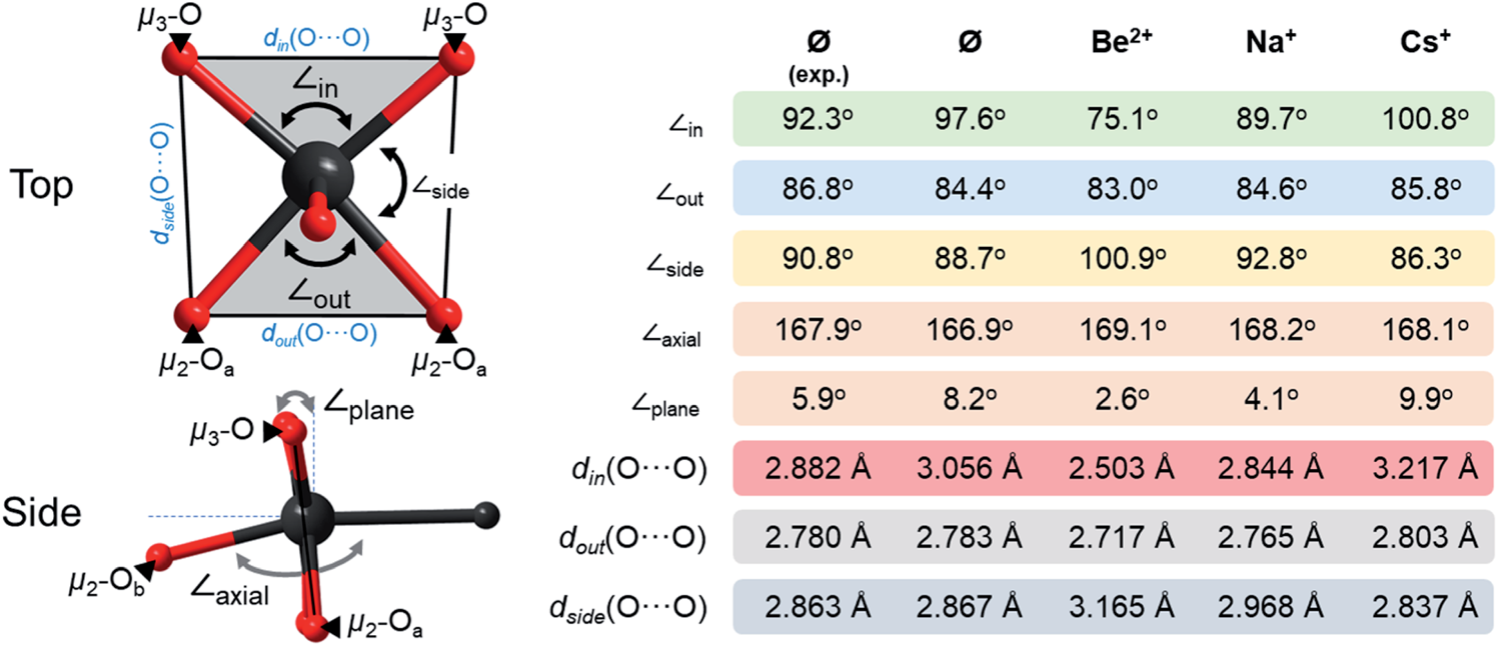
Seven different interatomic parameters describing the coordination geometry around the Pt^III^ centres. As Be^2+^, Na^+^ and Cs^+^ connect to the oxo ligands and not to Pt^III^, they have been omitted in the graphics on the left. Colour code: Pt = black and O = red spheres.

The intra-archetypal comparison in [Pt_12_O_8_(SO_4_)_12_]^4−^ derivatives requires the comparison of multiple structural parameters around every single Pt centre. The coordination around the Pt centres shows obvious deviations from the ideal octahedral complex. The O–Pt–Pt axes which lean at around 168°, instead of the ideal 180°, are an obvious example and indicative of intramolecular strains (see Fig. 3). Distortions are also found around the equatorial plane that features two structurally inequivalent O-ligands. The three O–Pt–O pointing towards the interior (∠_in_), peripheral (∠_side_), and outer side (∠_out_) are expected to add to 360° considering the planar alignment of the five atoms. As expansion in one angle *per se* leads to contraction of another, there is a certain deviation of about 15° from the ideal 90° and increased variance in the *d*(O⋯O) = 2.7–3.2 Å that arises when transitioning from small hard Be^2+^ cations to large and soft Cs^+^ cations. These distortions also affect the planarity of the Pt and its four equatorial O-ligands as expressed in virtual {PtO_4_} moieties. Virtual subdivision of the {PtO_4_} into two trigonal {PtO_2_} units aligning with ∠_in_ and ∠_out_ angles, respectively, suggests that the {PtO_4_} moiety itself is quasi-planar. This is evident from the interplanar angles that increase with the incorporation order: Be^2+^ (2.6°) < Na^+^ (4.1°) < Cs^+^ (9.9°). The distortions are one parameter that governs the incorporation, which is negative for Na^+^ (Δ*H* = −274.11 kJ mol^−1^) and Be^2+^ (Δ*H* = −1408.42 kJ mol^−1^) but positive for Cs^+^ (Δ*H* = 399.27 kJ mol^−1^) in [Pt_12_O_8_(SO_4_)_12_]^4−^.

### Electronic properties and reactivity of the ground state

**Fig. 4:**
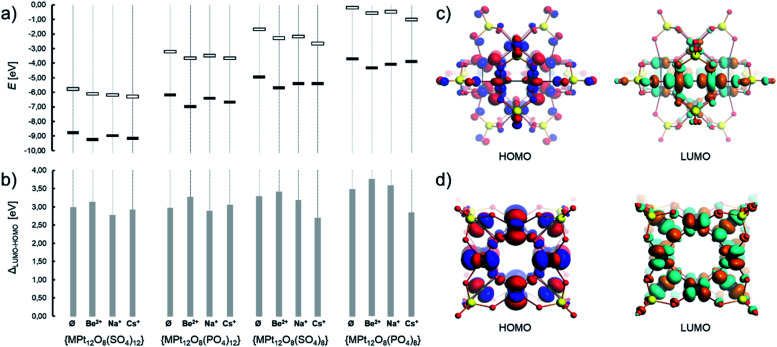
(a) Positioning of the HOMO and LUMO and (b) the respective gap energy of various {Pt_12_O_8_(XO_4_)_8_} and {Pt_12_O_8_(XO_4_)_12_} and their cation hosting systems {M⊂Pt_12_O_8_(XO_4_)_8_} and {M⊂Pt_12_O_8_(XO_4_)_12_}. Frontier orbitals of (c) [Pt_12_O_8_(SO_4_)_12_]^4−^ and (d) [Pt_12_O_8_(SO_4_)_8_]^8−^. All values are based on Table S3.† Colour code: Pt = black and O = red spheres. Isosurface value of MOs = 0.03 produced with a “fine” grid.

Electronic structure characterization, including the highest occupied and the lowest unoccupied molecular orbitals (*i.e.* HOMO and LUMO), and the respective gap energy (*i.e. Δ*_LUMO–HOMO_) provide a piece of intrinsic information on the reactivity and the stability of the involved POM complexes. In [Pt_12_O_8_(SO_4_)_12_]^4−^, *Δ*_LUMO–HOMO_ = 2.9 eV and this gap energy fluctuates slightly with the incorporation of the selected cations (see Fig. 4a and b), where, for instance, the gap energy [Na⊂Pt_12_O_8_(SO_4_)_12_]^3−^ is 2.8 eV. The evolution of the gap energies for different bare and cation containing systems (*i.e.* Be^2+^⊂POM, Na^+^⊂POM and Cs^+^⊂POM), in which POM = [Pt_12_O_8_(SO_4_)_12_]^*x*^ with *x* = −4, −8, and −16, is presented in Fig. 4b. The overall energy of the HOMO and LUMO increases with the negative charge while the formal configuration changes from d^7^ to d^8^. As a result, the *Δ*_LUMO–HOMO_ gap energy reaches 3.3 eV and 3.5 eV for [Pt_12_O_8_(SO_4_)_8_]^8−^ and [Pt_12_O_8_(PO_4_)_8_]^16−^, respectively. The latter gap energies are also comparable to those of cuboidal palladium(ii)-based POMs calculated on a comparable theoretical level.^68^

In [Pt_12_O_8_(SO_4_)_12_]^4−^, the HOMO mostly consists of O-centred p-type orbitals arranged in an antibonding fashion with Pt centres and Pt-centred d_*xy*_-like orbitals (*ca.* 33% distribution, see Table S4†) where the atom-centred orbitals are symmetrically tilted towards each other in a bonding fashion (Fig. 4c). On the other hand, the LUMO and LUMO+1 in [Pt_12_O_8_(SO_4_)_12_]^4−^ are both triply degenerate molecular orbitals of b.g and b.u type and consist of Pt-centred d_*z*^2^_ and d_*x*^2^−y^2^_ type orbitals (>65% distribution) arranged in an antibonding fashion, resembling molecular σ* and δ* orbitals characteristic of Pt–Pt bonded complexes. The addition of electrons in these orbitals suggests destabilization along the six {O–Pt–Pt–O} moieties (*vide infra*).^85–87^ The HOMO in [Pt^II^_12_O_8_(SO_4_)_8_]^8−^ and [Pt^II^_12_O_8_(PO_4_)_8_]^16−^ exhibits Pt-centred d_*xy*_-type and O-centred p-type atomic-like orbitals. The LUMO in the latter systems has an antibonding character along Pt–O bonds as it is based on Pt-centred d_*x*^2^−y^2^_-type and O-centred p-type atomic-like orbitals, suggesting destabilization upon further reduction (Fig. 4d).

The density isosurface of [Pt_12_O_8_(SO_4_)_12_]^4−^ and [Pt_12_O_8_(PO_4_)_12_]^16−^ exhibits continuity between each of the six Pt^III^-pair centres, which is in agreement with the existence of Pt–Pt bonds. On the other hand, the cation hosting complexes (*e.g.* [Na⊂Pt_12_O_8_(SO_4_)_12_]^3−^) show clear discontinuation of the electron density between the POM host and the cationic guest, which is indicative of ionic bonding. The plots of the molecular electrostatic potential (MEP) over the density isosurface reveal high basicity of the inner μ_3_-O ligands, which makes it comparable to those of some polyoxopalladates and polyoxoaurates.^15,19,20^ Mulliken charge analysis reveals the highest charge densities on the terminal oxo and the μ_3_-O oxo anions (Fig. S1a†).

**Fig. 5:**
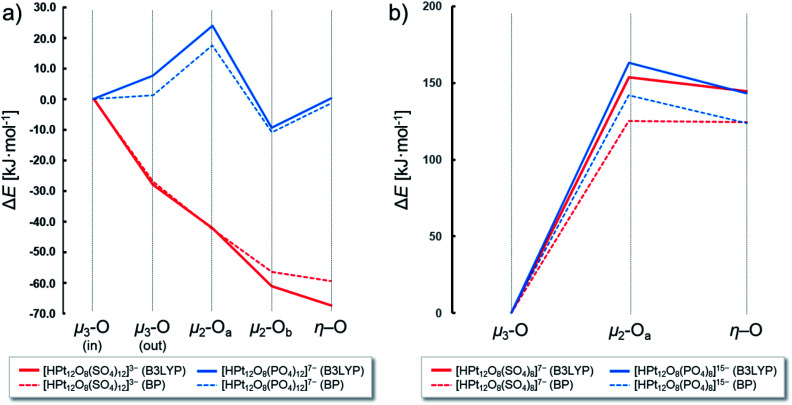
(a) Bonding energy difference relative to [HPt_12_O_8_(SO_4_)_12_]^3−^ (red) and [HPt_12_O_8_(PO_4_)_12_]^15−^ (blue) with protonation on one of the eight μ_3_-O ligands. (b) Bonding energy difference relative to [HPt_12_O_8_(SO_4_)_8_]^7−^ (red) and [HPt_12_O_8_(PO_4_)_8_]^15−^ (blue) with protonation on one of the eight μ_3_-O ligands. All values are based on Tables S5 and S6.† Calculations were performed using B3LYP (full line) and BP86 (dashed line).

In order to explore the reactivity of [Pt_12_O_8_(SO_4_)_12_]^4−^ and [Pt_12_O_8_(PO_4_)_12_]^16−^ and their related [Pt_12_O_8_(SO_4_)_8_]^8−^ and [Pt_12_O_8_(PO_4_)_8_]^16−^ towards protonation, we have used the simple reaction model: H_3_O^+^ + POM^*n*−^ → HPOM^(*n*+1)−^ + H_2_O. The calculated enthalpy indicates stabilization of the highly negative POMs in water and in the presence of protons, which is qualitatively expected for heteropolyacids. However, when comparing the two archetypes, there are important differences. In the mono-protonated [HPt_12_O_8_(SO_4_)_12_]^3−^ structure, the protonation of the terminal oxo positions is the most favoured, followed by the protonation of the axial oxo μ_2_-O_*b*_ with 6.2 kJ mol^−1^ higher energy. On the other hand, in the highly charged [HPt_12_O_8_(PO_4_)_12_]^15−^ the protonation is favoured on the axial oxo μ_2_-O_*b*_ position (by 9 kJ mol^−1^) in comparison with protonated structures at the terminal and the interior μ_3_ oxo ligands (Fig. 5a). In [HPt_12_O_8_(SO_4_)_8_]^8−^ and [HPt_12_O_8_(PO_4_)_8_]^16−^, the protonation is overwhelmingly preferred within the interior of the POM structure and lies >100 kJ mol^−1^ lower in energy than the terminal and bridging oxo ligands (Fig. 5b). The μ_3_-O oxo in the latter systems also exhibit the most negative Mulliken charges relative to the other ligands (Fig. S1b†). The large difference suggests higher reactivity of the central {O_8_} cavity within the cuboidal POMs in contrast to Wickleder's archetype. It should be mentioned that once the cavity is occupied, the terminal positions are next in line to be protonated, which is the case with cuboidal heteropolyoxopalladates^88^ and kegginoidal heteropolyoxovanadates.^89^

### Reduced states

In order to analyze the reduction effects on the electronic and geometric structure of [Pt_12_O_8_(SO_4_)_12_]^4−^, we have optimized the structure with the addition of six and twelve additional electrons leading to [Pt_12_O_8_(SO_4_)_12_]^10−^ and [Pt_12_O_8_(SO_4_)_12_]^16−^. The additional electrons are expected to fill the two triply degenerate LUMO and LUMO+1 orbitals (b.g and b.u type), following which, in the case of [Pt_12_O_8_(SO_4_)_12_]^10−^, we have explored both closed-shell (*i.e.* diamagnetic) and open-shell (*i.e.* high spin) configurations. However, our calculations showed that the optimized structure is about 44 kJ mol^−1^ more stable when considering a closed-shell scenario. We have also explored octa-potassium adducts of [Pt_12_O_8_(SO_4_)_12_]^4−^, [Pt_12_O_8_(SO_4_)_12_]^10−^ and [Pt_12_O_8_(SO_4_)_12_]^16−^, that is {K_8_[Pt_12_O_8_(SO_4_)_12_]}^4+^, {K_8_[Pt_12_O_8_(SO_4_)_12_]}^2−^ and {K_8_[Pt_12_O_8_(SO_4_)_12_]}^8−^.

**Fig. 6:**
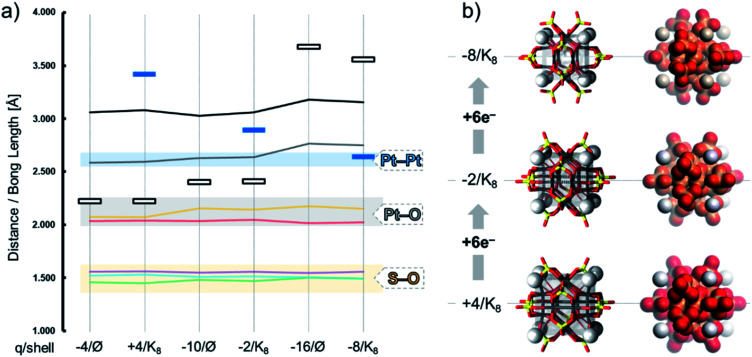
(a) Diagram showing the S–O, Pt–O, and Pt–Pt bond lengths and the interatomic *d*(O⋯O) (black line) and *d*(K⋯O) distances (blue rectangular markers) in {Pt_12_O_8_(SO_4_)_12_}^*q*^ (*q* = −4, −10 and −16) and {K_8_[Pt_12_O_8_(SO_4_)_12_]}^*q*^ (*q* = +4, −2 and −8). (b) Combined polyhedral/ball-and-stick and MEP representation of {K_8_[Pt_12_O_8_(SO_4_)_12_]}^*q*^ with red colour depicting the most basic positions. Colour code: Pt = black, O = red, S = yellow sticks and K = grey spheres; {K_8_} = grey cube. The density isosurface is produced using a “fine” grid and the isosurface value is 0.03.

The stepwise reduction shows that the bond lengths within the tetrahedral {SO_4_} and the quasi-planar {PtO_4_} moieties remain almost unaffected. However, in both bare and {K_8_}-adduct type models, there is a stepwise increase around the six axial {O–Pt–Pt–O} moieties. The distance between the Pt centre and the axial μ_2_-O_*b*_ increases from 2.219 Å (+0e^−^) to 2.397 Å (+6e^−^) and 3.674 Å (+12e^−^). In the final reduction stage, the twelve Pt–O bonds in [Pt_12_O_8_(SO_4_)_12_]^16−^ are dissociated, as shown by the large distance and clear absence of electron density. Consequently, the distance between the two Pt centres increases from 2.582 Å (+0e^−^) to 2.627 Å (+6e^−^) and then to 2.761 Å (+12e^−^) (see Fig. 6a). Apart from these geometric modifications, the remaining part of the structure stays well intact. In [Pt_12_O_8_(SO_4_)_12_]^16−^, the two Pt centres remain close in a distance range that can be explained by Pt⋯Pt contact or platinophilic interactions.^90^ The bond distances (*e.g.* Pt–O, S–O, and Pt–Pt) between bare “∅” and adduct “{K_8_}^8+^” models are virtually similar (Fig. 6a). However, as the charging of the POM progresses, the cationic {K_8_}^8+^ shell around the POM starts to contract, showing *d*(K⋯K) edge distances of 3.413 Å, 2.887 Å and 2.639 Å for “0e^−^”, “6e^−^” and “12e^−^” reduced states, respectively. The positioning of the cations and the POM is illustrated in Fig. 6b, along with the molecular electrostatic potential of these adducts. The contraction of the {K_8_}^8+^ is triggered by ionic bonding, which increases as a result of the higher overall charge and the local charging of the POM at different sites (Fig. S1c†).

**Fig. 7:**
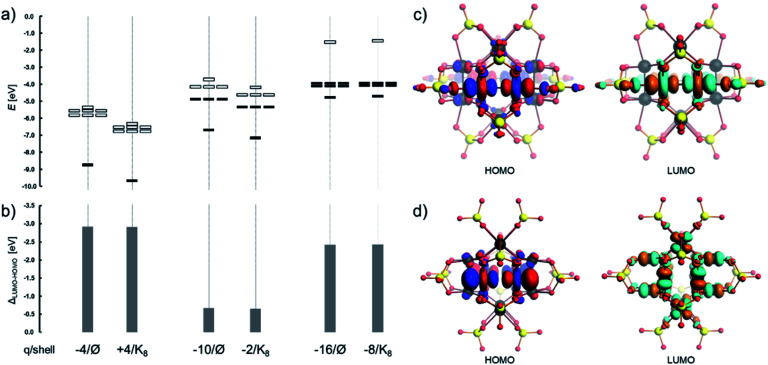
(a) Positioning of the HOMO and LUMO; (b) the gap energy, (c) frontier orbitals of [Pt_12_O_8_(SO_4_)_12_]^8−^, (d) frontier orbitals in [Pt_12_O_8_(SO_4_)_8_]^16−^. Colour code: Pt = black, O = red and S = yellow spheres. The MOs are produced using a “fine” grid and the isosurface value is 0.03.

The electronic structure of [Pt_12_O_8_(SO_4_)_12_]^*q*^ (*q* = −4, −8 and −12) species provides insights into the dissociation of the six {O–Pt–Pt–O} moieties. This is also evident from the output of the calculated Mayer bond valence indices (Fig. S1d†). In the case of reduction, the LUMO and LUMO+1 of [Pt_12_O_8_(SO_4_)_12_]^*n*−^, which are antibonding in character, become populated and thus constitute the new HOMO or HOMO−1 (Fig. 7a and b). In [Pt_12_O_8_(SO_4_)_12_]^10−^, the energy difference between the pair of triply degenerate HOMO and LUMO orbitals (Fig. 7c) is only 0.7 eV which is very low. This is not surprising as it implies that in the partially reduced and nanoconfined {Pt_12_O_8_}, many of the unoccupied and occupied orbitals are close in energy, while in the structurally related Na_*x*_Pt_3_O_4_ there is virtually no separation of electron populated and unpopulated band gaps, leading to electron-conducting behaviour. In [Pt_12_O_8_(SO_4_)_12_]^16−^ featuring six dissociated {O–Pt–Pt–O} moieties, the LUMO is allocated over Pt-centred *d*_*x*^2^−*y*^2^_-type and O-centred p-type atomic-like orbitals that are antibonding along with the remaining Pt–O bonds. The nature of the HOMO and LUMO in [Pt_12_O_8_(SO_4_)_12_]^16−^ (Fig. 7d) mimics that of [Pt_12_O_8_(SO_4_)_8_]^8−^ (Fig. 4d) and both structures exhibit reasonable stability based on their comparable *Δ*_LUMO–HOMO_ gap energies (*i.e.* 3.2 *vs.* 3.3 eV, respectively). The antibonding LUMO in [Pt_12_O_8_(SO_4_)_12_]^16−^ and [Pt_12_O_8_(SO_4_)_8_]^16−^ implies further dissociation of the equatorial Pt–O bonds, the start of the disintegration of the overall structure. Such description is analogous to the reduction in cuboidal polyoxopalladates. An interesting aspect between [Pt_12_O_8_(SO_4_)_12_]^16−^ and [Pt_12_O_8_(SO_4_)_8_]^16−^ is their possible interconversion. Using a model reaction [Pt^II^_12_O_8_(SO_4_)_12_]^16−^ → [Pt^II^_12_O_8_(SO_4_)_8_]^8−^ + 4 SO_4_^2−^ which describes a scenario of the release of four SO_4_^2−^ and internal rearrangement, we have calculated a difference of 3547 kJ mol^−1^ in advantage for [Pt_12_O_8_(SO_4_)_12_]^16−^. This implies that in aqueous media, [Pt_12_O_8_(SO_4_)_12_]^16−^ has higher thermodynamic stability than the products of rearrangement. Considering the slow kinetics of Pt^II^ complexes, rearrangements are also less likely to take place. These aspects show high promise for high electron reduction stability of [Pt_12_O_8_(SO_4_)_12_]^4−^ and its application in nanoelectronic devices.

The simultaneous dissociation of six {O–Pt–Pt–O} moieties is unusually rare and, to the best of our knowledge, an unparalleled combination of features by any other POM. During the opening of the POM (see the ESI video†), the twelve μ_3_-(SO_4_) capping groups in [Pt_12_O_8_(SO_4_)_12_]^4−^ turn into μ_2_-(SO_4_) groups in [Pt_12_O_8_(SO_4_)_12_]^16−^. In a related Pd-based POM, [Na_2_⊂Pd^II^_22_O_12_(As^V^O_4_)_16_]^26−^, there is a presence of a pair of static μ_2_-(As^V^O_4_) groups.^22^ High reduction in Keggin-type [PMo_12_O_40_]^3−^ to [PMo_12_O_40_]^12−^ leads to the formation of Mo–Mo bonds; however, on the basis of molecular dynamics calculations, it is shown that there is an unanticipated symmetry break and dissociation of a Mo–O bond.^58^ In the present case, the high reduction of [Pt_12_O_8_(SO_4_)_12_]^4−^ leads to a precise and controlled metal oxo bond dissociation which, to the best of our knowledge, is unprecedented in POM chemistry. In addition, frequency calculations show that the terminal S–O stretch undergoes a significant shift from 1210 cm^−1^ in [Pt_12_O_8_(SO_4_)_12_]^4−^ (Fig. S2a†) to 1064 cm^−1^ in [Pt_12_O_8_(SO_4_)_12_]^16−^ (Fig. S2b†), which indicates that such changes in structure can be spectroscopically detected.

**Fig. 8:**
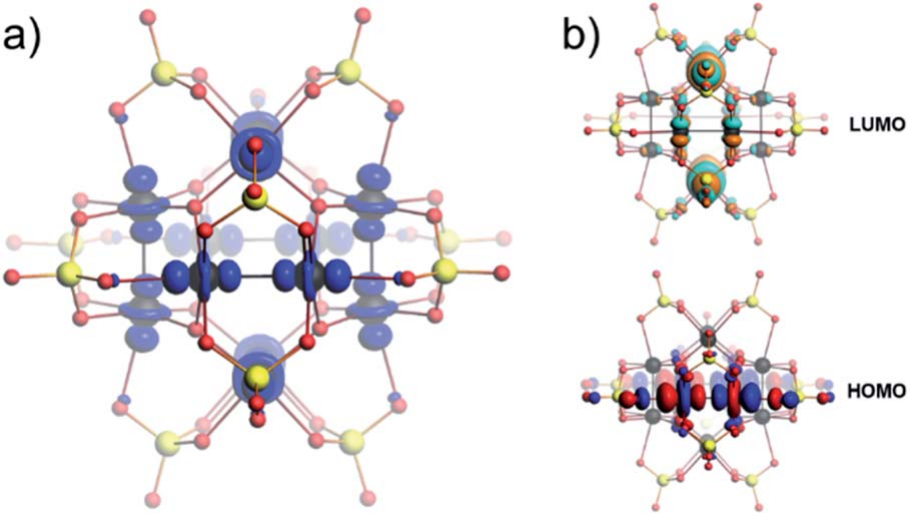
(a) Atomic spin density (ASD) isosurfaces indicating accumulation of α spins for the partially reduced [Pt_12_O_8_(SO_4_)_12_]^10−^. (b) Frontier orbitals in high-spin [Pt_12_O_8_(SO_4_)_12_]^10−^. Colour code: Pt = black, O = red, and S = yellow spheres. The MOs and spin density are produced using a “fine” grid and the isosurface value is 0.03.

The partially reduced [Pt_12_O_8_(SO_4_)_12_]^*n*−^ (*n* = 5–11) can lead to many spin-polarized scenarios and magnetic distributions, which may exhibit complicated spin–spin interactions. Such investigation relies on careful input from electrochemical and magnetochemical data which currently is beyond the scope of this work. In consideration of the applicable conformational changes present among POMs, what is also questionable is to what extent magnetic distributions may be topologically induced. To gain a qualitative understanding of the magnetic [Pt_12_O_8_(SO_4_)_12_]^8−^, we considered an unrestricted high-spin scenario (*i.e.* open-shell 6/2 spins). The atomic spin densities (ASDs) for [Pt_12_O_8_(SO_4_)_12_]^10−^ suggest that they are localized around the Pt centres and adopt the shape of *d*_*z*^2^_ atomic-like orbitals (Fig. 8a), reminiscent of the σ* orbital arrangement. We obtained an identical qualitative ASD distribution for the three-electron reduced system (*i.e.* [Pt_12_O_8_(SO_4_)_12_]^7−^). The frontier orbitals show similar qualitative distributions to the closed-shell systems described above (Fig. 8b).

### Transport properties of the Pt_12_S_12_ based device

**Fig. 9:**
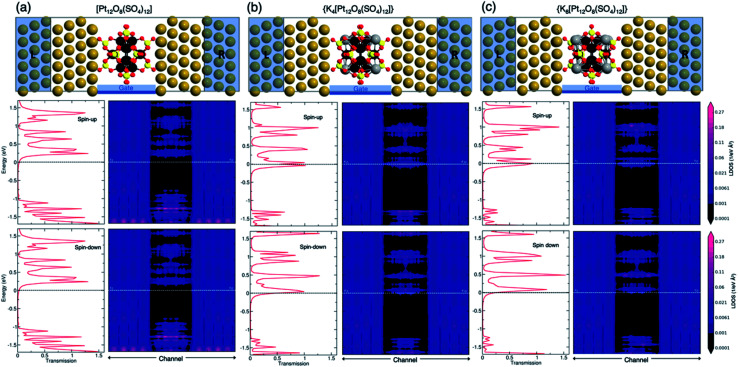
Schematic representation, zero-bias transmission spectra and energy resolved local device density of states (LDOS) for the Au/POM/Au junction with (a) [Pt_12_O_8_(SO_4_)_12_], (b) {K_4_[Pt_12_O_8_(SO_4_)_12_]} and (c) {K_8_[Pt_12_O_8_(SO_4_)_12_]}. L and R represent left and right semi-infinite electrodes, respectively. Colour code: Au = gold, Pt = black, O = red, S = yellow and K = grey spheres.

To further exploit the potential of the POMs as components of electronic devices, we studied the transport properties of {[Pt_12_O_8_(SO_4_)_12_]}, {K_4_[Pt_12_O_8_(SO_4_)_12_]} and {K_8_[Pt_12_O_8_(SO_4_)_12_]} based molecular junctions. For our purpose, the POM is placed between two bulk gold electrodes, the most common contact metal, cleaved in the [111] surface (Fig. 9a–c).

It is known that the coupling of the molecule to the electrode contact has a significant impact on the current flow in molecular electronic systems. Low contact resistance between molecules and the metal surface generally requires a favourable contact geometry, a high density of delocalized interface states at the Fermi level, and a low potential barrier.^91,92^ Our results indicate that the separation distance between the POM and Au surface is inversely proportional to the number of potassium cations, as shown in Table 1. The average distance between the Au surface and the {K_8_[Pt_12_O_8_(SO_4_)_12_]} molecule is 1.77 Å which is higher than that for the bare [Pt_12_O_8_(SO_4_)_12_] molecule (1.59 Å). This implies that the absence of potassium increases charge polarization at the POM–Au interface, which facilitates interfacial coupling between the two systems. Among the considered POMs, the smallest separation distance was found for the [Pt_12_O_8_(SO_4_)_12_]/Au interface corresponding to a binding energy of −0.18 eV Å^−2^.

**Table 1:** The distance of separation (*d*_POM–Au_) and binding energies between the POM and Au surface

System	{[Pt_12_O_8_(SO_4_)_12_]}	{K_4_[Pt_12_O_8_(SO_4_)_12_]}	{K_8_[Pt_12_O_8_(SO_4_)_12_]}
*d*_POM–Au_ (Å)	1.59	1.77	1.88
*E*_bind_ (eV Å^−2^)	−0.18	−0.14	−0.11

**Fig. 10:**
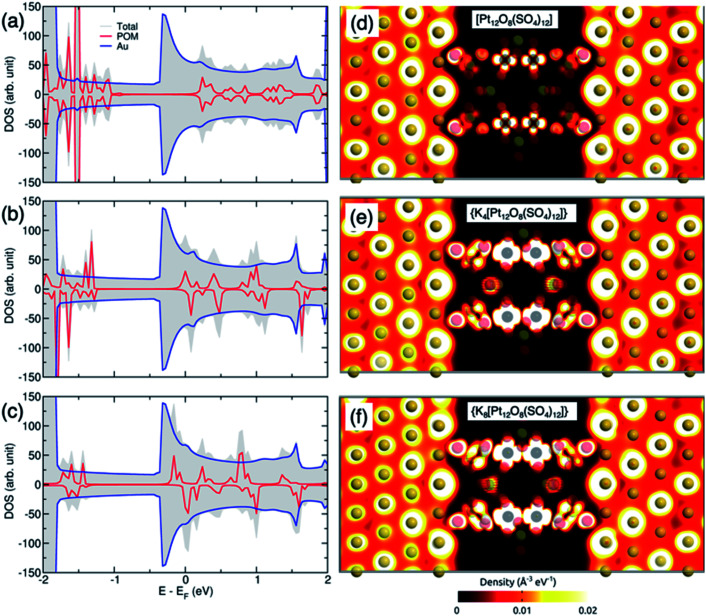
Density of states (DOS) for the Au/POM/Au junction with (a) [Pt_12_O_8_(SO_4_)_12_], (b) {K_4_[Pt_12_O_8_(SO_4_)_12_]} and (c) {K_8_[Pt_12_O_8_(SO_4_)_12_]}. (d–f) Electron density maps through cross-sections of the POM–Au based junctions.

The local density of states (LDOS) for the Au/POM/Au junction is shown in Fig. 9. The left and right sides of the plot show the metal electrode parts with a high density of states while the middle part indicates the distinct energy levels of the molecule coupled to the electrodes. A comparison of the LDOS and transmission spectra for the initial and reduced POM reveals that electron injection can also be modulated by reducing the POM. In addition, the coupling between the POM/POM-countercation adduct and the surface may depend on the direction of the molecule. We have also studied the effects of rotating the molecule around the transport axes (Fig. S3†). The results for three different orientations showed that the transmission spectra are almost independent of the rotation due to the high symmetry of the POM. Therefore, in the following, we only considered one configuration as typical. In the case of {[Pt_12_O_8_(SO_4_)_12_]}, the Fermi level is located in the HOMO–LUMO gap where no molecular orbital is available for electrons to tunnel through the molecule (see Fig. 9a). The incorporation of potassium countercations around the POMs introduces new transmission channels at the Fermi level (see Fig. 9b and c), which can contribute to electron injection through the molecule. The electron distribution indicates that the device, including {[Pt_12_O_8_(SO_4_)_12_]}, has the lowest electron population in the channel region and the average electron density at the channel increases with the number of modelled potassium atoms (Fig. 10).

The electronic structure around the Fermi level is primarily formed by Au 3d and Pt 3d orbitals with small contributions from O 2p orbitals (Fig. S4†). Therefore, these orbitals are mainly responsible for electron injection into the device. The presence of potassium countercations substantially enhances the electron density in the channel, as can be clearly seen both in the 2D density plot and in the available electronic states at the Fermi level.

It can be noted that the electronic densities inside the energy window are split for the spin-up and spin-down states. Since electrodes are made from a non-magnetic metal, it is clear that the spin-polarized transmission is entirely due to the magnetic properties of the POM molecule. The difference between the two spin subbands explains the localized magnetization in the POM-based channel. Note that the spin dependency of electronic states at the channel site mainly originates from Pt 3d and O 2p orbitals (Fig. S4†). The separation between the two spin subbands can provide two paths for electron conduction through the junction, as shown by the transmission spectrum in Fig. S3.†

**Fig. 11:**
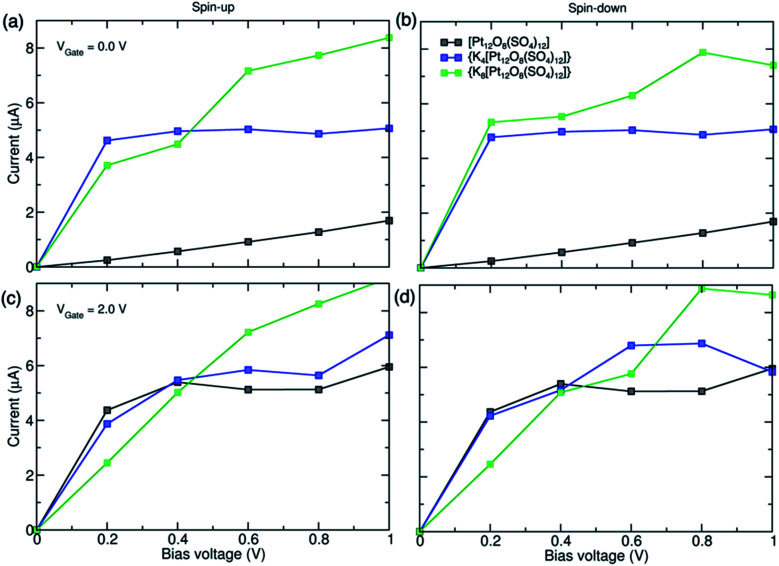
*I*–*V* characteristics for the Au/POM/Au junction with [Pt_12_O_8_(SO_4_)_12_], {K_4_[Pt_12_O_8_(SO_4_)_12_]} and {K_8_[Pt_12_O_8_(SO_4_)_12_]} under (a and b) *V*_Gate_ = 0.0 V and (c and d) *V*_Gate_ = 2.0 V. The spin-up and spin-down currents are shown in the left and right panels, respectively.

In order to investigate the transport features of the junction, we have calculated the *I*–*V* characteristics presented in Fig. 11. The *I*–*V* curves show a symmetrical behaviour with respect to *V*_Bias_ = 0.0 V; thus, the results for negative applied voltages are not shown. Since the currents depend on the molecular density of states lying between the left and right electrodes' chemical potentials, increasing the bias voltage makes more transport channels available inside the energy window, and currents increase. The *I*–*V* characteristics show an ohmic behaviour at low applied voltages and display different behaviours at higher voltages, depending on the reduction of the POM.

One interesting feature is that both spin up and down currents for the {K_4_[Pt_12_O_8_(SO_4_)_12_]} based device show a saturation region for bias voltages higher than 0.4 V. By applying a gate voltage, the POM is ionized due to the gate-induced shift in the energy of the molecular orbitals relative to the gold electrode's Fermi energy. For all studied cases, the current increases under the gate voltage as new conducting channels become available for the electrons to pass from the metal to the molecule. In the case of *V*_G_ = +2.0, the transmission coefficient of the gated device is higher than that of *V*_G_ = 0.0 V at *E*_F_, which increases the molecular conduction, leading to the enhanced *I*–*V* curves in Fig. 11.

## Conclusions

As nanoscopic metal-oxo clusters, POMs have a very broad range of applications. Most of the studied POMs belong to the group of early transition metals, and therefore most of the developed applications are related to that set of materials. In this article, we took the liberty to explore POMs made of late transition metals, in particular polyoxoplatinates. We also looked at polyoxoplatinates that exhibit metal–metal bonds, a feature that is not very common for classical and fully oxidised POMs.^38^ To date, late transition metal-based POMs have been highlighted as important models for understanding the catalytic activity of noble metal catalysts,^93^ and as potential precursors for the generation of metal clusters.^21,94^ As a result, most efforts until now were invested in cuboidal polyoxopalladates.^21–27^ In this work, we have described the structure of the pyritohedral [Pt^III^_12_O_8_(SO_4_)_12_]^4−^ and we compared it to that of the cuboidal POM archetype (common for polyoxopalladates) and to that of the catalytically active platinum-oxo bronzes. In comparison to the cuboidal POM archetype, the cuboidal {O_8_} cavity of [Pt^III^_12_O_8_(SO_4_)_12_]^4−^ and its phosphate derivative [Pt^III^_12_O_8_(PO_4_)_12_]^16−^ offer lower structural rigidity but a remarkable capability for incorporation of cations differing in charge and size. The electronic structure of [Pt^III^_12_O_8_(SO_4_)_12_]^4−^ shows two triply degenerate LUMOs that can accommodate and delocalize twelve electrons over the twelve Pt centres. The calculations of the reduced species show that the population of the LUMOs has an antibonding character, which eventually triggers the dissociation of the six {O–Pt–Pt–O} moieties in a controllable manner whilst retaining the structural integrity of the POM. In that sense, [Pt^III^_12_O_8_(SO_4_)_12_]^4−^ is a transformable POM-cluster hybrid that can act as an all-inorganic example of dynamic covalent chemistry. In comparison to the α-Keggin POMs where reduction induces metal–metal bonding, the polyoxoplatinate exhibits a reversed electron storage mechanism where the reduction dissociates the metal–metal bonding. In that regard, our calculations predict that the polyoxoplatinate can trap multiple electrons and act as an “electron sponge”. In addition to the ground and fully reduced state, a number of spin-unpaired [Pt_12_O_8_(SO_4_)_12_]^(4+*n*)−^ species may also exist, which can affect transport phenomena.

Reduction of the POM can trigger non-linear changes in the HOMO–LUMO gap, which shows potential for [Pt^III^_12_O_8_(SO_4_)_12_]^4−^ in “POMtronics”. Following this, we studied the electronic transport through various bare and adduct POM models sandwiched between two gold electrodes. It has been shown that the overall charge of the POM (counterbalanced by the presence of potassium cations) changes the transmission curves and the *I*–*V* characteristics significantly. Applying gate voltage on the POM molecule can further tune the electron conduction in POM-based molecular devices.

The theoretical study of [Pt^III^_12_O_8_(SO_4_)_12_]^4−^ suggests that the polyanions have prospects for future synthetic derivatizations (*e.g.* with phosphate heterogroups and metal cation guests) and magnetochemical investigations. Furthermore, the high symmetry of this polyanion, its established bulk synthesis, and its high thermal and redox stability provide great advantage when designing nanodevices based on [Pt^III^_12_O_8_(SO_4_)_12_]^4−^ POMs. The presence of {O–Pt–Pt–O} moieties in [Pt^III^_12_O_8_(SO_4_)_12_]^4−^ provides possibilities for controlled covalent dynamics, which currently is inaccessible for other POMs. Following the broad application of the kegginoid and cuboid type POMs, [Pt^III^_12_O_8_(SO_4_)_12_]^4−^ and its derivatives can be further envisioned as promising candidates for molecular spintronics.

## Conflicts of interest

There are no conflicts to declare.

## Supplementary Material

NA-003-D1NA00387A-s001

NA-003-D1NA00387A-s002
