# Supplementary material for: Polyoxoplatinates as covalently dynamic electron sponges and molecular electronics materials
Source: Nanoscale Adv. 2021 Aug 13;3(19):5663–75. doi: 10.1039/d1na00387a (PMC9417413; doi:10.1039/d1na00387a)
Supplement: NA-003-D1NA00387A-s001 [file NA-003-D1NA00387A-s001.pdf]

Electronic Supplementary Material (ESI) for Nanoscale Advances.
This journal is © The Royal Society of Chemistry 2021

Polyoxoplatinates as Covalently Dynamic Electron Sponges and Molecular Electronics Materials

Aleksandar Kondinski,[a,†]* Mahdi Ghorbani-Asl[b*]

a) Department of Chemical Engineering and Biotechnology, University of Cambridge, Philippa Fawcett Dr, Cambridge CB3 0AS, Cambridge, UK

b) Institute of Ion Beam Physics and Materials Research, Helmholtz-Zentrum Dresden-Rossendorf, 01328 Dresden, Germany

* Correspondance: Email: aleksandar@kondinski.com and mahdi.ghorbani@hzdr.de

† On the leave from KU Leuven.

Note on the creation and usage of the term POMtronics:

The term POMtronics has appeared in the literature in multiple instances,[1–3] however it is originally inspired from the term “MOFtronics”, where MOF is an abbreviation of a Metal-Organic Framework. “MOFtronics” was coined by Mark Allendorf and coworkers in 2014,[4] and further popularized by Xinliang Feng and coworkers over the past years.[5] As a hypernym MOFtronics refers to tailoring the electronic structure of MOFs towards the development of MOF-based (opto)electronics, spintronics, battery and supercapacitor devices and other technologies.[4,5]

In similar context, the term POMtronics is a hypernym referring to electronic structure engineering of POMs towards the realization of POM-based technologies in nanoelectronics, opto and photoelectronics, spintronics, battery, capacitor and various energy conversion materials.

Note on POM Archetypes:

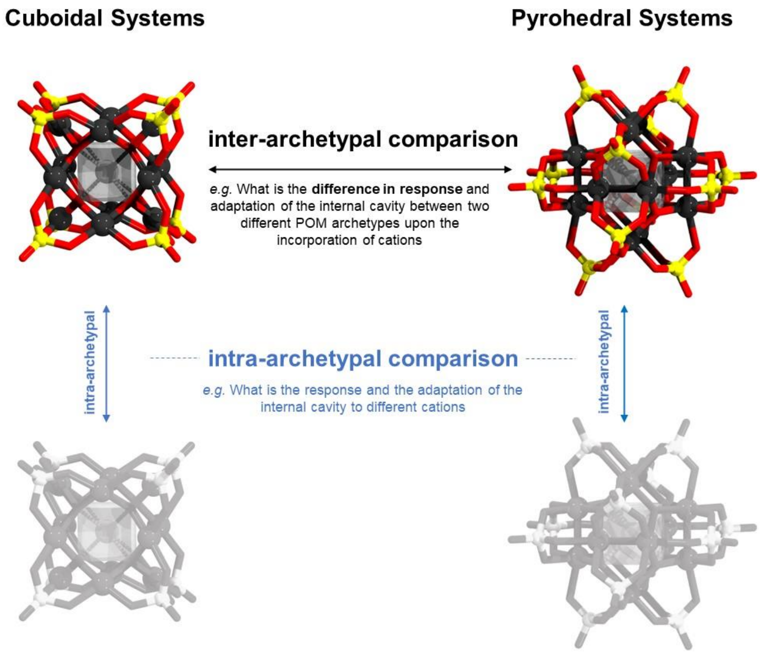

Scheme S1: Depiction of the difference between inter-archetypal comparisons and intra-archetypal comparisons. Both the cuboidal and pyrohedral dodecametalates share a number of similar features such as the set of building units (or building unit fragments), design of internal cavities etc. The intra-archetypal comparison offers an insight, for instance, on how the cavity of a particular archetype responds and re-adapts based on the incorporation of different metal cations. On the other hand, the inter-archetypal comparison offers insights into how this response and adaptation differs between different archetypes. Colour code: Pt = black, O = red, S = yellow, M = grey spheres.

Table S1: Calculated geometry parameters of $\{Pt_{12}O_8(XO_4)_{12}\}$ and cation bearing $\{M\subset Pt_{12}O_8(XO_4)_{12}\}$ based systems with X = S and P in comparison to crystallographically determined distances in reported structures. Geometry optimization at BP86/TZP/ZORA-scalar/COSMO-water level.

Structural Type	Bond lenghts and interatomic distances [Å]									$\angle_{in}$ [°]
	Pt−Pt	Pt−μ_3-(O)	Pt−O_{ax}	Pt−O_{eq}	S−O_t	S−O_b	S−O_b	d(O···O)	M···O	
$NaPt_3O_4$ (exp)[6]	2.844	2.011			-			2.844	2.463	90.0
$[Pt_{12}O_8(SO_4)_{12}]^{4-}$ (exp)[7]	2.532	2.000	2.14(2.16)	2.000	1.42(4)	1.460-1.482	1.521(6)	2.883	-	92.3
**$[Pt_{12}O_8(SO_4)_{12}]^{4-*}$**	**2.582**	**2.032**	**2.219**	**2.071**	**1.453**	**1.517**	**1.553**	**3.057**	**-**	**97.6**
$[Pt_{12}O_8(SO_4)_{12}]^{10-*}$	2.627	2.032	2.397	2.151	1.479	1.506	1.545	3.025	-	99.4
$[Pt_{12}O_8(SO_4)_{12}]^{16-*}$	2.761	2.012	3.674	2.169	1.499	1.501	1.540	3.175	-	104.3
$[K_8Pt_{12}O_8(SO_4)_{12}]^{4+*}$	2.588	2.036	2.217	2.068	1.444	1.525	1.557	3.076	-	98.2
$[K_8Pt_{12}O_8(SO_4)_{12}]^{2-*}$	2.632	2.044	2.408	2.138	1.465	1.511	1.552	3.058	-	96.9
$[K_8Pt_{12}O_8(SO_4)_{12}]^{8-*}$	2.744	2.020	3.553	2.149	1.489	1.491	1.553	3.153	-	102.8
$[Li\subset Pt_{12}O_8(SO_4)_{12}]^{3-}$	2.597	2.042	2.200	2.057	1.450	1.516	1.558	2.645	2.291	80.7
$[Na\subset Pt_{12}O_8(SO_4)_{12}]^{3-*}$	2.597	2.045	2.204	2.055	1.450	1.516	1.558	2.885	2.498	89.7
$[K\subset Pt_{12}O_8(SO_4)_{12}]^{3-}$	2.611	2.059	2.204	2.058	1.450	1.517	1.557	3.068	2.657	96.3
$[Rb\subset Pt_{12}O_8(SO_4)_{12}]^{3-}$	2.624	2.070	2.204	2.059	1.450	1518	1.556	3.134	2.714	98.4
$[Cs\subset Pt_{12}O_8(SO_4)_{12}]^{3-}$	2.645	2.088	2.202	2.059	1.450	1.518	1.556	3.217	2.786	100.8
$[Be\subset Pt_{12}O_8(SO_4)_{12}]^{2-*}$	2.586	2.054	2.192	2.050	1.447	1.515	1.562	2.503	2.168	75.1
$[Mg\subset Pt_{12}O_8(SO_4)_{12}]^{2-}$	2.602	2.060	2.192	2.048	1.447	1.516	1.561	2.602	2.253	78.3
$[Ca\subset Pt_{12}O_8(SO_4)_{12}]^{2-*}$	2.619	2.066	2.192	2.045	1.448	1.516	1.561	2.764	2.394	84.0
$[Sr\subset Pt_{12}O_8(SO_4)_{12}]^{2-}$	2.626	2.072	2.192	2.044	1.447	1.516	1.561	2.926	2.534	89.9
$[Ba\subset Pt_{12}O_8(SO_4)_{12}]^{2-}$	2.639	2.084	2.193	2.047	1.448	1.517	1.560	3.095	2.680	95.9
$[La\subset Pt_{12}O_8(SO_4)_{12}]^{-}$	2.641	2.087	2.184	2.035	1.445	1.516	1.564	2.877	2.492	87.1
$[Lu\subset Pt_{12}O_8(SO_4)_{12}]^{-}$	2.620	2.080	2.186	2.037	1.445	1.515	1.563	2.690	2.330	80.6
$[Pt_{12}O_8(PO_4)_{12}]^{16-*}$	2.609	2.044	2.225	2.069	1.531	1.566	1.591	3.063	-	97.0
$[Li\subset Pt_{12}O_8(PO_4)_{12}]^{15-}$	2.623	2.052	2.206	2.055	1.527	1.566	1.595	2.675	2.316	81.3
$[Na\subset Pt_{12}O_8(PO_4)_{12}]^{15-}$	2.627	2.057	2.208	2.052	1.527	1.566	1.595	2.906	2.517	89.9
$[K\subset Pt_{12}O_8(PO_4)_{12}]^{15-}$	2.639	2.072	2.205	2.055	1.527	1.566	1.594	3.079	2.666	95.9
$[Rb\subset Pt_{12}O_8(PO_4)_{12}]^{15-}$	2.652	2.083	2.205	2.056	1.527	1.567	1.594	3.143	2.721	97.9
$[Cs\subset Pt_{12}O_8(PO_4)_{12}]^{15-}$	2.673	2.101	2.204	2.055	1.527	1.568	1.594	3.221	2.791	100.2
$[Be\subset Pt_{12}O_8(PO_4)_{12}]^{14-}$	2.610	2.063	2.197	2.048	1.524	1.565	1.599	2.514	2.177	75.1
$[Mg\subset Pt_{12}O_8(PO_4)_{12}]^{14-}$	2.627	2.070	2.195	2.046	1.523	1.566	1.599	2.615	2.265	78.3
$[Ca\subset Pt_{12}O_8(PO_4)_{12}]^{14-}$	2.643	2.076	2.193	2.043	1.523	1.566	1.598	2.784	2.411	84.2
$[Sr\subset Pt_{12}O_8(PO_4)_{12}]^{14-}$	2.655	2.082	2.192	2.042	1.523	1.567	1.597	2.947	2.553	90.1
$[Ba\subset Pt_{12}O_8(PO_4)_{12}]^{14-}$	2.667	2.097	2.190	2.044	1.523	1.567	1.597	3.103	2.687	95.4
$[La\subset Pt_{12}O_8(PO_4)_{12}]^{13-}$	2.666	2.095	2.181	2.034	1.520	1.567	1.597	2.889	2.502	87.2
$[La\subset Pt_{12}O_8(PO_4)_{12}]^{13-}$	2.642	2.088	2.184	2.036	1.520	1.567	1.601	2.700	2.338	80.5

* Geometry has been provided in Table S7.

Table S2: Geometry parameters of $\{Pt_{12}O_8(XO_4)_8\}$ and cation bearing $\{M{\subset}Pt_{12}O_8(XO_4)_8\}$ based systems with X = S and P in BP86/TZP/ZORA-scalar/COSMO-water.

Structural Type	Bond lenghts and interatomic distances [Å]						$\angle_{in}$ [°]
	Pt−μ_3-(O)	Pt−O_{ax}	S−O_t	S−O_b	d(O···O)	M···O	
$[Pt_{12}O_8(SO_4)_8]^{8-*}$	1.976	2.134	1.474	1.545	2.645	-	84.0
$[Pt_{12}O_8(PO_4)_8]^{16-*}$	1.991	2.127	1.543	1.594	2.669	-	84.2
$[Li{\subset}Pt_{12}O_8(SO_4)_8]^{7-}$	1.983	2.119	1.470	1.546	2.629	2.277	83.1
$[Na{\subset}Pt_{12}O_8(SO_4)_8]^{7-}$	1.988	2.115	1.470	1.547	2.703	2.341	85.6
$[K{\subset}Pt_{12}O_8(SO_4)_8]^{7-}$	1.999	2.107	1.471	1.550	2.847	2.465	90.8
$[Rb{\subset}Pt_{12}O_8(SO_4)_8]^{7-}$	2.003	2.102	1.471	1.552	2.934	2.541	94.2
$[Cs{\subset}Pt_{12}O_8(SO_4)_8]^{7-}$	2.014	2.099	1.472	1.558	3.079	2.667	99.7
$[Be{\subset}Pt_{12}O_8(SO_4)_8]^{6-}$	1.979	2.112	1.466	1.547	2.531	2.192	79.5
$[Mg{\subset}Pt_{12}O_8(SO_4)_8]^{6-}$	1.989	2.108	1.466	1.548	2.615	2.265	82.2
$[Ca{\subset}Pt_{12}O_8(SO_4)_8]^{6-}$	2.001	2.102	1.466	1.549	2.734	2.367	86.2
$[Sr{\subset}Pt_{12}O_8(SO_4)_8]^{6-}$	2.008	2.097	1.467	1.551	2.828	2.449	89.5
$[Ba{\subset}Pt_{12}O_8(SO_4)_8]^{6-}$	2.016	2.093	1.467	1.553	2.943	2.549	93.8
$[La{\subset}Pt_{12}O_8(SO_4)_8]^{5-}$	2.014	2.088	13463	1.552	2.829	2.450	89.3
$[Lu{\subset}Pt_{12}O_8(SO_4)_8]^{5-}$	2.003	2.091	1.462	1.551	2.679	2.320	84.0
$[Li{\subset}Pt_{12}O_8(PO_4)_8]^{15-}$	1.997	2.111	1.539	1.595	2.648	2.293	83.0
$[Na{\subset}Pt_{12}O_8(PO_4)_8]^{15-}$	2.002	2.106	1.539	1.596	2.718	2.354	85.5
$[K{\subset}Pt_{12}O_8(PO_4)_8]^{15-}$	2.015	2.101	1.539	1.599	2.868	2.484	90.7
$[Rb{\subset}Pt_{12}O_8(PO_4)_8]^{15-}$	2.021	2.096	1.540	1.600	2.968	2.571	94.5
$[Cs{\subset}Pt_{12}O_8(PO_4)_8]^{15-}$	2.030	2.087	1.542	1.605	3.117	2.699	100.3
$[Be{\subset}Pt_{12}O_8(PO_4)_8]^{14-}$	1.992	2.101	1.534	1.597	2.541	2.200	79.2
$[Mg{\subset}Pt_{12}O_8(PO_4)_8]^{14-}$	2.004	2.097	1.534	1.597	2.628	2.276	82.0
$[Ca{\subset}Pt_{12}O_8(PO_4)_8]^{14-}$	2.014	2.092	1.535	1.599	2.747	2.379	86.0
$[Sr{\subset}Pt_{12}O_8(PO_4)_8]^{14-}$	2.023	2.089	1.535	1.600	2.845	2.464	89.4
$[Ba{\subset}Pt_{12}O_8(PO_4)_8]^{14-}$	2.032	2.083	1.536	1.602	2.966	2.569	93.8
$[La{\subset}Pt_{12}O_8(PO_4)_8]^{13-}$	2.029	2.076	1.529	1.602	2.842	2.461	89.9
$[La{\subset}Pt_{12}O_8(PO_4)_8]^{13-}$	2.018	2.081	1.530	1.600	2.693	2.332	83.7

* Geometry has been provided in Table S7.

Table S3: Energies of the HOMO, LUMO and the gap energies ($\Delta_{LUMO\text{-}HOMO}$) in eV for different systems obtained by single point calculations at B3LYP/TZP/ZORA-scalar/COSMO-water level.

	$\{M{\subset}Pt_{12}O_8(SO_4)_{12}\}$				$\{M{\subset}Pt_{12}O_8(PO_4)_{12}\}$				$\{M{\subset}Pt_{12}O_8(SO_4)_8\}$				$\{M{\subset}Pt_{12}O_8(PO_4)_8\}$			
	Ø	Be^{2+}	Na^+	Cs^+	Ø	Be^{2+}	Na^+	Cs^+	Ø	Be^{2+}	Na^+	Cs^+	Ø	Be^{2+}	Na^+	Cs^+
HOMO [eV]	-8.80	-9.29	-9.00	-9.23	-6.20	-6.96	-6.40	-6.70	-5.00	-5.71	-5.40	-5.39	-3.70	-4.33	-4.10	-3.88
LUMO [eV]	-5.80	-6.15	-6.20	-6.28	-3.20	-3.67	-3.50	-3.63	-1.70	-2.26	-2.20	-2.67	-0.20	-0.55	-0.50	-1.01
$\Delta_{LUMO\text{-}HOMO}$ [eV]	3.00	3.14	2.80	2.94	3.00	3.30	2.90	3.06	3.30	3.44	3.20	2.72	3.50	3.78	3.60	2.87

Table S4: Relative distributions of the HOMO, LUMO and the energy difference in different systems obtained by single point calculations at B3LYP/TZP/ZORA-scalar/COSMO-water level.

	$[Pt_{12}O_8(SO_4)_{12}]^{4-}$		$[K_8Pt_{12}O_8(SO_4)_{12}]^{4+}$		$[Pt_{12}O_8(SO_4)_{12}]^{8-}$		$[K_8Pt_{12}O_8(SO_4)_{12}]^{2-}$		$[Pt_{12}O_8(SO_4)_{12}]^{16-}$		$[K_8Pt_{12}O_8(SO_4)_{12}]^{8-}$	
	Pt	O	Pt	O	Pt	O	Pt	O	Pt	O	Pt	
HOMO [%]	33	64	26	69	74	18	74	18	86	13	86	10
LUMO [%]	66	23	66	24	73	19	72	20	73	19	72	17
$\Delta_{LUMO\text{-}HOMO}$ [%]	33	-40	40	-45	-2	1	-2	2	-13	6	-14	7

Table S5: Bonding energies of the monoprotonated $[HPt_{12}O_8(SO_4)_{12}]^{3-}$ and $[HPt_{12}O_8(PO_4)_{12}]^{15-}$ species calculated at BP86/TZP/ZORA-scalar/COSMO-water and B3LYP/TZP/ZORA-scalar/COSMO-water level.

Site of protonation	$[HPt_{12}O_8(SO_4)_{12}]^{3-}$				$[HPt_{12}O_8(PO_4)_{12}]^{15-}$			
	E^{BP86} [kJ·mol^{-1}]	E^{B3LYP} [kJ·mol^{-1}]	ΔE^{BP86} [kJ·mol^{-1}]	ΔE^{B3LYP} [kJ·mol^{-1}]	E^{BP86} [kJ·mol^{-1}]	E^{B3LYP} [kJ·mol^{-1}]	ΔE^{BP86} [kJ·mol^{-1}]	ΔE^{B3LYP} [kJ·mol^{-1}]
μ_3-O (in)	-46040,9	-57949,4	0	0	-56860,5	-69083,9	0	0
μ_3-O (out)	-46067,9	-57977,3	-27,07	-27,9	-56859,3	-69076,2	1,26	7,68
μ_2-O$_a$	-46083,3	-57991,6	-42,42	-42,2	-56843,1	-69060	17,46	23,9
μ_2-O$_b$	-46097,3	-58010,6	-56,44	-61,2	-56871,4	-69093,4	-10,92	-9,48
η–O	-46100,4	-58016,8	-59,54	-67,4	-56862	-69083,7	-1,49	0,18

Table S6: Bonding energies of the monoprotonated $[HPt_{12}O_8(SO_4)_8]^{7-}$ and $[HPt_{12}O_8(PO_4)_8]^{15-}$ species calculated at BP86/TZP/ZORA-scalar/COSMO-water and B3LYP/TZP/ZORA-scalar/COSMO-water level.

Site of protonation	$[HPt_{12}O_8(SO_4)_8]^{7-}$				$[HPt_{12}O_8(PO_4)_8]^{15-}$			
	E^{BP86} [kJ·mol^{-1}]	E^{B3LYP} [kJ·mol^{-1}]	ΔE^{BP86} [kJ·mol^{-1}]	ΔE^{B3LYP} [kJ·mol^{-1}]	E^{BP86} [kJ·mol^{-1}]	E^{B3LYP} [kJ·mol^{-1}]	ΔE^{BP86} [kJ·mol^{-1}]	ΔE^{B3LYP} [kJ·mol^{-1}]
μ_3-O	-36560,6	-45599,4	0	0	-43078,2	-52234,3	0	0
μ_2-O	-36435,6	-45445,8	125,05	153,56	-42936,3	-52071	141,98	163,31
η–O	-36436,2	-45454,8	124,4	144,61	-42954,5	-52091,3	123,7	143,05

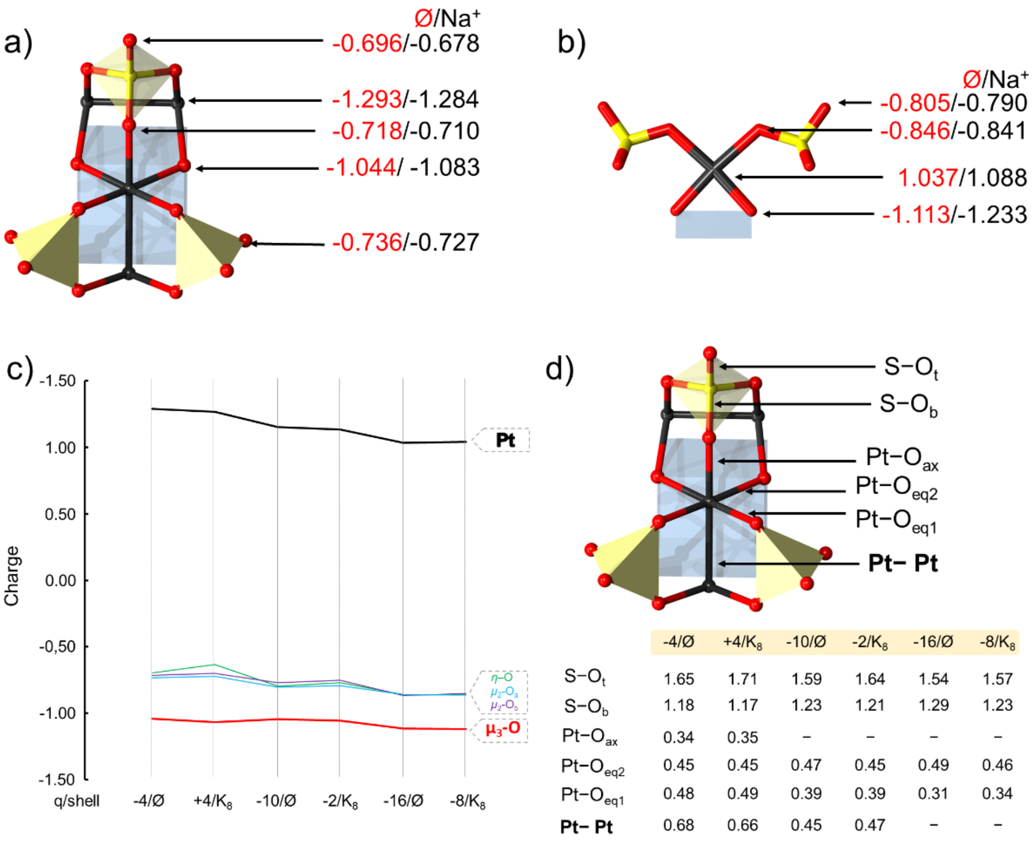

	-4/Ø	+4/K$_8$	-10/Ø	-2/K$_8$	-16/Ø	-8/K$_8$
S−O$_t$	1.65	1.71	1.59	1.64	1.54	1.57
S−O$_b$	1.18	1.17	1.23	1.21	1.29	1.23
Pt−O$_{ax}$	0.34	0.35	–	–	–	–
Pt−O$_{eq2}$	0.45	0.45	0.47	0.45	0.49	0.46
Pt−O$_{eq1}$	0.48	0.49	0.39	0.39	0.31	0.34
**Pt− Pt**	0.68	0.66	0.45	0.47	–	–

Figure S1. a) Mulliken charge populations of fractions of $[Pt_{12}O_8(SO_4)_{12}]^{4-}$/$[NaPt_{12}O_8(SO_4)_{12}]^{3-}$; b) Mulliken charge populations of fractions of $[Pt_{12}O_8(SO_4)_8]^{8-}$/$[NaPt_{12}O_8(SO_4)_8]^{7-}$; c) Mulliken charge populations at different oxo sites in $\{Pt_{12}O_8(SO_4)_{12}\}$/$\{K_8Pt_{12}O_8(SO_4)_{12}\}$ systems as a function of the overall charge; d) Typical Mayer bond multiplicity indices as calculated for $\{Pt_{12}O_8(SO_4)_{12}\}^q$ (q = -4, -10 and -16) and $\{K_8[Pt_{12}O_8(SO_4)_{12}]\}^q$ (q = +4, -2 and -8) models. Bond multiplicity indices lower 0.2 are by default not printed (i.e. omitted).

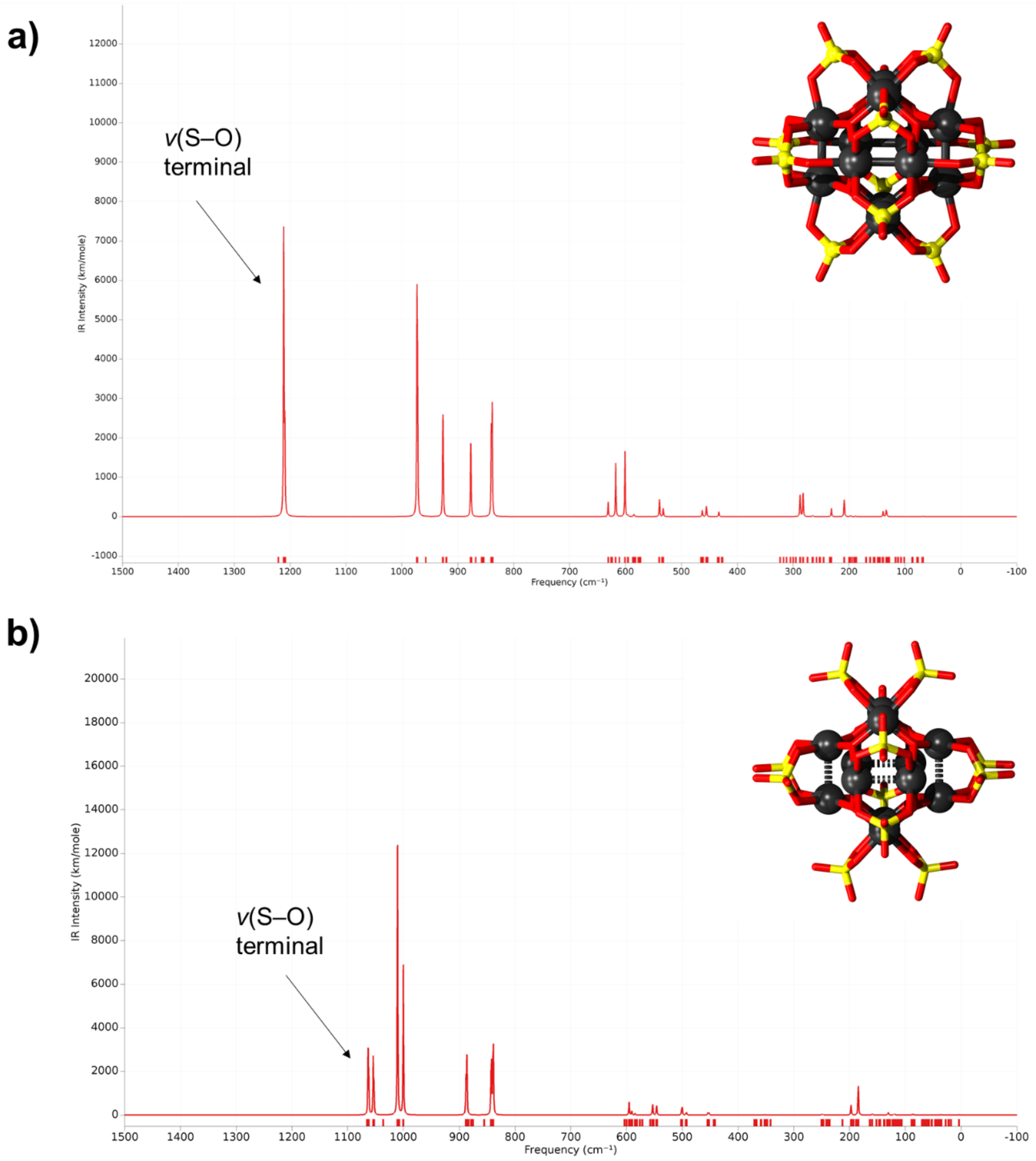

Figure S2: Calculated spectrum of a) $[Pt_{12}O_8(SO_4)_{12}]^{4-}$ and b) $[Pt_{12}O_8(SO_4)_{12}]^{16-}$ at BP86/TZP/ZORA/COSMO-water level.

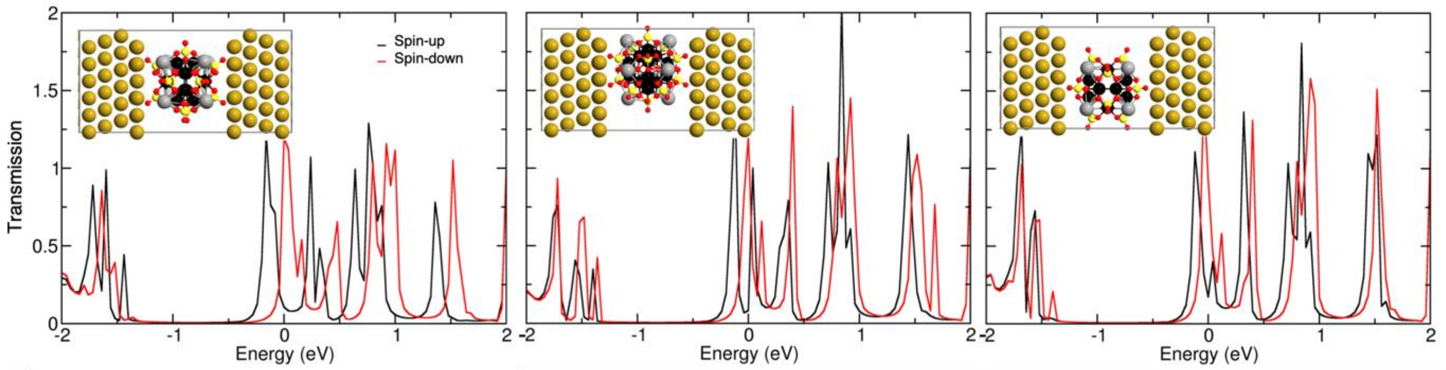

Figure S3. The zero-bias transmission spectra of the POM-based junction with 8 potassium for three different POM orientations. The spin up and spin down coefficients are shown with black and red, respectively. Inset shows the optimized geometry of the model.

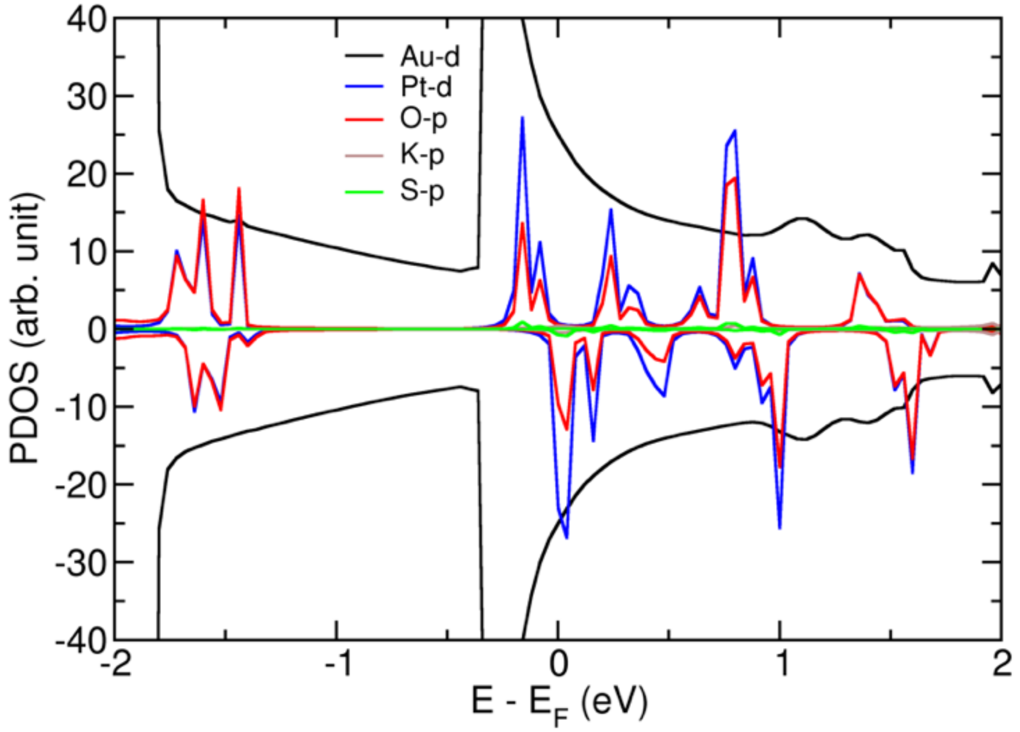

Figure S4: Projected density of states (PDOS) for the POM-based junction with $\{K_8[Pt_{12}O_8(SO_4)_{12}]\}$.

Figure S5: The comparison between transmission through the Au/POM/Au junction with $\{K_8[Pt_{12}O_8(SO_4)_{12}]\}$ calculated using PBE and BP86 functional.

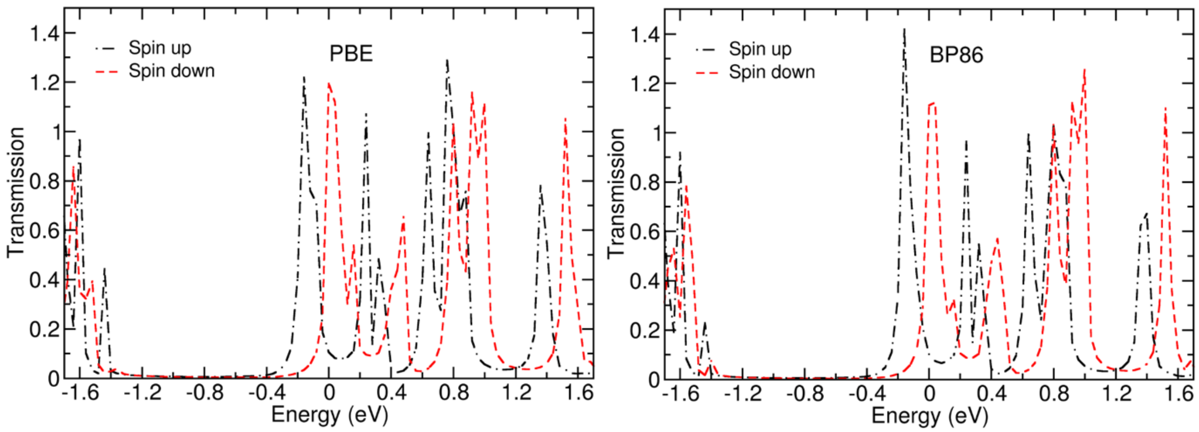

Table S7: Cartesian coordinates (in Angstroms) of the individual POMs and the Au/POM/Au junctions

$[Pt_{12}O_8(SO_4)_{12}]^{4-}$ (D_{2h})
Level: PBE/TZP/ZORA-scalar/COSMO-water

80

Pt	2.84335273	1.28964133	0.00000000
Pt	0.00000000	2.84335273	-1.28964133
Pt	1.28964133	0.00000000	-2.84335273
Pt	1.28964133	0.00000000	2.84335273
Pt	0.00000000	2.84335273	1.28964133
Pt	-1.28964133	0.00000000	-2.84335273
Pt	-2.84335273	1.28964133	-0.00000000
Pt	2.84335273	-1.28964133	-0.00000000
Pt	0.00000000	-2.84335273	-1.28964133
Pt	0.00000000	-2.84335273	1.28964133
Pt	-1.28964133	0.00000000	2.84335273
Pt	-2.84335273	-1.28964133	-0.00000000
S	4.53666864	0.00000000	-2.29180522
S	4.53666864	0.00000000	2.29180522
S	2.33552455	4.53666864	0.00000000
S	-2.29180522	4.53666864	-0.00000000
S	0.00000000	2.29180522	-4.53666864
S	0.00000000	-2.29180522	-4.53666864
S	0.00000000	2.29180522	4.53666864
S	0.00000000	-2.29180522	4.53666864
S	-4.53666864	0.00000000	-2.29180522
S	-4.53666864	0.00000000	2.29180522
S	2.29180522	-4.53666864	-0.00000000
S	-2.29180522	-4.53666864	-0.00000000
O	1.52704430	1.52704430	-1.52704430
O	1.52704430	1.52704430	1.52704430
O	4.37618377	1.25236284	-1.39047108
O	4.37618377	1.25236284	1.39047108
O	3.34707755	3.44938190	-0.00000000
O	-1.52704430	1.52704430	-1.52704430
O	-1.39047108	4.37618377	-1.25236284
O	1.39047108	4.37618377	-1.25236284
O	0.00000000	3.34707755	-3.44938190
O	1.52704430	-1.52704430	-1.52704430

O	3.44938190	0.00000000	-3.34707755
O	1.25236284	-1.39047108	-4.37618377
O	1.25236284	1.39047108	-4.37618377
O	1.52704430	-1.52704430	1.52704430
O	1.25236284	1.39047108	4.37618377
O	1.25236284	-1.39047108	4.37618377
O	3.44938190	0.00000000	3.34707755
O	-1.52704430	1.52704430	1.52704430
O	0.00000000	3.34707755	3.44938190
O	-1.39047108	4.37618377	1.25236284
O	1.39047108	4.37618377	1.25236284
O	4.37618377	-1.25236284	-1.39047108
O	5.85938705	0.00000000	-2.89098224
O	4.37618377	-1.25236284	1.39047108
O	5.85938705	0.00000000	2.89098224
O	2.89098224	5.85938705	-0.00000000
O	-1.52704430	-1.52704430	-1.52704430
O	-1.25236284	-1.39047108	-4.37618377
O	-1.25236284	1.39047108	-4.37618377
O	-3.44938190	0.00000000	-3.34707755
O	-3.34707755	3.44938190	-0.00000000
O	-4.37618377	1.25236284	1.39047108
O	-4.37618377	1.25236284	-1.39047108
O	-2.89098224	5.85938705	-0.00000000
O	0.00000000	2.89098224	-5.85938705
O	3.34707755	-3.44938190	-0.00000000
O	0.00000000	-3.34707755	-3.44938190
O	1.39047108	-4.37618377	-1.25236284
O	-1.39047108	-4.37618377	-1.25236284
O	0.00000000	-2.89098224	-5.85938705
O	-1.52704430	-1.52704430	1.52704430
O	1.39047108	-4.37618377	1.25236284
O	-1.39047108	-4.37618377	1.25236284
O	0.00000000	-3.34707755	3.44938190
O	-1.25236284	1.39047108	4.37618377
O	0.00000000	2.89098224	5.85938705
O	-1.25236284	-1.39047108	4.37618377
O	0.00000000	-2.89098224	5.85938705
O	-3.44938190	0.00000000	3.34707755
O	-4.37618377	-1.25236284	1.39047108
O	-4.37618377	-1.25236284	-1.39047108
O	-3.34707755	-3.44938190	-0.00000000
O	-5.85938705	0.00000000	-2.89098224
O	-5.85938705	0.00000000	2.89098224
O	2.89098224	-5.85938705	-0.00000000
O	-2.89098224	-5.85938705	-0.00000000

$[Pt_{12}O_8(SO_4)_{12}]^{10-}$ (D_{2h})
Level: PBE/TZP/ZORA-scalar/COSMO-water

80

Pt	2.84823608	1.31680789	0.00000000
Pt	0.00000000	2.84755372	-1.31669490
Pt	1.31683265	0.00000000	-2.84794548
Pt	1.31683265	0.00000000	2.84794548
Pt	0.00000000	2.84755372	1.31669490
Pt	-1.31683265	0.00000000	-2.84794548
Pt	-2.84823608	1.31680789	-0.00000000
Pt	2.84823608	-1.31680789	-0.00000000
Pt	0.00000000	-2.84755372	-1.31669490
Pt	0.00000000	-2.84755372	1.31669490

Pt	-1.31683265	0.00000000	2.84794548
Pt	-2.84823608	-1.31680789	-0.00000000
S	4.67051843	0.00000000	-2.29060051
S	4.67051843	0.00000000	2.29060051
S	2.29034065	4.67057669	-0.00000000
S	-2.29034065	4.67057669	-0.00000000
S	0.00000000	2.29084185	-4.66995423
S	0.00000000	-2.29084185	-4.66995423
S	0.00000000	2.29084185	4.66995423
S	0.00000000	-2.29084185	4.66995423
S	-4.67051843	0.00000000	-2.29060051
S	-4.67051843	0.00000000	2.29060051
S	2.29034065	-4.67057669	-0.00000000
S	-2.29034065	-4.67057669	-0.00000000
O	1.51947923	1.51917589	-1.51922019
O	1.51947923	1.51917589	1.51922019
O	4.45268435	1.25558096	-1.42019508
O	4.45268435	1.25558096	1.42019508
O	3.42794313	3.69008047	-0.00000000
O	-1.51947923	1.51917589	-1.51922019
O	-1.41989431	4.45186219	-1.25549821
O	1.41989431	4.45186219	-1.25549821
O	0.00000000	3.42791763	-3.68876330
O	1.51947923	-1.51917589	-1.51922019
O	3.68873183	0.00000000	-3.42735872
O	1.25533370	-1.42013248	-4.45201014
O	1.25533370	1.42013248	-4.45201014
O	1.51947923	-1.51917589	1.51922019
O	1.25533370	1.42013248	4.45201014
O	1.25533370	-1.42013248	4.45201014
O	3.68873183	0.00000000	3.42735872
O	-1.51947923	1.51917589	1.51922019
O	0.00000000	3.42791763	3.68876330
O	-1.41989431	4.45186219	1.25549821
O	1.41989431	4.45186219	1.25549821
O	4.45268435	-1.25558096	-1.42019508
O	6.06805317	0.00000000	-2.76876453
O	4.45268435	-1.25558096	1.42019508
O	6.06805317	0.00000000	2.76876453
O	2.76624244	6.06890449	-0.00000000
O	-1.51947923	-1.51917589	-1.51922019
O	-1.25533370	-1.42013248	-4.45201014
O	-1.25533370	1.42013248	-4.45201014
O	-3.68873183	0.00000000	-3.42735872
O	-3.42794313	3.69008047	-0.00000000
O	-4.45268435	1.25558096	1.42019508
O	-4.45268435	1.25558096	-1.42019508
O	-2.76624244	6.06890449	-0.00000000
O	0.00000000	2.76815580	-6.06780463
O	3.42794313	-3.69008047	-0.00000000
O	0.00000000	-3.42791763	-3.68876330
O	1.41989431	-4.45186219	-1.25549821
O	-1.41989431	-4.45186219	-1.25549821
O	0.00000000	-2.76815580	-6.06780463
O	-1.51947923	-1.51917589	1.51922019
O	1.41989431	-4.45186219	1.25549821
O	-1.41989431	-4.45186219	1.25549821
O	0.00000000	-3.42791763	3.68876330
O	-1.25533370	1.42013248	4.45201014
O	0.00000000	2.76815580	6.06780463
O	-1.25533370	-1.42013248	4.45201014
O	0.00000000	-2.76815580	6.06780463

O	-3.68873183	0.00000000	3.42735872
O	-4.45268435	-1.25558096	1.42019508
O	-4.45268435	-1.25558096	-1.42019508
O	-3.42794313	-3.69008047	-0.00000000
O	-6.06805317	0.00000000	-2.76876453
O	-6.06805317	0.00000000	2.76876453
O	2.76624244	-6.06890449	-0.00000000
O	-2.76624244	-6.06890449	-0.00000000

$[Pt_{12}O_8(SO_4)_{12}]^{16-}$ (D_{2h})
Level: PBE/TZP/ZORA-scalar/COSMO-water

80

Pt	2.79584499	1.37306537	0.00000000
Pt	0.00000000	2.80472997	-1.38062768
Pt	1.37789623	-0.00000000	-2.80888912
Pt	1.37789623	-0.00000000	2.80888912
Pt	0.00000000	2.80472997	1.38062768
Pt	-1.37789623	0.00000000	-2.80888912
Pt	-2.79584499	1.37306537	-0.00000000
Pt	2.79584499	-1.37306537	-0.00000000
Pt	0.00000000	-2.80472997	-1.38062768
Pt	0.00000000	-2.80472997	1.38062768
Pt	-1.37789623	0.00000000	2.80888912
Pt	-2.79584499	-1.37306537	-0.00000000
S	5.06311526	-0.00000000	-2.01280589
S	5.06311526	-0.00000000	2.01280589
S	2.01204053	5.06812424	0.00000000
S	-2.01204053	5.06812424	-0.00000000
S	0.00000000	2.01280263	-5.07350758
S	0.00000000	-2.01280263	-5.07350758
S	0.00000000	2.01280263	5.07350758
S	0.00000000	-2.01280263	5.07350758
S	-5.06311526	0.00000000	-2.01280589
S	-5.06311526	0.00000000	2.01280589
S	2.01204053	-5.06812424	-0.00000000
S	-2.01204053	-5.06812424	-0.00000000
O	1.58436044	1.58614612	-1.58990881
O	1.58436044	1.58614612	1.58990880
O	4.37840455	1.27262594	-1.48603518
O	4.37840455	1.27262594	1.48603517
O	3.50990426	4.98382724	-0.00000000
O	-1.58436044	1.58614612	-1.58990881
O	-1.48218324	4.38799212	-1.27434060
O	1.48218324	4.38799212	-1.27434060
O	0.00000000	3.51099710	-4.99125017
O	1.58436044	-1.58614612	-1.58990881
O	4.98846014	-0.00000000	-3.51145706
O	1.27379172	-1.48376869	-4.39193142
O	1.27379172	1.48376869	-4.39193142
O	1.58436044	-1.58614612	1.58990880
O	1.27379172	1.48376869	4.39193141
O	1.27379172	-1.48376869	4.39193141
O	4.98846014	-0.00000000	3.51145706
O	-1.58436044	1.58614612	1.58990880
O	0.00000000	3.51099710	4.99125017
O	-1.48218324	4.38799212	1.27434060
O	1.48218324	4.38799212	1.27434060
O	4.37840455	-1.27262594	-1.48603518
O	6.49724603	-0.00000000	-1.57905017
O	4.37840455	-1.27262594	1.48603517

O	6.49724603	-0.00000000	1.57905017
O	1.58648910	6.50446848	-0.00000000
O	-1.58436044	-1.58614612	-1.58990881
O	-1.27379172	-1.48376869	-4.39193142
O	-1.27379172	1.48376869	-4.39193142
O	-4.98846014	0.00000000	-3.51145706
O	-3.50990426	4.98382724	-0.00000000
O	-4.37840455	1.27262594	1.48603517
O	-4.37840455	1.27262594	-1.48603518
O	-1.58648910	6.50446848	-0.00000000
O	0.00000000	1.58516032	-6.50932154
O	3.50990426	-4.98382724	-0.00000000
O	0.00000000	-3.51099710	-4.99125017
O	1.48218324	-4.38799212	-1.27434060
O	-1.48218324	-4.38799212	-1.27434060
O	0.00000000	-1.58516032	-6.50932154
O	-1.58436044	-1.58614612	1.58990880
O	1.48218324	-4.38799212	1.27434060
O	-1.48218324	-4.38799212	1.27434060
O	0.00000000	-3.51099710	4.99125017
O	-1.27379172	1.48376869	4.39193141
O	0.00000000	1.58516032	6.50932154
O	-1.27379172	-1.48376869	4.39193141
O	0.00000000	-1.58516032	6.50932154
O	-4.98846014	0.00000000	3.51145706
O	-4.37840455	-1.27262594	1.48603517
O	-4.37840455	-1.27262594	-1.48603518
O	-3.50990426	-4.98382724	-0.00000000
O	-6.49724603	0.00000000	-1.57905017
O	-6.49724603	0.00000000	1.57905017
O	1.58648910	-6.50446848	-0.00000000
O	-1.58648910	-6.50446848	-0.00000000

$[Pt_{12}O_8(SO_4)_{12}]^{4-}$ (*D*2h)
Level: BP86/TZP/ZORA-scalar/COSMO-water

80

Pt	2.84593804	1.29096605	-0.00000000
Pt	0.00000000	2.84550087	-1.29095354
Pt	1.29089409	0.00000000	-2.84542517
Pt	1.29089409	0.00000000	2.84542517
Pt	0.00000000	2.84550087	1.29095354
Pt	-1.29089409	0.00000000	-2.84542517
Pt	-2.84593804	1.29096605	0.00000000
Pt	2.84593804	-1.29096605	-0.00000000
Pt	0.00000000	-2.84550087	-1.29095354
Pt	0.00000000	-2.84550087	1.29095354
Pt	-1.29089409	0.00000000	2.84542517
Pt	-2.84593804	-1.29096605	0.00000000
S	4.54182383	0.00000000	-2.29355262
S	4.54182383	0.00000000	2.29355262
S	2.29325694	4.54166237	-0.00000000
S	-2.29325694	4.54166237	0.00000000
S	0.00000000	2.29335879	-4.54158625
S	0.00000000	-2.29335879	-4.54158625
S	0.00000000	2.29335879	4.54158625
S	0.00000000	-2.29335879	4.54158625
S	-4.54182383	0.00000000	-2.29355262
S	-4.54182383	0.00000000	2.29355262
S	2.29325694	-4.54166237	0.00000000
S	-2.29325694	-4.54166237	0.00000000

O	1.52828954	1.52816933	-1.52805830
O	1.52828954	1.52816933	1.52805830
O	4.37950063	1.25349599	-1.39114940
O	4.37950063	1.25349599	1.39114940
O	3.34897760	3.45190493	0.00000000
O	-1.52828954	1.52816933	-1.52805830
O	-1.39147761	4.37862640	-1.25366363
O	1.39147761	4.37862640	-1.25366363
O	0.00000000	3.34876429	-3.45163972
O	1.52828954	-1.52816933	-1.52805830
O	3.45160300	0.00000000	-3.34845509
O	1.25349657	-1.39129778	-4.37871707
O	1.25349657	1.39129778	-4.37871707
O	1.52828954	-1.52816933	1.52805830
O	1.25349657	1.39129778	4.37871707
O	1.25349657	-1.39129778	4.37871707
O	3.45160300	0.00000000	3.34845509
O	-1.52828954	1.52816933	1.52805830
O	0.00000000	3.34876429	3.45163972
O	-1.39147761	4.37862640	1.25366363
O	1.39147761	4.37862640	1.25366363
O	4.37950063	-1.25349599	-1.39114940
O	5.86468671	0.00000000	-2.89343691
O	4.37950063	-1.25349599	1.39114940
O	5.86468671	0.00000000	2.89343691
O	2.89272546	5.86515829	0.00000000
O	-1.52828954	-1.52816933	-1.52805830
O	-1.25349657	-1.39129778	-4.37871707
O	-1.25349657	1.39129778	-4.37871707
O	-3.45160300	0.00000000	-3.34845509
O	-3.34897760	3.45190493	0.00000000
O	-4.37950063	1.25349599	1.39114940
O	-4.37950063	1.25349599	-1.39114940
O	-2.89272546	5.86515829	0.00000000
O	0.00000000	2.89292665	-5.86471980
O	3.34897760	-3.45190493	0.00000000
O	0.00000000	-3.34876429	-3.45163972
O	1.39147761	-4.37862640	-1.25366363
O	-1.39147761	-4.37862640	-1.25366363
O	0.00000000	-2.89292665	-5.86471980
O	-1.52828954	-1.52816933	1.52805830
O	1.39147761	-4.37862640	1.25366363
O	-1.39147761	-4.37862640	1.25366363
O	0.00000000	-3.34876429	3.45163972
O	-1.25349657	1.39129778	4.37871707
O	0.00000000	2.89292665	5.86471980
O	-1.25349657	-1.39129778	4.37871707
O	0.00000000	-2.89292665	5.86471980
O	-3.45160300	0.00000000	3.34845509
O	-4.37950063	-1.25349599	1.39114940
O	-4.37950063	-1.25349599	-1.39114940
O	-3.34897760	-3.45190493	0.00000000
O	-5.86468671	0.00000000	-2.89343691
O	-5.86468671	0.00000000	2.89343691
O	2.89272546	-5.86515829	0.00000000
O	-2.89272546	-5.86515829	0.00000000

$[Pt_{12}O_8(SO_4)_{12}]^{10-}$ (D_{2h})
Level: BP86/TZP/ZORA-scalar/COSMO-water

80

Pt	2.85452858	1.31349885	0.00000000
Pt	0.00000000	2.85399509	-1.31353452
Pt	1.31372498	0.00000000	-2.85475867
Pt	1.31372498	0.00000000	2.85475867
Pt	0.00000000	2.85399509	1.31353452
Pt	-1.31372498	0.00000000	-2.85475867
Pt	-2.85452858	1.31349885	0.00000000
Pt	2.85452858	-1.31349885	0.00000000
Pt	0.00000000	-2.85399509	-1.31353452
Pt	0.00000000	-2.85399509	1.31353452
Pt	-1.31372498	0.00000000	2.85475867
Pt	-2.85452858	-1.31349885	0.00000000
S	4.64967769	0.00000000	-2.31764704
S	4.64967769	0.00000000	2.31764704
S	2.31758565	4.64820990	0.00000000
S	-2.31758565	4.64820990	0.00000000
S	0.00000000	2.31782809	-4.64849412
S	0.00000000	-2.31782809	-4.64849412
S	0.00000000	2.31782809	4.64849412
S	0.00000000	-2.31782809	4.64849412
S	-4.64967769	0.00000000	-2.31764704
S	-4.64967769	0.00000000	2.31764704
S	2.31758565	-4.64820990	0.00000000
S	-2.31758565	-4.64820990	0.00000000
O	1.51231922	1.51201573	-1.51233090
O	1.51231922	1.51201573	1.51233090
O	4.45329603	1.25571312	-1.43978508
O	4.45329603	1.25571312	1.43978508
O	3.43523869	3.63902350	0.00000000
O	-1.51231922	1.51201573	-1.51233090
O	-1.44007999	4.45120429	-1.25578411
O	1.44007999	4.45120429	-1.25578411
O	0.00000000	3.43516289	-3.63889902
O	1.51231922	-1.51201573	-1.51233090
O	3.63933155	0.00000000	-3.43420664
O	1.25582610	-1.44040667	-4.45178538
O	1.25582610	1.44040667	-4.45178538
O	1.51231922	-1.51201573	1.51233090
O	1.25582610	1.44040667	4.45178538
O	1.25582610	-1.44040667	4.45178538
O	3.63933155	0.00000000	3.43420664
O	-1.51231922	1.51201573	1.51233090
O	0.00000000	3.43516289	3.63889902
O	-1.44007999	4.45120429	1.25578411
O	1.44007999	4.45120429	1.25578411
O	4.45329603	-1.25571312	-1.43978508
O	6.03495624	0.00000000	-2.83571073
O	4.45329603	-1.25571312	1.43978508
O	6.03495624	0.00000000	2.83571073
O	2.83412153	6.03381191	0.00000000
O	-1.51231922	-1.51201573	-1.51233090
O	-1.25582610	-1.44040667	-4.45178538
O	-1.25582610	1.44040667	-4.45178538
O	-3.63933155	0.00000000	-3.43420664
O	-3.43523869	3.63902350	0.00000000
O	-4.45329603	1.25571312	1.43978508
O	-4.45329603	1.25571312	-1.43978508
O	-2.83412153	6.03381191	0.00000000
O	0.00000000	2.83489671	-6.03379532
O	3.43523869	-3.63902350	0.00000000
O	0.00000000	-3.43516289	-3.63889902
O	1.44007999	-4.45120429	-1.25578411

O	-1.44007999	-4.45120429	-1.25578411
O	0.00000000	-2.83489671	-6.03379532
O	-1.51231922	-1.51201573	1.51233090
O	1.44007999	-4.45120429	1.25578411
O	-1.44007999	-4.45120429	1.25578411
O	0.00000000	-3.43516289	3.63889902
O	-1.25582610	1.44040667	4.45178538
O	0.00000000	2.83489671	6.03379532
O	-1.25582610	-1.44040667	4.45178538
O	0.00000000	-2.83489671	6.03379532
O	-3.63933155	0.00000000	3.43420664
O	-4.45329603	-1.25571312	1.43978508
O	-4.45329603	-1.25571312	-1.43978508
O	-3.43523869	-3.63902350	0.00000000
O	-6.03495624	0.00000000	-2.83571073
O	-6.03495624	0.00000000	2.83571073
O	2.83412153	-6.03381191	0.00000000
O	-2.83412153	-6.03381191	0.00000000

$[Pt_{12}O_8(SO_4)_{12}]^{16-}$ (D_{2h})
Level: BP86/TZP/ZORA-scalar/COSMO-water

80

Pt	2.80447998	1.38060921	-0.00000000
Pt	0.00000000	2.80593285	-1.37949478
Pt	1.38115116	-0.00000000	-2.80385467
Pt	1.38115116	-0.00000000	2.80385467
Pt	0.00000000	2.80593285	1.37949478
Pt	-1.38115116	-0.00000000	-2.80385467
Pt	-2.80447998	1.38060921	-0.00000000
Pt	2.80447998	-1.38060921	-0.00000000
Pt	0.00000000	-2.80593285	-1.37949478
Pt	0.00000000	-2.80593285	1.37949478
Pt	-1.38115116	-0.00000000	2.80385467
Pt	-2.80447998	-1.38060921	-0.00000000
S	5.06469764	-0.00000000	-2.01382929
S	5.06469764	-0.00000000	2.01382928
S	2.01481850	5.06926994	-0.00000000
S	-2.01481850	5.06926994	-0.00000000
S	0.00000000	2.01554721	-5.06469854
S	0.00000000	-2.01554721	-5.06469854
S	0.00000000	2.01554721	5.06469853
S	0.00000000	-2.01554721	5.06469853
S	-5.06469764	-0.00000000	-2.01382929
S	-5.06469764	-0.00000000	2.01382928
S	2.01481850	-5.06926994	-0.00000000
S	-2.01481850	-5.06926994	-0.00000000
O	1.58769076	1.58943067	-1.58813007
O	1.58769076	1.58943067	1.58813007
O	4.37983392	1.27458785	-1.48696836
O	4.37983392	1.27458785	1.48696836
O	3.51342653	4.98534837	-0.00000000
O	-1.58769076	1.58943067	-1.58813007
O	-1.48355547	4.38908639	-1.27543306
O	1.48355547	4.38908639	-1.27543306
O	0.00000000	3.51406275	-4.97809907
O	1.58769076	-1.58943067	-1.58813007
O	4.99069954	-0.00000000	-3.51296692
O	1.27526435	-1.48377070	-4.38455959
O	1.27526435	1.48377070	-4.38455959
O	1.58769076	-1.58943067	1.58813007

O	1.27526435	1.48377070	4.38455958
O	1.27526435	-1.48377070	4.38455958
O	4.99069954	-0.00000000	3.51296691
O	-1.58769076	1.58943067	1.58813007
O	0.00000000	3.51406275	4.97809907
O	-1.48355547	4.38908639	1.27543305
O	1.48355547	4.38908639	1.27543305
O	4.37983392	-1.27458785	-1.48696836
O	6.49952977	-0.00000000	-1.57919657
O	4.37983392	-1.27458785	1.48696836
O	6.49952977	-0.00000000	1.57919657
O	1.58955253	6.50695052	-0.00000000
O	-1.58769076	-1.58943067	-1.58813007
O	-1.27526435	-1.48377070	-4.38455959
O	-1.27526435	1.48377070	-4.38455959
O	-4.99069954	-0.00000000	-3.51296692
O	-3.51342653	4.98534837	-0.00000000
O	-4.37983392	1.27458785	1.48696836
O	-4.37983392	1.27458785	-1.48696836
O	-1.58955253	6.50695052	-0.00000000
O	0.00000000	1.59157491	-6.50250900
O	3.51342653	-4.98534837	-0.00000000
O	0.00000000	-3.51406275	-4.97809907
O	1.48355547	-4.38908639	-1.27543306
O	-1.48355547	-4.38908639	-1.27543306
O	0.00000000	-1.59157491	-6.50250900
O	-1.58769076	-1.58943067	1.58813007
O	1.48355547	-4.38908639	1.27543305
O	-1.48355547	-4.38908639	1.27543305
O	0.00000000	-3.51406275	4.97809907
O	-1.27526435	1.48377070	4.38455958
O	0.00000000	1.59157491	6.50250900
O	-1.27526435	-1.48377070	4.38455958
O	0.00000000	-1.59157491	6.50250900
O	-4.99069954	-0.00000000	3.51296691
O	-4.37983392	-1.27458785	1.48696836
O	-4.37983392	-1.27458785	-1.48696836
O	-3.51342653	-4.98534837	-0.00000000
O	-6.49952977	-0.00000000	-1.57919657
O	-6.49952977	-0.00000000	1.57919657
O	1.58955253	-6.50695052	-0.00000000
O	-1.58955253	-6.50695052	-0.00000000

$[K_8Pt_{12}O_8(SO_4)_{12}]^{4+}$ (D_{2h})
Level: BP86/TZP/ZORA-scalar/COSMO-water

88

K	-3.54625102	3.55241712	3.46150631
K	-3.39854194	3.71654395	-3.73697300
K	3.54191638	3.54154888	3.52911593
K	3.55706381	3.54110095	-3.52233011
K	-3.50278515	-3.53779341	3.50338855
K	3.54624154	-3.54573780	3.52980126
K	3.54560212	-3.54969347	-3.53024086
K	-3.53896487	-3.46808400	-3.51671205
Pt	2.85160559	1.29192313	0.00132264
Pt	-0.00229122	2.84414184	-1.29011701
Pt	1.29586432	-0.00272267	-2.84645005
Pt	1.29745703	-0.00315885	2.85422339
Pt	0.00478980	2.84758383	1.29807770
Pt	-1.29335887	-0.00253217	-2.84728177

Pt	-2.84790711	1.29028179	0.00666261
Pt	2.85216209	-1.29576166	0.00339634
Pt	0.00247755	-2.85102363	-1.29186695
Pt	0.00262969	-2.85256275	1.29659591
Pt	-1.29073019	-0.00302182	2.85402324
Pt	-2.84828912	-1.29687774	0.00284921
S	4.55816272	-0.00418500	-2.29138376
S	4.55744988	0.00010704	2.29580285
S	2.29367844	4.55251411	-0.00348568
S	-2.29485822	4.54918333	0.00872097
S	0.00409812	2.28911061	-4.55260162
S	0.00138891	-2.29612725	-4.55362752
S	0.00321967	2.29125164	4.55796328
S	0.00501757	-2.29801235	4.55800262
S	-4.55169259	0.00464072	-2.28767400
S	-4.55281122	-0.00768745	2.30112913
S	2.29735284	-4.55675100	0.00143076
S	-2.29213396	-4.55787937	0.00038859
O	1.53973747	1.53694593	-1.53593778
O	1.54218931	1.53476990	1.54238131
O	4.36825152	1.26072096	-1.40399878
O	4.37161141	1.26131611	1.40226177
O	3.35217745	3.45327181	-0.01193559
O	-1.53738965	1.53179186	-1.53240721
O	-1.41774155	4.35333736	-1.26189430
O	1.39483925	4.36816161	-1.26034800
O	-0.00647193	3.34547947	-3.45074208
O	1.54125805	-1.54076905	-1.53613087
O	3.45865349	-0.00852181	-3.34849188
O	1.26346712	-1.40391643	-4.36694531
O	1.26812142	1.40318150	-4.36375777
O	1.54172920	-1.54165357	1.54232027
O	1.26425425	1.39668629	4.37599019
O	1.26736687	-1.40684630	4.37283683
O	3.45728318	0.00224481	3.35219571
O	-1.53607932	1.53743788	1.54506120
O	0.01009937	3.34819650	3.45748864
O	-1.38741830	4.37565786	1.26252884
O	1.41183266	4.36251131	1.26473890
O	4.37280621	-1.26444569	-1.39698073
O	5.87179652	-0.00357615	-2.89220431
O	4.36969814	-1.26407673	1.40680930
O	5.86979381	0.00048661	2.89913665
O	2.89646246	5.86511314	-0.00587349
O	-1.53823083	-1.54174934	-1.53691354
O	-1.26167694	-1.40488578	-4.36833446
O	-1.25735496	1.39590887	-4.37186827
O	-3.45115757	-0.00649834	-3.34495867
O	-3.35028912	3.44898538	0.03871248
O	-4.36467073	1.25663423	1.41300803
O	-4.36608711	1.26074857	-1.39352450
O	-2.89871425	5.86138869	0.00618783
O	0.00352607	2.89902605	-5.86223910
O	3.35349735	-3.45652089	0.00492362
O	0.00159683	-3.35072423	-3.45169121
O	1.40640923	-4.36918433	-1.26135668
O	-1.39983284	-4.37049795	-1.26053903
O	0.00374420	-2.90087598	-5.86501522
O	-1.53620350	-1.54180980	1.54252575
O	1.40404597	-4.37335722	1.26299600
O	-1.40178632	-4.36980247	1.26402945
O	0.00366452	-3.35416677	3.45660487

O	-1.26040248	1.40279456	4.36915749
O	0.00229713	2.89610030	5.86961036
O	-1.25745562	-1.40661575	4.37314870
O	0.00572448	-2.90473846	5.86885071
O	-3.45214424	-0.01152471	3.35711455
O	-4.36691343	-1.26767399	1.40593203
O	-4.36849007	-1.26256568	-1.39752344
O	-3.35038173	-3.45915042	0.00136250
O	-5.86246820	0.00259005	-2.89451269
O	-5.86562966	-0.00876327	2.90361157
O	2.89904358	-5.86988741	-0.00057007
O	-2.89183088	-5.87188832	0.00068283

$[K_8Pt_{12}O_8(SO_4)_{12}]^{2-}$ (D_{2h})
Level: BP86/TZP/ZORA-scalar/COSMO-water

88

K	-3.19113424	3.21712610	3.17985881
K	-3.19113424	3.21712610	-3.17985881
K	3.19113424	3.21712610	3.17985881
K	3.19113424	3.21712610	-3.17985881
K	-3.19113424	-3.21712610	3.17985881
K	-3.19113424	-3.21712610	-3.17985881
K	3.19113424	-3.21712610	3.17985881
K	3.19113424	-3.21712610	-3.17985881
Pt	2.86775096	1.31607703	0.00000000
Pt	0.00000000	2.86913342	-1.31602495
Pt	1.31606938	0.00000000	-2.86830319
Pt	1.31606938	0.00000000	2.86830319
Pt	0.00000000	2.86913342	1.31602495
Pt	-1.31606938	0.00000000	-2.86830319
Pt	-2.86775096	1.31607703	0.00000000
Pt	2.86775096	-1.31607703	0.00000000
Pt	0.00000000	-2.86913342	-1.31602495
Pt	0.00000000	-2.86913342	1.31602495
Pt	-1.31606938	0.00000000	2.86830319
Pt	-2.86775096	-1.31607703	0.00000000
S	4.67125556	0.00000000	-2.31399227
S	4.67125556	0.00000000	2.31399227
S	2.31237727	4.67641567	0.00000000
S	-2.31237727	4.67641567	0.00000000
S	0.00000000	2.31331509	-4.67326731
S	0.00000000	-2.31331509	-4.67326731
S	0.00000000	2.31331509	4.67326731
S	0.00000000	-2.31331509	4.67326731
S	-4.67125556	0.00000000	-2.31399227
S	-4.67125556	0.00000000	2.31399227
S	2.31237727	-4.67641567	0.00000000
S	-2.31237727	-4.67641567	0.00000000
O	1.52914247	1.52978546	-1.52950218
O	1.52914247	1.52978546	1.52950218
O	4.44608731	1.26324501	-1.44108113
O	4.44608731	1.26324501	1.44108113
O	3.42856007	3.65737914	0.00000000
O	-1.52914247	1.52978546	-1.52950218
O	-1.43945265	4.45028537	-1.26257135
O	1.43945265	4.45028537	-1.26257135
O	0.00000000	3.42983615	-3.65436246
O	1.52914247	-1.52978546	-1.52950218
O	3.65294555	0.00000000	-3.42993555
O	1.26281173	-1.44081832	-4.44775455

O	1.26281173	1.44081832	-4.44775455
O	1.52914247	-1.52978546	1.52950218
O	1.26281173	1.44081832	4.44775455
O	1.26281173	-1.44081832	4.44775455
O	3.65294555	0.00000000	3.42993555
O	-1.52914247	1.52978546	1.52950218
O	0.00000000	3.42983615	3.65436246
O	-1.43945265	4.45028537	1.26257135
O	1.43945265	4.45028537	1.26257135
O	4.44608731	-1.26324501	-1.44108113
O	6.04903929	0.00000000	-2.81145795
O	4.44608731	-1.26324501	1.44108113
O	6.04903929	0.00000000	2.81145795
O	2.81093042	6.05345105	0.00000000
O	-1.52914247	-1.52978546	-1.52950218
O	-1.26281173	-1.44081832	-4.44775455
O	-1.26281173	1.44081832	-4.44775455
O	-3.65294555	0.00000000	-3.42993555
O	-3.42856007	3.65737914	0.00000000
O	-4.44608731	1.26324501	1.44108113
O	-4.44608731	1.26324501	-1.44108113
O	-2.81093042	6.05345105	0.00000000
O	0.00000000	2.81309629	-6.04999803
O	3.42856007	-3.65737914	0.00000000
O	0.00000000	-3.42983615	-3.65436246
O	1.43945265	-4.45028537	-1.26257135
O	-1.43945265	-4.45028537	-1.26257135
O	0.00000000	-2.81309629	-6.04999803
O	-1.52914247	-1.52978546	1.52950218
O	1.43945265	-4.45028537	1.26257135
O	-1.43945265	-4.45028537	1.26257135
O	0.00000000	-3.42983615	3.65436246
O	-1.26281173	1.44081832	4.44775455
O	0.00000000	2.81309629	6.04999803
O	-1.26281173	-1.44081832	4.44775455
O	0.00000000	-2.81309629	6.04999803
O	-3.65294555	0.00000000	3.42993555
O	-4.44608731	-1.26324501	1.44108113
O	-4.44608731	-1.26324501	-1.44108113
O	-3.42856007	-3.65737914	0.00000000
O	-6.04903929	0.00000000	-2.81145795
O	-6.04903929	0.00000000	2.81145795
O	2.81093042	-6.05345105	0.00000000
O	-2.81093042	-6.05345105	0.00000000

$[K_8Pt_{12}O_8(SO_4)_{12}]^{8-}$ (D_{2h})
Level: BP86/TZP/ZORA-scalar/COSMO-water

88

K	-3.10091149	3.10743490	-3.09690293
K	-3.10091149	3.10743490	3.09690293
K	-3.10091149	-3.10743490	3.09690293
K	-3.10091149	-3.10743490	-3.09690293
K	3.10091149	3.10743490	3.09690293
K	3.10091149	3.10743490	-3.09690293
K	3.10091149	-3.10743490	3.09690293
K	3.10091149	-3.10743490	-3.09690293
Pt	2.81985257	1.37301828	0.00000000
Pt	0.00000000	2.82546913	-1.37267598
Pt	1.37192692	0.00000000	-2.82248169
Pt	1.37192692	0.00000000	2.82248169

Pt	0.00000000	2.82546913	1.37267598
Pt	-1.37192692	0.00000000	-2.82248169
Pt	-2.81985257	1.37301828	0.00000000
Pt	2.81985257	-1.37301828	0.00000000
Pt	0.00000000	-2.82546913	-1.37267598
Pt	0.00000000	-2.82546913	1.37267598
Pt	-1.37192692	0.00000000	2.82248169
Pt	-2.81985257	-1.37301828	0.00000000
S	5.04130650	0.00000000	-2.05168029
S	5.04130650	0.00000000	2.05168029
S	2.05689213	5.04335882	0.00000000
S	-2.05689213	5.04335882	0.00000000
S	0.00000000	2.05171140	-5.04507105
S	0.00000000	-2.05171140	-5.04507105
S	0.00000000	2.05171140	5.04507105
S	0.00000000	-2.05171140	5.04507105
S	-5.04130650	0.00000000	-2.05168029
S	-5.04130650	0.00000000	2.05168029
S	2.05689213	-5.04335882	0.00000000
S	-2.05689213	-5.04335882	0.00000000
O	1.57671545	1.57936949	-1.57835025
O	1.57671545	1.57936949	1.57835025
O	4.37238928	1.28129281	-1.48247982
O	4.37238928	1.28129281	1.48247982
O	3.53602608	4.85320475	0.00000000
O	-1.57671545	1.57936949	-1.57835025
O	-1.47392394	4.38719079	-1.28158024
O	1.47392394	4.38719079	-1.28158024
O	0.00000000	3.53384858	-4.88351984
O	1.57671545	-1.57936949	-1.57835025
O	4.87627623	0.00000000	-3.53383028
O	1.28143580	-1.48483594	-4.37427846
O	1.28143580	1.48483594	-4.37427846
O	1.57671545	-1.57936949	1.57835025
O	1.28143580	1.48483594	4.37427846
O	1.28143580	-1.48483594	4.37427846
O	4.87627623	0.00000000	3.53383028
O	-1.57671545	1.57936949	1.57835025
O	0.00000000	3.53384858	4.88351984
O	-1.47392394	4.38719079	1.28158024
O	1.47392394	4.38719079	1.28158024
O	4.37238928	-1.28129281	-1.48247982
O	6.47722787	0.00000000	-1.65773547
O	4.37238928	-1.28129281	1.48247982
O	6.47722787	0.00000000	1.65773547
O	1.68749528	6.48622740	0.00000000
O	-1.57671545	-1.57936949	-1.57835025
O	-1.28143580	-1.48483594	-4.37427846
O	-1.28143580	1.48483594	-4.37427846
O	-4.87627623	0.00000000	-3.53383028
O	-3.53602608	4.85320475	0.00000000
O	-4.37238928	1.28129281	1.48247982
O	-4.37238928	1.28129281	-1.48247982
O	-1.68749528	6.48622740	0.00000000
O	0.00000000	1.65290273	-6.47981430
O	3.53602608	-4.85320475	0.00000000
O	0.00000000	-3.53384858	-4.88351984
O	1.47392394	-4.38719079	-1.28158024
O	-1.47392394	-4.38719079	-1.28158024
O	0.00000000	-1.65290273	-6.47981430
O	-1.57671545	-1.57936949	1.57835025
O	1.47392394	-4.38719079	1.28158024

O	-1.47392394	-4.38719079	1.28158024
O	0.00000000	-3.53384858	4.88351984
O	-1.28143580	1.48483594	4.37427846
O	0.00000000	1.65290273	6.47981430
O	-1.28143580	-1.48483594	4.37427846
O	0.00000000	-1.65290273	6.47981430
O	-4.87627623	0.00000000	3.53383028
O	-4.37238928	-1.28129281	1.48247982
O	-4.37238928	-1.28129281	-1.48247982
O	-3.53602608	-4.85320475	0.00000000
O	-6.47722787	0.00000000	-1.65773547
O	-6.47722787	0.00000000	1.65773547
O	1.68749528	-6.48622740	0.00000000
O	-1.68749528	-6.48622740	0.00000000

[Be⊂$Pt_{12}O_8(SO_4)_{12}$]$^{2-}$ (D_{2h})
Level: BP86/TZP/ZORA-scalar/COSMO-water

81

Be	0.00000000	0.00000000	0.00000000
Pt	2.88011983	1.29318009	0.00000000
Pt	0.00000000	2.87925319	-1.29311469
Pt	1.29309114	0.00000000	-2.87940051
Pt	1.29309114	0.00000000	2.87940051
Pt	0.00000000	2.87925319	1.29311469
Pt	-1.29309114	0.00000000	-2.87940051
Pt	-2.88011983	1.29318009	0.00000000
Pt	2.88011983	-1.29318009	0.00000000
Pt	0.00000000	-2.87925319	-1.29311469
Pt	0.00000000	-2.87925319	1.29311469
Pt	-1.29309114	0.00000000	2.87940051
Pt	-2.88011983	-1.29318009	0.00000000
S	4.55886011	0.00000000	-2.26589816
S	4.55886011	0.00000000	2.26589816
S	2.26548704	4.55865941	0.00000000
S	-2.26548704	4.55865941	0.00000000
S	0.00000000	2.26561619	-4.55878085
S	0.00000000	-2.26561619	-4.55878085
S	0.00000000	2.26561619	4.55878085
S	0.00000000	-2.26561619	4.55878085
S	-4.55886011	0.00000000	-2.26589816
S	-4.55886011	0.00000000	2.26589816
S	2.26548704	-4.55865941	0.00000000
S	-2.26548704	-4.55865941	0.00000000
O	1.25168374	1.25147058	-1.25139617
O	1.25168374	1.25147058	1.25139617
O	4.41490927	1.26299625	-1.35843205
O	4.41490927	1.26299625	1.35843205
O	3.29493267	3.44649546	0.00000000
O	-1.25168374	1.25147058	-1.25139617
O	-1.35893050	4.41329491	-1.26331711
O	1.35893050	4.41329491	-1.26331711
O	0.00000000	3.29419410	-3.44591575
O	1.25168374	-1.25147058	-1.25139617
O	3.44563465	0.00000000	-3.29380502
O	1.26326365	-1.35882725	-4.41362175
O	1.26326365	1.35882725	-4.41362175
O	1.25168374	-1.25147058	1.25139617
O	1.26326365	1.35882725	4.41362175
O	1.26326365	-1.35882725	4.41362175
O	3.44563465	0.00000000	3.29380502

O	-1.25168374	1.25147058	1.25139617
O	0.00000000	3.29419410	3.44591575
O	-1.35893050	4.41329491	1.26331711
O	1.35893050	4.41329491	1.26331711
O	4.41490927	-1.26299625	-1.35843205
O	5.86803044	0.00000000	-2.88260571
O	4.41490927	-1.26299625	1.35843205
O	5.86803044	0.00000000	2.88260571
O	2.88043177	5.86861171	0.00000000
O	-1.25168374	-1.25147058	-1.25139617
O	-1.26326365	-1.35882725	-4.41362175
O	-1.26326365	1.35882725	-4.41362175
O	-3.44563465	0.00000000	-3.29380502
O	-3.29493267	3.44649546	0.00000000
O	-4.41490927	1.26299625	1.35843205
O	-4.41490927	1.26299625	-1.35843205
O	-2.88043177	5.86861171	0.00000000
O	0.00000000	2.88107604	-5.86834515
O	3.29493267	-3.44649546	0.00000000
O	0.00000000	-3.29419410	-3.44591575
O	1.35893050	-4.41329491	-1.26331711
O	-1.35893050	-4.41329491	-1.26331711
O	0.00000000	-2.88107604	-5.86834515
O	-1.25168374	-1.25147058	1.25139617
O	1.35893050	-4.41329491	1.26331711
O	-1.35893050	-4.41329491	1.26331711
O	0.00000000	-3.29419410	3.44591575
O	-1.26326365	1.35882725	4.41362175
O	0.00000000	2.88107604	5.86834515
O	-1.26326365	-1.35882725	4.41362175
O	0.00000000	-2.88107604	5.86834515
O	-3.44563465	0.00000000	3.29380502
O	-4.41490927	-1.26299625	1.35843205
O	-4.41490927	-1.26299625	-1.35843205
O	-3.29493267	-3.44649546	0.00000000
O	-5.86803044	0.00000000	-2.88260571
O	-5.86803044	0.00000000	2.88260571
O	2.88043177	-5.86861171	0.00000000
O	-2.88043177	-5.86861171	0.00000000

$[Na{\subset}Pt_{12}O_8(SO_4)_{12}]^{3-}$ (D_{2h})
Level: BP86/TZP/ZORA-scalar/COSMO-water
81

Na	0.00000000	0.00000000	0.00000000
Pt	-2.88490344	-1.29867107	0.00000000
Pt	-1.29852092	0.00000000	-2.88380704
Pt	-2.88490344	1.29867107	0.00000000
Pt	2.88490344	-1.29867107	0.00000000
Pt	0.00000000	-2.88446212	-1.29860796
Pt	0.00000000	-2.88446212	1.29860796
Pt	-1.29852092	0.00000000	2.88380704
Pt	0.00000000	2.88446212	-1.29860796
Pt	1.29852092	0.00000000	-2.88380704
Pt	1.29852092	0.00000000	2.88380704
Pt	0.00000000	2.88446212	1.29860796
Pt	2.88490344	1.29867107	0.00000000
S	0.00000000	-2.28981720	4.55402030
S	-4.55406786	0.00000000	-2.28983855
S	-4.55406786	0.00000000	2.28983855
S	2.28965738	-4.55407353	0.00000000
S	-2.28965738	-4.55407353	0.00000000
S	-2.28965738	4.55407353	0.00000000

S	0.00000000	2.28981720	-4.55402030
S	0.00000000	-2.28981720	-4.55402030
S	0.00000000	2.28981720	4.55402030
S	4.55406786	0.00000000	-2.28983855
S	4.55406786	0.00000000	2.28983855
S	2.28965738	4.55407353	0.00000000
O	1.44248778	1.44245193	-1.44206423
O	1.44248778	1.44245193	1.44206423
O	4.40424523	1.25750791	-1.38260022
O	4.40424523	1.25750791	1.38260022
O	3.33544746	3.45646402	0.00000000
O	-1.44248778	1.44245193	-1.44206423
O	-1.38303821	4.40321098	-1.25789025
O	1.38303821	4.40321098	-1.25789025
O	0.00000000	3.33556952	-3.45644833
O	1.44248778	-1.44245193	-1.44206423
O	3.45575139	0.00000000	-3.33489055
O	1.25768555	-1.38326955	-4.40241182
O	1.25768555	1.38326955	-4.40241182
O	1.44248778	-1.44245193	1.44206423
O	1.25768555	1.38326955	4.40241182
O	1.25768555	-1.38326955	4.40241182
O	3.45575139	0.00000000	3.33489055
O	-1.44248778	1.44245193	1.44206423
O	0.00000000	3.33556952	3.45644833
O	-1.38303821	4.40321098	1.25789025
O	1.38303821	4.40321098	1.25789025
O	4.40424523	-1.25750791	-1.38260022
O	5.87106681	0.00000000	-2.89633562
O	4.40424523	-1.25750791	1.38260022
O	5.87106681	0.00000000	2.89633562
O	2.89455018	5.87179327	0.00000000
O	-1.44248778	-1.44245193	-1.44206423
O	-1.25768555	-1.38326955	-4.40241182
O	-1.25768555	1.38326955	-4.40241182
O	-3.45575139	0.00000000	-3.33489055
O	-3.33544746	3.45646402	0.00000000
O	-4.40424523	1.25750791	1.38260022
O	-4.40424523	1.25750791	-1.38260022
O	-2.89455018	5.87179327	0.00000000
O	0.00000000	2.89416019	-5.87191707
O	3.33544746	-3.45646402	0.00000000
O	0.00000000	-3.33556952	-3.45644833
O	1.38303821	-4.40321098	-1.25789025
O	-1.38303821	-4.40321098	-1.25789025
O	0.00000000	-2.89416019	-5.87191707
O	-1.44248778	-1.44245193	1.44206423
O	1.38303821	-4.40321098	1.25789025
O	-1.38303821	-4.40321098	1.25789025
O	0.00000000	-3.33556952	3.45644833
O	-1.25768555	1.38326955	4.40241182
O	0.00000000	2.89416019	5.87191707
O	-1.25768555	-1.38326955	4.40241182
O	0.00000000	-2.89416019	5.87191707
O	-3.45575139	0.00000000	3.33489055
O	-4.40424523	-1.25750791	1.38260022
O	-4.40424523	-1.25750791	-1.38260022
O	-3.33544746	-3.45646402	0.00000000
O	-5.87106681	0.00000000	-2.89633562
O	-5.87106681	0.00000000	2.89633562
O	2.89455018	-5.87179327	0.00000000
O	-2.89455018	-5.87179327	0.00000000

[Cs⊂$Pt_{12}O_8(SO_4)_{12}$]$^{3-}$ (D_{2h})
Level: BP86/TZP/ZORA-scalar/COSMO-water

81

Cs	0.00000000	0.00000000	0.00000000
Pt	2.90844559	1.32228343	0.00000000
Pt	0.00000000	2.90794459	-1.32233883
Pt	1.32214369	0.00000000	-2.90768262
Pt	1.32214369	0.00000000	2.90768262
Pt	0.00000000	2.90794459	1.32233883
Pt	-1.32214369	0.00000000	-2.90768262
Pt	-2.90844559	1.32228343	0.00000000
Pt	2.90844559	-1.32228343	0.00000000
Pt	0.00000000	-2.90794459	-1.32233883
Pt	0.00000000	-2.90794459	1.32233883
Pt	-1.32214369	0.00000000	2.90768262
Pt	-2.90844559	-1.32228343	0.00000000
S	4.56722407	0.00000000	-2.30790688
S	4.56722407	0.00000000	2.30790688
S	2.30753993	4.56715903	0.00000000
S	-2.30753993	4.56715903	0.00000000
S	0.00000000	2.30763525	-4.56726171
S	0.00000000	-2.30763525	-4.56726171
S	0.00000000	2.30763525	4.56726171
S	0.00000000	-2.30763525	4.56726171
S	-4.56722407	0.00000000	-2.30790688
S	-4.56722407	0.00000000	2.30790688
S	2.30753993	-4.56715903	0.00000000
S	-2.30753993	-4.56715903	0.00000000
O	1.60869636	1.60861242	-1.60843486
O	1.60869636	1.60861242	1.60843486
O	4.41589963	1.25588353	-1.40138116
O	4.41589963	1.25588353	1.40138116
O	3.36424190	3.47692891	0.00000000
O	-1.60869636	1.60861242	-1.60843486
O	-1.40188255	4.41477777	-1.25636007
O	1.40188255	4.41477777	-1.25636007
O	0.00000000	3.36419996	-3.47685556
O	1.60869636	-1.60861242	-1.60843486
O	3.47599038	0.00000000	-3.36335704
O	1.25615258	-1.40192940	-4.41438092
O	1.25615258	1.40192940	-4.41438092
O	1.60869636	-1.60861242	1.60843486
O	1.25615258	1.40192940	4.41438092
O	1.25615258	-1.40192940	4.41438092
O	3.47599038	0.00000000	3.36335704
O	-1.60869636	1.60861242	1.60843486
O	0.00000000	3.36419996	3.47685556
O	-1.40188255	4.41477777	1.25636007
O	1.40188255	4.41477777	1.25636007
O	4.41589963	-1.25588353	-1.40138116
O	5.88509863	0.00000000	-2.91276957
O	4.41589963	-1.25588353	1.40138116
O	5.88509863	0.00000000	2.91276957
O	2.91045817	5.88561915	0.00000000
O	-1.60869636	-1.60861242	-1.60843486
O	-1.25615258	-1.40192940	-4.41438092
O	-1.25615258	1.40192940	-4.41438092
O	-3.47599038	0.00000000	-3.36335704
O	-3.36424190	3.47692891	0.00000000

O	-4.41589963	1.25588353	1.40138116
O	-4.41589963	1.25588353	-1.40138116
O	-2.91045817	5.88561915	0.00000000
O	0.00000000	2.91080710	-5.88583816
O	3.36424190	-3.47692891	0.00000000
O	0.00000000	-3.36419996	-3.47685556
O	1.40188255	-4.41477777	-1.25636007
O	-1.40188255	-4.41477777	-1.25636007
O	0.00000000	-2.91080710	-5.88583816
O	-1.60869636	-1.60861242	1.60843486
O	1.40188255	-4.41477777	1.25636007
O	-1.40188255	-4.41477777	1.25636007
O	0.00000000	-3.36419996	3.47685556
O	-1.25615258	1.40192940	4.41438092
O	0.00000000	2.91080710	5.88583816
O	-1.25615258	-1.40192940	4.41438092
O	0.00000000	-2.91080710	5.88583816
O	-3.47599038	0.00000000	3.36335704
O	-4.41589963	-1.25588353	1.40138116
O	-4.41589963	-1.25588353	-1.40138116
O	-3.36424190	-3.47692891	0.00000000
O	-5.88509863	0.00000000	-2.91276957
O	-5.88509863	0.00000000	2.91276957
O	2.91045817	-5.88561915	0.00000000
O	-2.91045817	-5.88561915	0.00000000

$[Pt_{12}O_8(PO_4)_{12}]^{16-}$ (D_{2h})
Level: BP86/TZP/ZORA-scalar/COSMO-water

80

Pt	2.86795083	1.30470872	0.00000000
Pt	0.00000000	2.86724035	-1.30424852
Pt	1.30461004	0.00000000	-2.86561241
Pt	1.30461004	0.00000000	2.86561241
Pt	0.00000000	2.86724035	1.30424852
Pt	-1.30461004	0.00000000	-2.86561241
Pt	-2.86795083	1.30470872	0.00000000
Pt	2.86795083	-1.30470872	0.00000000
Pt	0.00000000	-2.86724035	-1.30424852
Pt	0.00000000	-2.86724035	1.30424852
Pt	-1.30461004	0.00000000	2.86561241
Pt	-2.86795083	-1.30470872	0.00000000
P	4.56376602	0.00000000	-2.30347395
P	4.56376602	0.00000000	2.30347395
P	2.30291761	4.56311678	0.00000000
P	-2.30291761	4.56311678	0.00000000
P	0.00000000	2.30431755	-4.56263397
P	0.00000000	-2.30431755	-4.56263397
P	0.00000000	2.30431755	4.56263397
P	0.00000000	-2.30431755	4.56263397
P	-4.56376602	0.00000000	-2.30347395
P	-4.56376602	0.00000000	2.30347395
P	2.30291761	-4.56311678	0.00000000
P	-2.30291761	-4.56311678	0.00000000
O	1.53159465	1.53143284	-1.53062278
O	1.53159465	1.53143284	1.53062278
O	4.40409661	1.29122146	-1.38679473
O	4.40409661	1.29122146	1.38679473
O	3.41239670	3.45892692	0.00000000
O	-1.53159465	1.53143284	-1.53062278
O	-1.38498763	4.40409175	-1.29068115

O	1.38498763	4.40409175	-1.29068115
O	0.00000000	3.41623462	-3.46002699
O	1.53159465	-1.53143284	-1.53062278
O	3.46062795	0.00000000	-3.41456238
O	1.29064379	-1.38724052	-4.40114136
O	1.29064379	1.38724052	-4.40114136
O	1.53159465	-1.53143284	1.53062278
O	1.29064379	1.38724052	4.40114136
O	1.29064379	-1.38724052	4.40114136
O	3.46062795	0.00000000	3.41456238
O	-1.53159465	1.53143284	1.53062278
O	0.00000000	3.41623462	3.46002699
O	-1.38498763	4.40409175	1.29068115
O	1.38498763	4.40409175	1.29068115
O	4.40409661	-1.29122146	-1.38679473
O	5.96096585	0.00000000	-2.93011575
O	4.40409661	-1.29122146	1.38679473
O	5.96096585	0.00000000	2.93011575
O	2.92932533	5.96004005	0.00000000
O	-1.53159465	-1.53143284	-1.53062278
O	-1.29064379	-1.38724052	-4.40114136
O	-1.29064379	1.38724052	-4.40114136
O	-3.46062795	0.00000000	-3.41456238
O	-3.41239670	3.45892692	0.00000000
O	-4.40409661	1.29122146	1.38679473
O	-4.40409661	1.29122146	-1.38679473
O	-2.92932533	5.96004005	0.00000000
O	0.00000000	2.92873708	-5.96055470
O	3.41239670	-3.45892692	0.00000000
O	0.00000000	-3.41623462	-3.46002699
O	1.38498763	-4.40409175	-1.29068115
O	-1.38498763	-4.40409175	-1.29068115
O	0.00000000	-2.92873708	-5.96055470
O	-1.53159465	-1.53143284	1.53062278
O	1.38498763	-4.40409175	1.29068115
O	-1.38498763	-4.40409175	1.29068115
O	0.00000000	-3.41623462	3.46002699
O	-1.29064379	1.38724052	4.40114136
O	0.00000000	2.92873708	5.96055470
O	-1.29064379	-1.38724052	4.40114136
O	0.00000000	-2.92873708	5.96055470
O	-3.46062795	0.00000000	3.41456238
O	-4.40409661	-1.29122146	1.38679473
O	-4.40409661	-1.29122146	-1.38679473
O	-3.41239670	-3.45892692	0.00000000
O	-5.96096585	0.00000000	-2.93011575
O	-5.96096585	0.00000000	2.93011575
O	2.92932533	-5.96004005	0.00000000
O	-2.92932533	-5.96004005	0.00000000

$[Pt_{12}O_8(SO_4)_8]^{8-}$ (D_{2h})
Level: BP86/TZP/ZORA-scalar/COSMO-water
60

Pt	0.00000000	-2.36052115	2.36052115
Pt	2.36052115	0.00000000	2.36052115
Pt	2.36052115	-2.36052115	-0.00000000
Pt	-2.36052115	0.00000000	2.36052115
Pt	-2.36052115	-2.36052115	-0.00000000
Pt	0.00000000	2.36052115	2.36052115
Pt	2.36052115	2.36052115	-0.00000000
Pt	0.00000000	-2.36052115	-2.36052115

Pt	2.36052115	0.00000000	-2.36052115
Pt	0.00000000	2.36052115	-2.36052115
Pt	-2.36052115	2.36052115	-0.00000000
Pt	-2.36052115	0.00000000	-2.36052115
S	-3.05718462	-3.05718462	3.05718462
S	3.05718462	-3.05718462	3.05718462
S	3.05718462	3.05718462	3.05718462
S	3.05718462	-3.05718462	-3.05718462
S	-3.05718462	3.05718462	3.05718462
S	-3.05718462	-3.05718462	-3.05718462
S	3.05718462	3.05718462	-3.05718462
S	-3.05718462	3.05718462	-3.05718462
O	1.32230584	-1.32230584	1.32230584
O	-1.32230584	-1.32230584	1.32230584
O	-1.57941865	-3.37481749	3.37481749
O	1.57941865	-3.37481749	3.37481749
O	3.90833779	-3.90833779	3.90833779
O	1.32230584	1.32230584	1.32230584
O	3.37481749	-1.57941865	3.37481749
O	3.37481749	1.57941865	3.37481749
O	1.32230584	-1.32230584	-1.32230584
O	3.37481749	-3.37481749	1.57941865
O	3.37481749	-3.37481749	-1.57941865
O	1.32230584	1.32230584	-1.32230584
O	-1.32230584	1.32230584	1.32230584
O	-1.32230584	-1.32230584	-1.32230584
O	-1.32230584	1.32230584	-1.32230584
O	-3.37481749	1.57941865	3.37481749
O	-3.37481749	-1.57941865	3.37481749
O	-3.37481749	-3.37481749	-1.57941865
O	-3.37481749	-3.37481749	1.57941865
O	-1.57941865	3.37481749	3.37481749
O	1.57941865	3.37481749	3.37481749
O	3.37481749	3.37481749	1.57941865
O	3.37481749	3.37481749	-1.57941865
O	3.90833779	3.90833779	3.90833779
O	-1.57941865	-3.37481749	-3.37481749
O	1.57941865	-3.37481749	-3.37481749
O	3.37481749	-1.57941865	-3.37481749
O	3.37481749	1.57941865	-3.37481749
O	3.90833779	-3.90833779	-3.90833779
O	-1.57941865	3.37481749	-3.37481749
O	1.57941865	3.37481749	-3.37481749
O	-3.37481749	3.37481749	-1.57941865
O	-3.37481749	3.37481749	1.57941865
O	-3.37481749	1.57941865	-3.37481749
O	-3.37481749	-1.57941865	-3.37481749
O	-3.90833779	3.90833779	3.90833779
O	-3.90833779	-3.90833779	-3.90833779
O	3.90833779	3.90833779	-3.90833779
O	-3.90833779	3.90833779	-3.90833779
O	-3.90833779	-3.90833779	3.90833779

$[Pt_{12}O_8(PO_4)_8]^{16-}$ (D_{2h})
Level: BP86/TZP/ZORA-scalar/COSMO-water

60

Pt	-0.00000000	-2.37919310	2.37919310
Pt	2.37919310	0.00000000	2.37919310
Pt	2.37919310	-2.37919310	0.00000000

Pt	-2.37919310	0.00000000	2.37919310
Pt	-2.37919310	-2.37919310	0.00000000
Pt	-0.00000000	2.37919310	2.37919310
Pt	2.37919310	2.37919310	0.00000000
Pt	-0.00000000	-2.37919310	-2.37919310
Pt	2.37919310	0.00000000	-2.37919310
Pt	-0.00000000	2.37919310	-2.37919310
Pt	-2.37919310	2.37919310	0.00000000
Pt	-2.37919310	0.00000000	-2.37919310
P	-3.07506480	-3.07506480	3.07506480
P	3.07506480	-3.07506480	3.07506480
P	3.07506480	3.07506480	3.07506480
P	3.07506480	-3.07506480	-3.07506480
P	-3.07506480	3.07506480	3.07506480
P	-3.07506480	-3.07506480	-3.07506480
P	3.07506480	3.07506480	-3.07506480
P	-3.07506480	3.07506480	-3.07506480
O	1.33456474	-1.33456474	1.33456474
O	-1.33456474	-1.33456474	1.33456474
O	-1.55201385	-3.40777710	3.40777710
O	1.55201385	-3.40777710	3.40777710
O	3.96571295	-3.96571295	3.96571295
O	1.33456474	1.33456474	1.33456474
O	3.40777710	-1.55201385	3.40777710
O	3.40777710	1.55201385	3.40777710
O	1.33456474	-1.33456474	-1.33456474
O	3.40777710	-3.40777710	1.55201385
O	3.40777710	-3.40777710	-1.55201385
O	1.33456474	1.33456474	-1.33456474
O	-1.33456474	1.33456474	1.33456474
O	-1.33456474	-1.33456474	-1.33456474
O	-1.33456474	1.33456474	-1.33456474
O	-3.40777710	1.55201385	3.40777710
O	-3.40777710	-1.55201385	3.40777710
O	-3.40777710	-3.40777710	-1.55201385
O	-3.40777710	-3.40777710	1.55201385
O	-1.55201385	3.40777710	3.40777710
O	1.55201385	3.40777710	3.40777710
O	3.40777710	3.40777710	1.55201385
O	3.40777710	3.40777710	-1.55201385
O	3.96571295	3.96571295	3.96571295
O	-1.55201385	-3.40777710	-3.40777710
O	1.55201385	-3.40777710	-3.40777710
O	3.40777710	-1.55201385	-3.40777710
O	3.40777710	1.55201385	-3.40777710
O	3.96571295	-3.96571295	-3.96571295
O	-1.55201385	3.40777710	-3.40777710
O	1.55201385	3.40777710	-3.40777710
O	-3.40777710	3.40777710	-1.55201385
O	-3.40777710	3.40777710	1.55201385
O	-3.40777710	1.55201385	-3.40777710
O	-3.40777710	-1.55201385	-3.40777710
O	-3.96571295	3.96571295	3.96571295
O	-3.96571295	-3.96571295	-3.96571295
O	3.96571295	3.96571295	-3.96571295
O	-3.96571295	3.96571295	-3.96571295
O	-3.96571295	-3.96571295	3.96571295

Cartesian coordinates (in Angstroms) of the Au/POM/Au junction with $\{[Pt_{12}O_8(SO_4)_{12}]\}$.

368
xyz

Au	0.000000e+00	0.000000e+00	1.453735e+00
Au	2.945068e+00	0.000000e+00	1.453735e+00
Au	5.890136e+00	0.000000e+00	1.453735e+00
Au	8.835204e+00	0.000000e+00	1.453735e+00
Au	1.178027e+01	0.000000e+00	1.453735e+00
Au	1.472534e+01	0.000000e+00	1.453735e+00
Au	-1.472534e+00	2.550504e+00	1.453735e+00
Au	1.472534e+00	2.550504e+00	1.453735e+00
Au	4.417602e+00	2.550504e+00	1.453735e+00
Au	7.362670e+00	2.550504e+00	1.453735e+00
Au	1.030774e+01	2.550504e+00	1.453735e+00
Au	1.325281e+01	2.550504e+00	1.453735e+00
Au	-2.945068e+00	5.101007e+00	1.453735e+00
Au	-9.000000e-12	5.101007e+00	1.453735e+00
Au	2.945068e+00	5.101007e+00	1.453735e+00
Au	5.890136e+00	5.101007e+00	1.453735e+00
Au	8.835204e+00	5.101007e+00	1.453735e+00
Au	1.178027e+01	5.101007e+00	1.453735e+00
Au	-4.417602e+00	7.651511e+00	1.453735e+00
Au	-1.472534e+00	7.651511e+00	1.453735e+00
Au	1.472534e+00	7.651511e+00	1.453735e+00
Au	4.417602e+00	7.651511e+00	1.453735e+00
Au	7.362670e+00	7.651511e+00	1.453735e+00
Au	1.030774e+01	7.651511e+00	1.453735e+00
Au	-5.890136e+00	1.020201e+01	1.453735e+00
Au	-2.945068e+00	1.020201e+01	1.453735e+00
Au	0.000000e+00	1.020201e+01	1.453735e+00
Au	2.945068e+00	1.020201e+01	1.453735e+00
Au	5.890136e+00	1.020201e+01	1.453735e+00
Au	8.835204e+00	1.020201e+01	1.453735e+00
Au	-7.362670e+00	1.275252e+01	1.453735e+00
Au	-4.417602e+00	1.275252e+01	1.453735e+00
Au	-1.472534e+00	1.275252e+01	1.453735e+00
Au	1.472534e+00	1.275252e+01	1.453735e+00
Au	4.417602e+00	1.275252e+01	1.453735e+00
Au	7.362670e+00	1.275252e+01	1.453735e+00
Au	1.472534e+00	8.501679e-01	3.858373e+00
Au	4.417602e+00	8.501679e-01	3.858373e+00
Au	7.362670e+00	8.501679e-01	3.858373e+00
Au	1.030774e+01	8.501679e-01	3.858373e+00
Au	1.325281e+01	8.501679e-01	3.858373e+00
Au	1.619787e+01	8.501679e-01	3.858373e+00
Au	9.000000e-12	3.400672e+00	3.858373e+00
Au	2.945068e+00	3.400672e+00	3.858373e+00
Au	5.890136e+00	3.400672e+00	3.858373e+00
Au	8.835204e+00	3.400672e+00	3.858373e+00
Au	1.178027e+01	3.400672e+00	3.858373e+00
Au	1.472534e+01	3.400672e+00	3.858373e+00
Au	-1.472534e+00	5.951175e+00	3.858373e+00
Au	1.472534e+00	5.951175e+00	3.858373e+00
Au	4.417602e+00	5.951175e+00	3.858373e+00
Au	7.362670e+00	5.951175e+00	3.858373e+00
Au	1.030774e+01	5.951175e+00	3.858373e+00
Au	1.325281e+01	5.951175e+00	3.858373e+00
Au	-2.945068e+00	8.501679e+00	3.858373e+00
Au	0.000000e+00	8.501679e+00	3.858373e+00
Au	2.945068e+00	8.501679e+00	3.858373e+00

Au	5.890136e+00	8.501679e+00	3.858373e+00
Au	8.835204e+00	8.501679e+00	3.858373e+00
Au	1.178027e+01	8.501679e+00	3.858373e+00
Au	-4.417602e+00	1.105218e+01	3.858373e+00
Au	-1.472534e+00	1.105218e+01	3.858373e+00
Au	1.472534e+00	1.105218e+01	3.858373e+00
Au	4.417602e+00	1.105218e+01	3.858373e+00
Au	7.362670e+00	1.105218e+01	3.858373e+00
Au	1.030774e+01	1.105218e+01	3.858373e+00
Au	-5.890136e+00	1.360269e+01	3.858373e+00
Au	-2.945068e+00	1.360269e+01	3.858373e+00
Au	-9.000000e-12	1.360269e+01	3.858373e+00
Au	2.945068e+00	1.360269e+01	3.858373e+00
Au	5.890136e+00	1.360269e+01	3.858373e+00
Au	8.835204e+00	1.360269e+01	3.858373e+00
Au	-0.000000e+00	1.700336e+00	6.263011e+00
Au	2.945068e+00	1.700336e+00	6.263011e+00
Au	5.890136e+00	1.700336e+00	6.263011e+00
Au	8.835204e+00	1.700336e+00	6.263011e+00
Au	1.178027e+01	1.700336e+00	6.263011e+00
Au	1.472534e+01	1.700336e+00	6.263011e+00
Au	-1.472534e+00	4.250839e+00	6.263011e+00
Au	1.472534e+00	4.250839e+00	6.263011e+00
Au	4.417602e+00	4.250839e+00	6.263011e+00
Au	7.362670e+00	4.250839e+00	6.263011e+00
Au	1.030774e+01	4.250839e+00	6.263011e+00
Au	1.325281e+01	4.250839e+00	6.263011e+00
Au	-2.945068e+00	6.801343e+00	6.263011e+00
Au	-0.000000e+00	6.801343e+00	6.263011e+00
Au	2.945068e+00	6.801343e+00	6.263011e+00
Au	5.890136e+00	6.801343e+00	6.263011e+00
Au	8.835204e+00	6.801343e+00	6.263011e+00
Au	1.178027e+01	6.801343e+00	6.263011e+00
Au	-4.417602e+00	9.351847e+00	6.263011e+00
Au	-1.472534e+00	9.351847e+00	6.263011e+00
Au	1.472534e+00	9.351847e+00	6.263011e+00
Au	4.417602e+00	9.351847e+00	6.263011e+00
Au	7.362670e+00	9.351847e+00	6.263011e+00
Au	1.030774e+01	9.351847e+00	6.263011e+00
Au	-5.890136e+00	1.190235e+01	6.263011e+00
Au	-2.945068e+00	1.190235e+01	6.263011e+00
Au	9.000000e-12	1.190235e+01	6.263011e+00
Au	2.945068e+00	1.190235e+01	6.263011e+00
Au	5.890136e+00	1.190235e+01	6.263011e+00
Au	8.835204e+00	1.190235e+01	6.263011e+00
Au	-7.362670e+00	1.445285e+01	6.263011e+00
Au	-4.417602e+00	1.445285e+01	6.263011e+00
Au	-1.472534e+00	1.445285e+01	6.263011e+00
Au	1.472534e+00	1.445285e+01	6.263011e+00
Au	4.417602e+00	1.445285e+01	6.263011e+00
Au	7.362670e+00	1.445285e+01	6.263011e+00
Au	6.121079e+00	1.012457e+01	8.455440e+00
Au	6.087970e+00	5.176935e+00	8.499066e+00
Au	4.425656e+00	1.282994e+01	8.580958e+00
Au	4.416328e+00	2.448940e+00	8.590352e+00
Au	7.397747e+00	1.276245e+01	8.608713e+00
Au	7.398479e+00	2.550537e+00	8.616270e+00
Au	8.964722e+00	1.021093e+01	8.618837e+00
Au	2.805207e+00	1.016293e+01	8.628199e+00
Au	8.945679e+00	5.104115e+00	8.628992e+00
Au	1.033119e+01	2.553748e+00	8.641638e+00
Au	1.178772e+01	2.841440e-02	8.647998e+00

Au	1.473764e+01	2.548370e-02	8.649134e+00
Au	8.852116e+00	8.069351e-03	8.652917e+00
Au	1.469692e+00	1.277035e+01	8.655886e+00
Au	7.497606e+00	7.651537e+00	8.659199e+00
Au	2.824276e+00	5.140370e+00	8.659574e+00
Au	1.326548e+01	2.556163e+00	8.659841e+00
Au	1.038151e+01	7.656737e+00	8.663820e+00
Au	0.000000e+00	0.000000e+00	8.667649e+00
Au	1.181942e+01	5.096237e+00	8.668295e+00
Au	5.916710e+00	-1.929691e-02	8.669100e+00
Au	-7.340568e+00	1.276110e+01	8.676154e+00
Au	-1.471112e+00	2.550078e+00	8.676247e+00
Au	2.935156e+00	-2.484591e-02	8.678560e+00
Au	1.467732e+00	2.533566e+00	8.685124e+00
Au	-5.840878e+00	1.021141e+01	8.686929e+00
Au	-1.469071e+00	1.276455e+01	8.687938e+00
Au	-4.406693e+00	1.275952e+01	8.690708e+00
Au	-4.404109e+00	7.655092e+00	8.692243e+00
Au	-2.946043e+00	5.097259e+00	8.694095e+00
Au	4.438153e+00	7.658825e+00	8.702800e+00
Au	-2.947362e+00	1.021259e+01	8.703795e+00
Au	-5.925249e-02	1.020569e+01	8.709082e+00
Au	-4.553389e-02	5.094813e+00	8.726877e+00
Au	-1.498108e+00	7.650672e+00	8.773572e+00
Au	1.415625e+00	7.649273e+00	8.805029e+00
O	4.639267e+00	1.080479e+01	1.010018e+01
O	4.637920e+00	4.400723e+00	1.015240e+01
O	3.366248e+00	8.977292e+00	1.123674e+01
O	3.340785e+00	6.214440e+00	1.127014e+01
S	4.643615e+00	9.890598e+00	1.136359e+01
O	5.913918e+00	8.985356e+00	1.136392e+01
O	5.891970e+00	6.223336e+00	1.137873e+01
S	4.624203e+00	5.313538e+00	1.140051e+01
O	1.277380e+00	7.621901e+00	1.210478e+01
O	-1.170060e+00	7.634868e+00	1.240417e+01
O	4.588726e+00	1.093479e+01	1.246249e+01
O	4.586267e+00	4.279844e+00	1.251121e+01
O	7.922625e+00	7.595384e+00	1.254602e+01
Pt	3.276284e+00	7.615297e+00	1.283210e+01
Pt	5.859020e+00	7.603684e+00	1.297843e+01
O	1.033511e+01	7.590859e+00	1.306677e+01
S	1.079952e-01	7.633725e+00	1.315333e+01
S	8.994301e+00	7.594602e+00	1.368602e+01
O	2.188577e-01	8.920977e+00	1.406699e+01
O	2.069853e-01	6.357457e+00	1.408375e+01
O	3.001275e+00	9.102009e+00	1.414262e+01
O	2.989886e+00	6.157000e+00	1.417204e+01
O	6.035940e+00	6.126377e+00	1.432137e+01
O	6.047325e+00	9.072060e+00	1.432361e+01
O	3.216043e+00	1.190537e+01	1.438195e+01
O	3.122603e+00	3.370797e+00	1.441423e+01
Pt	4.522237e+00	1.037351e+01	1.448057e+01
O	5.903017e+00	1.184227e+01	1.450057e+01
Pt	4.483550e+00	4.854867e+00	1.451480e+01
O	5.803754e+00	3.330660e+00	1.456068e+01
O	8.822879e+00	8.880640e+00	1.459298e+01
O	8.813445e+00	6.312254e+00	1.459714e+01
O	1.104185e+00	1.096462e+01	1.549228e+01
O	1.605147e+00	1.340942e+01	1.555646e+01
Pt	1.597745e+00	8.896818e+00	1.556347e+01
Pt	1.580296e+00	6.377646e+00	1.558489e+01
S	2.191992e+00	1.205604e+01	1.558797e+01

O	1.057051e+00	4.315879e+00	1.560046e+01
O	1.531733e+00	1.865073e+00	1.561773e+01
S	2.134804e+00	3.211119e+00	1.564884e+01
S	6.732716e+00	1.202642e+01	1.584266e+01
O	7.335054e+00	1.337298e+01	1.586433e+01
S	6.680715e+00	3.185635e+00	1.587694e+01
Pt	7.266701e+00	6.345047e+00	1.589975e+01
Pt	7.282333e+00	8.862457e+00	1.590051e+01
O	7.770847e+00	4.278341e+00	1.590684e+01
O	7.269238e+00	1.833233e+00	1.592275e+01
O	7.807156e+00	1.092305e+01	1.595018e+01
O	6.061015e-02	8.943135e+00	1.687491e+01
O	2.383974e-02	6.374473e+00	1.687785e+01
O	3.021304e+00	1.187382e+01	1.693067e+01
Pt	4.378836e+00	1.038309e+01	1.695902e+01
O	3.010437e+00	3.360962e+00	1.696595e+01
Pt	4.350356e+00	4.865784e+00	1.699570e+01
O	5.704638e+00	1.189961e+01	1.704769e+01
O	5.698150e+00	3.371197e+00	1.711178e+01
O	2.834853e+00	9.102790e+00	1.713655e+01
O	2.809946e+00	6.151638e+00	1.716235e+01
O	5.880953e+00	9.094495e+00	1.731789e+01
O	5.860055e+00	6.152561e+00	1.731981e+01
O	8.662185e+00	8.876428e+00	1.739837e+01
O	8.634210e+00	6.312524e+00	1.740840e+01
S	-1.351419e-01	7.662196e+00	1.778506e+01
S	8.752333e+00	7.597154e+00	1.832590e+01
O	-1.475788e+00	7.683703e+00	1.840466e+01
Pt	3.000365e+00	7.636923e+00	1.848884e+01
Pt	5.583686e+00	7.626178e+00	1.864465e+01
O	9.365065e-01	7.645138e+00	1.892449e+01
O	4.318637e+00	1.095098e+01	1.896043e+01
O	4.265316e+00	4.299478e+00	1.901712e+01
O	1.002979e+01	7.584605e+00	1.907673e+01
O	7.582076e+00	7.617187e+00	1.937449e+01
O	2.943283e+00	9.040184e+00	2.006956e+01
S	4.226865e+00	9.925288e+00	2.007508e+01
O	2.950261e+00	6.264029e+00	2.009907e+01
S	4.216668e+00	5.354280e+00	2.010528e+01
O	5.503386e+00	6.251515e+00	2.022575e+01
O	5.490394e+00	8.996842e+00	2.023436e+01
O	4.204724e+00	1.085691e+01	2.131017e+01
O	4.205097e+00	4.466044e+00	2.138358e+01
Au	9.828019e-01	7.714594e+00	2.273096e+01
Au	-1.930931e+00	7.715993e+00	2.276242e+01
Au	-4.783568e-01	5.160134e+00	2.280911e+01
Au	-4.920754e-01	1.027101e+01	2.282691e+01
Au	-3.380185e+00	1.027791e+01	2.283219e+01
Au	4.005330e+00	7.724146e+00	2.283319e+01
Au	-3.378866e+00	5.162581e+00	2.284189e+01
Au	-4.836932e+00	7.720413e+00	2.284375e+01
Au	-4.839516e+00	1.282484e+01	2.284528e+01
Au	-1.901894e+00	1.282987e+01	2.284805e+01
Au	-6.273701e+00	1.027673e+01	2.284906e+01
Au	1.034909e+00	2.598887e+00	2.285086e+01
Au	2.502334e+00	4.047530e-02	2.285743e+01
Au	-1.903935e+00	2.615400e+00	2.285974e+01
Au	-7.773391e+00	1.282642e+01	2.285983e+01
Au	5.483887e+00	4.602430e-02	2.286689e+01
Au	1.138660e+01	5.161559e+00	2.286769e+01
Au	-4.328229e-01	6.532121e-02	2.286834e+01
Au	9.948688e+00	7.722058e+00	2.287217e+01

Au	1.283266e+01	2.621484e+00	2.287615e+01
Au	2.391453e+00	5.205691e+00	2.287641e+01
Au	7.064783e+00	7.716858e+00	2.287679e+01
Au	1.036869e+00	1.283567e+01	2.288010e+01
Au	8.419293e+00	7.339056e-02	2.288307e+01
Au	1.430482e+01	9.080491e-02	2.288685e+01
Au	1.135490e+01	9.373561e-02	2.288799e+01
Au	9.898365e+00	2.619069e+00	2.289435e+01
Au	8.512856e+00	5.169437e+00	2.290700e+01
Au	2.372384e+00	1.022825e+01	2.290779e+01
Au	8.531899e+00	1.027625e+01	2.291715e+01
Au	6.965656e+00	2.615858e+00	2.291972e+01
Au	6.964924e+00	1.282777e+01	2.292728e+01
Au	3.983505e+00	2.514261e+00	2.294564e+01
Au	3.992833e+00	1.289526e+01	2.295503e+01
Au	5.655147e+00	5.242256e+00	2.303692e+01
Au	5.688256e+00	1.018989e+01	2.308055e+01
Au	-4.328229e-01	1.765657e+00	2.527298e+01
Au	2.512245e+00	1.765657e+00	2.527298e+01
Au	5.457313e+00	1.765657e+00	2.527298e+01
Au	8.402381e+00	1.765657e+00	2.527298e+01
Au	1.134745e+01	1.765657e+00	2.527298e+01
Au	1.429252e+01	1.765657e+00	2.527298e+01
Au	-1.905357e+00	4.316161e+00	2.527298e+01
Au	1.039711e+00	4.316161e+00	2.527298e+01
Au	3.984779e+00	4.316161e+00	2.527298e+01
Au	6.929847e+00	4.316161e+00	2.527298e+01
Au	9.874915e+00	4.316161e+00	2.527298e+01
Au	1.281998e+01	4.316161e+00	2.527298e+01
Au	-3.377891e+00	6.866664e+00	2.527298e+01
Au	-4.328229e-01	6.866664e+00	2.527298e+01
Au	2.512245e+00	6.866664e+00	2.527298e+01
Au	5.457313e+00	6.866664e+00	2.527298e+01
Au	8.402381e+00	6.866664e+00	2.527298e+01
Au	1.134745e+01	6.866664e+00	2.527298e+01
Au	-4.850425e+00	9.417168e+00	2.527298e+01
Au	-1.905357e+00	9.417168e+00	2.527298e+01
Au	1.039711e+00	9.417168e+00	2.527298e+01
Au	3.984779e+00	9.417168e+00	2.527298e+01
Au	6.929847e+00	9.417168e+00	2.527298e+01
Au	9.874915e+00	9.417168e+00	2.527298e+01
Au	-6.322959e+00	1.196767e+01	2.527298e+01
Au	-3.377891e+00	1.196767e+01	2.527298e+01
Au	-4.328229e-01	1.196767e+01	2.527298e+01
Au	2.512245e+00	1.196767e+01	2.527298e+01
Au	5.457313e+00	1.196767e+01	2.527298e+01
Au	8.402381e+00	1.196767e+01	2.527298e+01
Au	-7.795493e+00	1.451818e+01	2.527298e+01
Au	-4.850425e+00	1.451818e+01	2.527298e+01
Au	-1.905357e+00	1.451818e+01	2.527298e+01
Au	1.039711e+00	1.451818e+01	2.527298e+01
Au	3.984779e+00	1.451818e+01	2.527298e+01
Au	6.929847e+00	1.451818e+01	2.527298e+01
Au	1.039711e+00	9.154891e-01	2.767762e+01
Au	3.984779e+00	9.154891e-01	2.767762e+01
Au	6.929847e+00	9.154891e-01	2.767762e+01
Au	9.874915e+00	9.154891e-01	2.767762e+01
Au	1.281998e+01	9.154891e-01	2.767762e+01
Au	1.576505e+01	9.154891e-01	2.767762e+01
Au	-4.328229e-01	3.465993e+00	2.767762e+01
Au	2.512245e+00	3.465993e+00	2.767762e+01
Au	5.457313e+00	3.465993e+00	2.767762e+01

Au	8.402381e+00	3.465993e+00	2.767762e+01
Au	1.134745e+01	3.465993e+00	2.767762e+01
Au	1.429252e+01	3.465993e+00	2.767762e+01
Au	-1.905357e+00	6.016496e+00	2.767762e+01
Au	1.039711e+00	6.016496e+00	2.767762e+01
Au	3.984779e+00	6.016496e+00	2.767762e+01
Au	6.929847e+00	6.016496e+00	2.767762e+01
Au	9.874915e+00	6.016496e+00	2.767762e+01
Au	1.281998e+01	6.016496e+00	2.767762e+01
Au	-3.377891e+00	8.567000e+00	2.767762e+01
Au	-4.328229e-01	8.567000e+00	2.767762e+01
Au	2.512245e+00	8.567000e+00	2.767762e+01
Au	5.457313e+00	8.567000e+00	2.767762e+01
Au	8.402381e+00	8.567000e+00	2.767762e+01
Au	1.134745e+01	8.567000e+00	2.767762e+01
Au	-4.850425e+00	1.111750e+01	2.767762e+01
Au	-1.905357e+00	1.111750e+01	2.767762e+01
Au	1.039711e+00	1.111750e+01	2.767762e+01
Au	3.984779e+00	1.111750e+01	2.767762e+01
Au	6.929847e+00	1.111750e+01	2.767762e+01
Au	9.874915e+00	1.111750e+01	2.767762e+01
Au	-6.322959e+00	1.366801e+01	2.767762e+01
Au	-3.377891e+00	1.366801e+01	2.767762e+01
Au	-4.328229e-01	1.366801e+01	2.767762e+01
Au	2.512245e+00	1.366801e+01	2.767762e+01
Au	5.457313e+00	1.366801e+01	2.767762e+01
Au	8.402381e+00	1.366801e+01	2.767762e+01
Au	-4.328229e-01	6.532121e-02	3.008225e+01
Au	2.512245e+00	6.532121e-02	3.008225e+01
Au	5.457313e+00	6.532121e-02	3.008225e+01
Au	8.402381e+00	6.532121e-02	3.008225e+01
Au	1.134745e+01	6.532121e-02	3.008225e+01
Au	1.429252e+01	6.532121e-02	3.008225e+01
Au	-1.905357e+00	2.615825e+00	3.008225e+01
Au	1.039711e+00	2.615825e+00	3.008225e+01
Au	3.984779e+00	2.615825e+00	3.008225e+01
Au	6.929847e+00	2.615825e+00	3.008225e+01
Au	9.874915e+00	2.615825e+00	3.008225e+01
Au	1.281998e+01	2.615825e+00	3.008225e+01
Au	-3.377891e+00	5.166329e+00	3.008225e+01
Au	-4.328229e-01	5.166329e+00	3.008225e+01
Au	2.512245e+00	5.166329e+00	3.008225e+01
Au	5.457313e+00	5.166329e+00	3.008225e+01
Au	8.402381e+00	5.166329e+00	3.008225e+01
Au	1.134745e+01	5.166329e+00	3.008225e+01
Au	-4.850425e+00	7.716832e+00	3.008225e+01
Au	-1.905357e+00	7.716832e+00	3.008225e+01
Au	1.039711e+00	7.716832e+00	3.008225e+01
Au	3.984779e+00	7.716832e+00	3.008225e+01
Au	6.929847e+00	7.716832e+00	3.008225e+01
Au	9.874915e+00	7.716832e+00	3.008225e+01
Au	-6.322959e+00	1.026734e+01	3.008225e+01
Au	-3.377891e+00	1.026734e+01	3.008225e+01
Au	-4.328229e-01	1.026734e+01	3.008225e+01
Au	2.512245e+00	1.026734e+01	3.008225e+01
Au	5.457313e+00	1.026734e+01	3.008225e+01
Au	8.402381e+00	1.026734e+01	3.008225e+01
Au	-7.795493e+00	1.281784e+01	3.008225e+01
Au	-4.850425e+00	1.281784e+01	3.008225e+01
Au	-1.905357e+00	1.281784e+01	3.008225e+01
Au	1.039711e+00	1.281784e+01	3.008225e+01
Au	3.984779e+00	1.281784e+01	3.008225e+01

Au 6.929847e+00 1.281784e+01 3.008225e+01

Cartesian coordinates (in Angstroms) of the Au/POM/Au junction with $\{K_4[Pt_{12}O_8(SO_4)_{12}]\}$.
372
xyz

Au 0.000000e+00 0.000000e+00 1.235393e+00
Au 2.945068e+00 0.000000e+00 1.235393e+00
Au 5.890136e+00 0.000000e+00 1.235393e+00
Au 8.835204e+00 0.000000e+00 1.235393e+00
Au 1.178027e+01 0.000000e+00 1.235393e+00
Au 1.472534e+01 0.000000e+00 1.235393e+00
Au -1.472534e+00 2.550504e+00 1.235393e+00
Au 1.472534e+00 2.550504e+00 1.235393e+00
Au 4.417602e+00 2.550504e+00 1.235393e+00
Au 7.362670e+00 2.550504e+00 1.235393e+00
Au 1.030774e+01 2.550504e+00 1.235393e+00
Au 1.325281e+01 2.550504e+00 1.235393e+00
Au -2.945068e+00 5.101007e+00 1.235393e+00
Au -9.000000e-12 5.101007e+00 1.235393e+00
Au 2.945068e+00 5.101007e+00 1.235393e+00
Au 5.890136e+00 5.101007e+00 1.235393e+00
Au 8.835204e+00 5.101007e+00 1.235393e+00
Au 1.178027e+01 5.101007e+00 1.235393e+00
Au -4.417602e+00 7.651511e+00 1.235393e+00
Au -1.472534e+00 7.651511e+00 1.235393e+00
Au 1.472534e+00 7.651511e+00 1.235393e+00
Au 4.417602e+00 7.651511e+00 1.235393e+00
Au 7.362670e+00 7.651511e+00 1.235393e+00
Au 1.030774e+01 7.651511e+00 1.235393e+00
Au -5.890136e+00 1.020201e+01 1.235393e+00
Au -2.945068e+00 1.020201e+01 1.235393e+00
Au 0.000000e+00 1.020201e+01 1.235393e+00
Au 2.945068e+00 1.020201e+01 1.235393e+00
Au 5.890136e+00 1.020201e+01 1.235393e+00
Au 8.835204e+00 1.020201e+01 1.235393e+00
Au -7.362670e+00 1.275252e+01 1.235393e+00
Au -4.417602e+00 1.275252e+01 1.235393e+00
Au -1.472534e+00 1.275252e+01 1.235393e+00
Au 1.472534e+00 1.275252e+01 1.235393e+00
Au 4.417602e+00 1.275252e+01 1.235393e+00
Au 7.362670e+00 1.275252e+01 1.235393e+00
Au 1.472534e+00 8.501679e-01 3.640031e+00
Au 4.417602e+00 8.501679e-01 3.640031e+00
Au 7.362670e+00 8.501679e-01 3.640031e+00
Au 1.030774e+01 8.501679e-01 3.640031e+00
Au 1.325281e+01 8.501679e-01 3.640031e+00
Au 1.619787e+01 8.501679e-01 3.640031e+00
Au 9.000000e-12 3.400672e+00 3.640031e+00
Au 2.945068e+00 3.400672e+00 3.640031e+00
Au 5.890136e+00 3.400672e+00 3.640031e+00
Au 8.835204e+00 3.400672e+00 3.640031e+00
Au 1.178027e+01 3.400672e+00 3.640031e+00
Au 1.472534e+01 3.400672e+00 3.640031e+00
Au -1.472534e+00 5.951175e+00 3.640031e+00
Au 1.472534e+00 5.951175e+00 3.640031e+00
Au 4.417602e+00 5.951175e+00 3.640031e+00
Au 7.362670e+00 5.951175e+00 3.640031e+00
Au 1.030774e+01 5.951175e+00 3.640031e+00
Au 1.325281e+01 5.951175e+00 3.640031e+00
Au -2.945068e+00 8.501679e+00 3.640031e+00
Au 0.000000e+00 8.501679e+00 3.640031e+00

Au	2.945068e+00	8.501679e+00	3.640031e+00
Au	5.890136e+00	8.501679e+00	3.640031e+00
Au	8.835204e+00	8.501679e+00	3.640031e+00
Au	1.178027e+01	8.501679e+00	3.640031e+00
Au	-4.417602e+00	1.105218e+01	3.640031e+00
Au	-1.472534e+00	1.105218e+01	3.640031e+00
Au	1.472534e+00	1.105218e+01	3.640031e+00
Au	4.417602e+00	1.105218e+01	3.640031e+00
Au	7.362670e+00	1.105218e+01	3.640031e+00
Au	1.030774e+01	1.105218e+01	3.640031e+00
Au	-5.890136e+00	1.360269e+01	3.640031e+00
Au	-2.945068e+00	1.360269e+01	3.640031e+00
Au	-9.000000e-12	1.360269e+01	3.640031e+00
Au	2.945068e+00	1.360269e+01	3.640031e+00
Au	5.890136e+00	1.360269e+01	3.640031e+00
Au	8.835204e+00	1.360269e+01	3.640031e+00
Au	-0.000000e+00	1.700336e+00	6.044669e+00
Au	2.945068e+00	1.700336e+00	6.044669e+00
Au	5.890136e+00	1.700336e+00	6.044669e+00
Au	8.835204e+00	1.700336e+00	6.044669e+00
Au	1.178027e+01	1.700336e+00	6.044669e+00
Au	1.472534e+01	1.700336e+00	6.044669e+00
Au	-1.472534e+00	4.250839e+00	6.044669e+00
Au	1.472534e+00	4.250839e+00	6.044669e+00
Au	4.417602e+00	4.250839e+00	6.044669e+00
Au	7.362670e+00	4.250839e+00	6.044669e+00
Au	1.030774e+01	4.250839e+00	6.044669e+00
Au	1.325281e+01	4.250839e+00	6.044669e+00
Au	-2.945068e+00	6.801343e+00	6.044669e+00
Au	-0.000000e+00	6.801343e+00	6.044669e+00
Au	2.945068e+00	6.801343e+00	6.044669e+00
Au	5.890136e+00	6.801343e+00	6.044669e+00
Au	8.835204e+00	6.801343e+00	6.044669e+00
Au	1.178027e+01	6.801343e+00	6.044669e+00
Au	-4.417602e+00	9.351847e+00	6.044669e+00
Au	-1.472534e+00	9.351847e+00	6.044669e+00
Au	1.472534e+00	9.351847e+00	6.044669e+00
Au	4.417602e+00	9.351847e+00	6.044669e+00
Au	7.362670e+00	9.351847e+00	6.044669e+00
Au	1.030774e+01	9.351847e+00	6.044669e+00
Au	-5.890136e+00	1.190235e+01	6.044669e+00
Au	-2.945068e+00	1.190235e+01	6.044669e+00
Au	9.000000e-12	1.190235e+01	6.044669e+00
Au	2.945068e+00	1.190235e+01	6.044669e+00
Au	5.890136e+00	1.190235e+01	6.044669e+00
Au	8.835204e+00	1.190235e+01	6.044669e+00
Au	-7.362670e+00	1.445285e+01	6.044669e+00
Au	-4.417602e+00	1.445285e+01	6.044669e+00
Au	-1.472534e+00	1.445285e+01	6.044669e+00
Au	1.472534e+00	1.445285e+01	6.044669e+00
Au	4.417602e+00	1.445285e+01	6.044669e+00
Au	7.362670e+00	1.445285e+01	6.044669e+00
Au	2.836520e+00	1.018065e+01	8.320513e+00
Au	2.844732e+00	5.132599e+00	8.336587e+00
Au	5.967940e+00	1.018995e+01	8.390944e+00
Au	4.430303e+00	1.277339e+01	8.401538e+00
Au	5.961665e+00	5.115286e+00	8.410952e+00
Au	4.430611e+00	2.521506e+00	8.417561e+00
Au	1.422052e+00	7.653030e+00	8.421778e+00
Au	1.459021e+00	2.545617e+00	8.422645e+00
Au	-5.311773e-02	5.101853e+00	8.425370e+00
Au	1.462698e+00	1.276821e+01	8.425744e+00

Au	4.419977e+00	7.653645e+00	8.426512e+00
Au	-1.477604e+00	2.546075e+00	8.429512e+00
Au	1.473371e+01	2.029591e-03	8.436668e+00
Au	1.178391e+01	2.013644e-03	8.437175e+00
Au	-4.555661e-02	1.020184e+01	8.439683e+00
Au	1.325714e+01	2.544424e+00	8.441326e+00
Au	-2.959046e+00	5.094366e+00	8.445783e+00
Au	0.000000e+00	0.000000e+00	8.449307e+00
Au	-1.496749e+00	7.652467e+00	8.451467e+00
Au	-1.472234e+00	1.275469e+01	8.453664e+00
Au	8.842063e+00	2.950144e-03	8.454060e+00
Au	1.031825e+01	2.544516e+00	8.457132e+00
Au	2.939954e+00	-1.287459e-03	8.457445e+00
Au	-2.955073e+00	1.020711e+01	8.461350e+00
Au	5.908174e+00	-4.697349e-03	8.462151e+00
Au	1.179252e+01	5.093728e+00	8.462613e+00
Au	-4.414814e+00	7.651992e+00	8.465127e+00
Au	7.380833e+00	1.275984e+01	8.466961e+00
Au	7.384078e+00	2.549954e+00	8.469949e+00
Au	-4.411378e+00	1.276028e+01	8.472204e+00
Au	-7.351994e+00	1.275245e+01	8.477347e+00
Au	-5.876302e+00	1.020602e+01	8.478409e+00
Au	1.033231e+01	7.649534e+00	8.484905e+00
Au	8.875574e+00	5.097669e+00	8.492225e+00
Au	8.876629e+00	1.020259e+01	8.494534e+00
Au	7.407048e+00	7.653790e+00	8.496273e+00
O	4.293955e+00	1.068590e+01	1.021992e+01
O	4.256656e+00	4.516969e+00	1.024511e+01
S	4.292119e+00	9.907175e+00	1.153002e+01
O	5.570771e+00	6.216371e+00	1.153749e+01
S	4.270227e+00	5.306393e+00	1.154648e+01
O	5.602294e+00	9.011735e+00	1.155309e+01
O	3.041476e+00	8.985111e+00	1.161479e+01
O	3.011807e+00	6.216388e+00	1.164631e+01
O	7.664165e+00	7.541990e+00	1.240832e+01
O	4.366788e+00	1.097115e+01	1.263041e+01
O	4.380687e+00	4.256912e+00	1.265778e+01
O	9.831676e-01	7.637745e+00	1.274199e+01
O	1.013426e+01	7.584445e+00	1.276731e+01
K	7.358786e+00	1.070044e+01	1.280332e+01
K	7.346327e+00	4.551342e+00	1.282144e+01
Pt	5.588738e+00	7.606862e+00	1.307375e+01
Pt	3.036940e+00	7.619368e+00	1.318002e+01
O	-1.445271e+00	7.649729e+00	1.324886e+01
S	8.827857e+00	7.583654e+00	1.345215e+01
S	-1.038144e-01	7.643001e+00	1.386265e+01
O	8.655714e+00	8.892109e+00	1.433068e+01
O	5.872221e+00	9.116385e+00	1.437493e+01
O	5.863990e+00	6.108519e+00	1.438753e+01
O	8.687503e+00	6.312506e+00	1.438775e+01
O	2.839537e+00	9.116573e+00	1.450001e+01
O	2.832719e+00	6.139843e+00	1.451753e+01
O	5.703455e+00	1.194944e+01	1.456905e+01
O	5.679637e+00	3.287656e+00	1.460523e+01
Pt	4.357713e+00	1.041384e+01	1.468850e+01
Pt	4.339202e+00	4.828507e+00	1.471208e+01
O	2.966744e+00	1.188827e+01	1.474397e+01
O	6.413634e-02	8.928652e+00	1.477674e+01
O	5.779686e-02	6.356681e+00	1.477690e+01
O	2.928101e+00	3.372412e+00	1.477976e+01
O	7.779863e+00	1.094377e+01	1.560156e+01
O	7.752490e+00	4.282561e+00	1.564769e+01

O	7.370648e+00	1.340517e+01	1.573213e+01
S	6.730252e+00	1.207840e+01	1.578476e+01
O	7.320023e+00	1.826176e+00	1.580233e+01
S	6.691613e+00	3.159119e+00	1.583311e+01
Pt	7.253730e+00	8.878081e+00	1.583693e+01
Pt	7.244734e+00	6.351819e+00	1.585453e+01
Pt	1.610642e+00	8.905786e+00	1.606547e+01
Pt	1.596368e+00	6.378998e+00	1.607502e+01
S	2.167438e+00	1.210258e+01	1.608605e+01
S	2.120556e+00	3.179686e+00	1.612163e+01
O	1.540796e+00	1.343597e+01	1.612615e+01
O	1.479161e+00	1.853155e+00	1.617028e+01
O	1.106762e+00	1.098038e+01	1.628240e+01
O	1.070881e+00	4.316033e+00	1.630184e+01
O	8.801746e+00	8.901399e+00	1.712406e+01
O	5.928602e+00	1.188572e+01	1.712875e+01
O	8.778924e+00	6.330215e+00	1.715910e+01
O	5.880373e+00	3.373566e+00	1.716811e+01
Pt	4.522398e+00	1.042650e+01	1.719939e+01
Pt	4.485682e+00	4.842188e+00	1.722277e+01
O	3.194208e+00	1.197661e+01	1.730147e+01
O	3.138132e+00	3.308461e+00	1.734475e+01
O	6.029085e+00	9.119491e+00	1.740272e+01
O	6.000786e+00	6.145575e+00	1.740835e+01
O	2.995976e+00	9.145079e+00	1.752398e+01
O	2.974378e+00	6.137345e+00	1.754374e+01
O	1.583439e-01	6.369864e+00	1.754759e+01
O	2.032953e-01	8.947365e+00	1.756674e+01
S	8.956330e+00	7.626779e+00	1.805555e+01
S	2.748090e-02	7.653947e+00	1.846666e+01
O	1.029671e+01	7.624205e+00	1.867211e+01
Pt	5.817422e+00	7.637679e+00	1.873718e+01
Pt	3.267045e+00	7.647125e+00	1.884204e+01
K	1.527083e+00	1.075874e+01	1.907331e+01
K	1.479700e+00	4.608121e+00	1.912505e+01
O	-1.275914e+00	7.668208e+00	1.915699e+01
O	7.869126e+00	7.649572e+00	1.917557e+01
O	4.512691e+00	1.100726e+01	1.925218e+01
O	4.436773e+00	4.287896e+00	1.928053e+01
O	1.193672e+00	7.622892e+00	1.950861e+01
O	5.837545e+00	6.238751e+00	2.027558e+01
O	5.821079e+00	9.020856e+00	2.029557e+01
O	3.260852e+00	9.068743e+00	2.034658e+01
S	4.577488e+00	9.955152e+00	2.036538e+01
S	4.568911e+00	5.344730e+00	2.038302e+01
O	3.279028e+00	6.269291e+00	2.039433e+01
O	4.569600e+00	1.075001e+01	2.166516e+01
O	4.587278e+00	4.555773e+00	2.168817e+01
Au	7.737670e+00	7.692594e+00	2.343701e+01
Au	9.207250e+00	1.024140e+01	2.343875e+01
Au	9.206196e+00	5.136473e+00	2.344106e+01
Au	1.066293e+01	7.688339e+00	2.344838e+01
Au	-5.545680e+00	1.024483e+01	2.345487e+01
Au	-7.021372e+00	1.279126e+01	2.345594e+01
Au	-4.080757e+00	1.279908e+01	2.346108e+01
Au	7.714700e+00	2.588758e+00	2.346333e+01
Au	7.711455e+00	1.279864e+01	2.346632e+01
Au	-4.084192e+00	7.690796e+00	2.346816e+01
Au	1.212314e+01	5.132533e+00	2.347067e+01
Au	6.238796e+00	3.410715e-02	2.347113e+01
Au	-2.624452e+00	1.024592e+01	2.347193e+01
Au	3.270575e+00	3.751704e-02	2.347584e+01

Au	1.064887e+01	2.583321e+00	2.347615e+01
Au	9.172685e+00	4.175465e-02	2.347922e+01
Au	-1.141613e+00	1.279349e+01	2.347962e+01
Au	-1.166128e+00	7.691272e+00	2.348182e+01
Au	3.306214e-01	3.880450e-02	2.348398e+01
Au	-2.628425e+00	5.133171e+00	2.348750e+01
Au	1.358777e+01	2.583228e+00	2.349196e+01
Au	2.850647e-01	1.024065e+01	2.349360e+01
Au	1.211453e+01	4.081815e-02	2.349611e+01
Au	1.506433e+01	4.083409e-02	2.349662e+01
Au	-1.146983e+00	2.584879e+00	2.350377e+01
Au	4.750599e+00	7.692450e+00	2.350677e+01
Au	1.793319e+00	1.280701e+01	2.350754e+01
Au	2.775036e-01	5.140657e+00	2.350791e+01
Au	1.789642e+00	2.584421e+00	2.351064e+01
Au	1.752673e+00	7.691834e+00	2.351151e+01
Au	4.761232e+00	2.560311e+00	2.351572e+01
Au	6.292286e+00	5.154091e+00	2.352233e+01
Au	4.760925e+00	1.281219e+01	2.353175e+01
Au	6.298561e+00	1.022875e+01	2.354234e+01
Au	3.175354e+00	5.171404e+00	2.359670e+01
Au	3.167141e+00	1.021945e+01	2.361277e+01
Au	3.306214e-01	1.739140e+00	2.588861e+01
Au	3.275689e+00	1.739140e+00	2.588861e+01
Au	6.220757e+00	1.739140e+00	2.588861e+01
Au	9.165825e+00	1.739140e+00	2.588861e+01
Au	1.211089e+01	1.739140e+00	2.588861e+01
Au	1.505596e+01	1.739140e+00	2.588861e+01
Au	-1.141913e+00	4.289644e+00	2.588861e+01
Au	1.803155e+00	4.289644e+00	2.588861e+01
Au	4.748223e+00	4.289644e+00	2.588861e+01
Au	7.693291e+00	4.289644e+00	2.588861e+01
Au	1.063836e+01	4.289644e+00	2.588861e+01
Au	1.358343e+01	4.289644e+00	2.588861e+01
Au	-2.614447e+00	6.840148e+00	2.588861e+01
Au	3.306214e-01	6.840148e+00	2.588861e+01
Au	3.275689e+00	6.840148e+00	2.588861e+01
Au	6.220757e+00	6.840148e+00	2.588861e+01
Au	9.165825e+00	6.840148e+00	2.588861e+01
Au	1.211089e+01	6.840148e+00	2.588861e+01
Au	-4.086981e+00	9.390651e+00	2.588861e+01
Au	-1.141913e+00	9.390651e+00	2.588861e+01
Au	1.803155e+00	9.390651e+00	2.588861e+01
Au	4.748223e+00	9.390651e+00	2.588861e+01
Au	7.693291e+00	9.390651e+00	2.588861e+01
Au	1.063836e+01	9.390651e+00	2.588861e+01
Au	-5.559515e+00	1.194116e+01	2.588861e+01
Au	-2.614447e+00	1.194116e+01	2.588861e+01
Au	3.306214e-01	1.194116e+01	2.588861e+01
Au	3.275689e+00	1.194116e+01	2.588861e+01
Au	6.220757e+00	1.194116e+01	2.588861e+01
Au	9.165825e+00	1.194116e+01	2.588861e+01
Au	-7.032049e+00	1.449166e+01	2.588861e+01
Au	-4.086981e+00	1.449166e+01	2.588861e+01
Au	-1.141913e+00	1.449166e+01	2.588861e+01
Au	1.803155e+00	1.449166e+01	2.588861e+01
Au	4.748223e+00	1.449166e+01	2.588861e+01
Au	7.693291e+00	1.449166e+01	2.588861e+01
Au	1.803155e+00	8.889724e-01	2.829325e+01
Au	4.748223e+00	8.889724e-01	2.829325e+01
Au	7.693291e+00	8.889724e-01	2.829325e+01
Au	1.063836e+01	8.889724e-01	2.829325e+01

Au	1.358343e+01	8.889724e-01	2.829325e+01
Au	1.652850e+01	8.889724e-01	2.829325e+01
Au	3.306214e-01	3.439476e+00	2.829325e+01
Au	3.275689e+00	3.439476e+00	2.829325e+01
Au	6.220757e+00	3.439476e+00	2.829325e+01
Au	9.165825e+00	3.439476e+00	2.829325e+01
Au	1.211089e+01	3.439476e+00	2.829325e+01
Au	1.505596e+01	3.439476e+00	2.829325e+01
Au	-1.141913e+00	5.989980e+00	2.829325e+01
Au	1.803155e+00	5.989980e+00	2.829325e+01
Au	4.748223e+00	5.989980e+00	2.829325e+01
Au	7.693291e+00	5.989980e+00	2.829325e+01
Au	1.063836e+01	5.989980e+00	2.829325e+01
Au	1.358343e+01	5.989980e+00	2.829325e+01
Au	-2.614447e+00	8.540483e+00	2.829325e+01
Au	3.306214e-01	8.540483e+00	2.829325e+01
Au	3.275689e+00	8.540483e+00	2.829325e+01
Au	6.220757e+00	8.540483e+00	2.829325e+01
Au	9.165825e+00	8.540483e+00	2.829325e+01
Au	1.211089e+01	8.540483e+00	2.829325e+01
Au	-4.086981e+00	1.109099e+01	2.829325e+01
Au	-1.141913e+00	1.109099e+01	2.829325e+01
Au	1.803155e+00	1.109099e+01	2.829325e+01
Au	4.748223e+00	1.109099e+01	2.829325e+01
Au	7.693291e+00	1.109099e+01	2.829325e+01
Au	1.063836e+01	1.109099e+01	2.829325e+01
Au	-5.559515e+00	1.364149e+01	2.829325e+01
Au	-2.614447e+00	1.364149e+01	2.829325e+01
Au	3.306214e-01	1.364149e+01	2.829325e+01
Au	3.275689e+00	1.364149e+01	2.829325e+01
Au	6.220757e+00	1.364149e+01	2.829325e+01
Au	9.165825e+00	1.364149e+01	2.829325e+01
Au	3.306214e-01	3.880450e-02	3.069789e+01
Au	3.275689e+00	3.880450e-02	3.069789e+01
Au	6.220757e+00	3.880450e-02	3.069789e+01
Au	9.165825e+00	3.880450e-02	3.069789e+01
Au	1.211089e+01	3.880450e-02	3.069789e+01
Au	1.505596e+01	3.880450e-02	3.069789e+01
Au	-1.141913e+00	2.589308e+00	3.069789e+01
Au	1.803155e+00	2.589308e+00	3.069789e+01
Au	4.748223e+00	2.589308e+00	3.069789e+01
Au	7.693291e+00	2.589308e+00	3.069789e+01
Au	1.063836e+01	2.589308e+00	3.069789e+01
Au	1.358343e+01	2.589308e+00	3.069789e+01
Au	-2.614447e+00	5.139812e+00	3.069789e+01
Au	3.306214e-01	5.139812e+00	3.069789e+01
Au	3.275689e+00	5.139812e+00	3.069789e+01
Au	6.220757e+00	5.139812e+00	3.069789e+01
Au	9.165825e+00	5.139812e+00	3.069789e+01
Au	1.211089e+01	5.139812e+00	3.069789e+01
Au	-4.086981e+00	7.690316e+00	3.069789e+01
Au	-1.141913e+00	7.690316e+00	3.069789e+01
Au	1.803155e+00	7.690316e+00	3.069789e+01
Au	4.748223e+00	7.690316e+00	3.069789e+01
Au	7.693291e+00	7.690316e+00	3.069789e+01
Au	1.063836e+01	7.690316e+00	3.069789e+01
Au	-5.559515e+00	1.024082e+01	3.069789e+01
Au	-2.614447e+00	1.024082e+01	3.069789e+01
Au	3.306214e-01	1.024082e+01	3.069789e+01
Au	3.275689e+00	1.024082e+01	3.069789e+01
Au	6.220757e+00	1.024082e+01	3.069789e+01
Au	9.165825e+00	1.024082e+01	3.069789e+01

Au -7.032049e+00 1.279132e+01 3.069789e+01
Au -4.086981e+00 1.279132e+01 3.069789e+01
Au -1.141913e+00 1.279132e+01 3.069789e+01
Au 1.803155e+00 1.279132e+01 3.069789e+01
Au 4.748223e+00 1.279132e+01 3.069789e+01
Au 7.693291e+00 1.279132e+01 3.069789e+01

Cartesian coordinates (in Angstroms) of the Au/POM/Au junction with $\{K_8[Pt_{12}O_8(SO_4)_{12}]\}$.
376
xyz

Au 0.000000e+00 0.000000e+00 1.453735e+00
Au 2.945068e+00 0.000000e+00 1.453735e+00
Au 5.890136e+00 0.000000e+00 1.453735e+00
Au 8.835204e+00 0.000000e+00 1.453735e+00
Au 1.178027e+01 0.000000e+00 1.453735e+00
Au 1.472534e+01 0.000000e+00 1.453735e+00
Au -1.472534e+00 2.550504e+00 1.453735e+00
Au 1.472534e+00 2.550504e+00 1.453735e+00
Au 4.417602e+00 2.550504e+00 1.453735e+00
Au 7.362670e+00 2.550504e+00 1.453735e+00
Au 1.030774e+01 2.550504e+00 1.453735e+00
Au 1.325281e+01 2.550504e+00 1.453735e+00
Au -2.945068e+00 5.101007e+00 1.453735e+00
Au -9.000000e-12 5.101007e+00 1.453735e+00
Au 2.945068e+00 5.101007e+00 1.453735e+00
Au 5.890136e+00 5.101007e+00 1.453735e+00
Au 8.835204e+00 5.101007e+00 1.453735e+00
Au 1.178027e+01 5.101007e+00 1.453735e+00
Au -4.417602e+00 7.651511e+00 1.453735e+00
Au -1.472534e+00 7.651511e+00 1.453735e+00
Au 1.472534e+00 7.651511e+00 1.453735e+00
Au 4.417602e+00 7.651511e+00 1.453735e+00
Au 7.362670e+00 7.651511e+00 1.453735e+00
Au 1.030774e+01 7.651511e+00 1.453735e+00
Au -5.890136e+00 1.020201e+01 1.453735e+00
Au -2.945068e+00 1.020201e+01 1.453735e+00
Au -0.000000e+00 1.020201e+01 1.453735e+00
Au 2.945068e+00 1.020201e+01 1.453735e+00
Au 5.890136e+00 1.020201e+01 1.453735e+00
Au 8.835204e+00 1.020201e+01 1.453735e+00
Au -7.362670e+00 1.275252e+01 1.453735e+00
Au -4.417602e+00 1.275252e+01 1.453735e+00
Au -1.472534e+00 1.275252e+01 1.453735e+00
Au 1.472534e+00 1.275252e+01 1.453735e+00
Au 4.417602e+00 1.275252e+01 1.453735e+00
Au 7.362670e+00 1.275252e+01 1.453735e+00
Au 1.472534e+00 8.501679e-01 3.858373e+00
Au 4.417602e+00 8.501679e-01 3.858373e+00
Au 7.362670e+00 8.501679e-01 3.858373e+00
Au 1.030774e+01 8.501679e-01 3.858373e+00
Au 1.325281e+01 8.501679e-01 3.858373e+00
Au 1.619787e+01 8.501679e-01 3.858373e+00
Au 9.000000e-12 3.400672e+00 3.858373e+00
Au 2.945068e+00 3.400672e+00 3.858373e+00
Au 5.890136e+00 3.400672e+00 3.858373e+00
Au 8.835204e+00 3.400672e+00 3.858373e+00
Au 1.178027e+01 3.400672e+00 3.858373e+00
Au 1.472534e+01 3.400672e+00 3.858373e+00
Au -1.472534e+00 5.951175e+00 3.858373e+00
Au 1.472534e+00 5.951175e+00 3.858373e+00

Au	4.417602e+00	5.951175e+00	3.858373e+00
Au	7.362670e+00	5.951175e+00	3.858373e+00
Au	1.030774e+01	5.951175e+00	3.858373e+00
Au	1.325281e+01	5.951175e+00	3.858373e+00
Au	-2.945068e+00	8.501679e+00	3.858373e+00
Au	0.000000e+00	8.501679e+00	3.858373e+00
Au	2.945068e+00	8.501679e+00	3.858373e+00
Au	5.890136e+00	8.501679e+00	3.858373e+00
Au	8.835204e+00	8.501679e+00	3.858373e+00
Au	1.178027e+01	8.501679e+00	3.858373e+00
Au	-4.417602e+00	1.105218e+01	3.858373e+00
Au	-1.472534e+00	1.105218e+01	3.858373e+00
Au	1.472534e+00	1.105218e+01	3.858373e+00
Au	4.417602e+00	1.105218e+01	3.858373e+00
Au	7.362670e+00	1.105218e+01	3.858373e+00
Au	1.030774e+01	1.105218e+01	3.858373e+00
Au	-5.890136e+00	1.360269e+01	3.858373e+00
Au	-2.945068e+00	1.360269e+01	3.858373e+00
Au	-9.000000e-12	1.360269e+01	3.858373e+00
Au	2.945068e+00	1.360269e+01	3.858373e+00
Au	5.890136e+00	1.360269e+01	3.858373e+00
Au	8.835204e+00	1.360269e+01	3.858373e+00
Au	0.000000e+00	1.700336e+00	6.263011e+00
Au	2.945068e+00	1.700336e+00	6.263011e+00
Au	5.890136e+00	1.700336e+00	6.263011e+00
Au	8.835204e+00	1.700336e+00	6.263011e+00
Au	1.178027e+01	1.700336e+00	6.263011e+00
Au	1.472534e+01	1.700336e+00	6.263011e+00
Au	-1.472534e+00	4.250839e+00	6.263011e+00
Au	1.472534e+00	4.250839e+00	6.263011e+00
Au	4.417602e+00	4.250839e+00	6.263011e+00
Au	7.362670e+00	4.250839e+00	6.263011e+00
Au	1.030774e+01	4.250839e+00	6.263011e+00
Au	1.325281e+01	4.250839e+00	6.263011e+00
Au	-2.945068e+00	6.801343e+00	6.263011e+00
Au	0.000000e+00	6.801343e+00	6.263011e+00
Au	2.945068e+00	6.801343e+00	6.263011e+00
Au	5.890136e+00	6.801343e+00	6.263011e+00
Au	8.835204e+00	6.801343e+00	6.263011e+00
Au	1.178027e+01	6.801343e+00	6.263011e+00
Au	-4.417602e+00	9.351847e+00	6.263011e+00
Au	-1.472534e+00	9.351847e+00	6.263011e+00
Au	1.472534e+00	9.351847e+00	6.263011e+00
Au	4.417602e+00	9.351847e+00	6.263011e+00
Au	7.362670e+00	9.351847e+00	6.263011e+00
Au	1.030774e+01	9.351847e+00	6.263011e+00
Au	-5.890136e+00	1.190235e+01	6.263011e+00
Au	-2.945068e+00	1.190235e+01	6.263011e+00
Au	9.000000e-12	1.190235e+01	6.263011e+00
Au	2.945068e+00	1.190235e+01	6.263011e+00
Au	5.890136e+00	1.190235e+01	6.263011e+00
Au	8.835204e+00	1.190235e+01	6.263011e+00
Au	-7.362670e+00	1.445285e+01	6.263011e+00
Au	-4.417602e+00	1.445285e+01	6.263011e+00
Au	-1.472534e+00	1.445285e+01	6.263011e+00
Au	1.472534e+00	1.445285e+01	6.263011e+00
Au	4.417602e+00	1.445285e+01	6.263011e+00
Au	7.362670e+00	1.445285e+01	6.263011e+00
Au	6.061381e+00	1.014458e+01	8.435321e+00
Au	6.046016e+00	5.152840e+00	8.474391e+00
Au	4.424274e+00	1.279969e+01	8.544150e+00
Au	4.413348e+00	2.469931e+00	8.567970e+00

Au	2.874893e+00	1.018339e+01	8.573864e+00
Au	2.874895e+00	5.110964e+00	8.595979e+00
Au	7.398119e+00	1.275307e+01	8.619450e+00
Au	7.456885e+00	7.646547e+00	8.628551e+00
Au	7.395082e+00	2.535282e+00	8.630355e+00
Au	4.428257e+00	7.650920e+00	8.636358e+00
Au	1.473760e+01	8.504808e-03	8.636920e+00
Au	1.178220e+01	8.388511e-03	8.643436e+00
Au	8.915903e+00	5.087583e+00	8.653028e+00
Au	8.845343e+00	-1.223095e-02	8.655188e+00
Au	1.326598e+01	2.544908e+00	8.655226e+00
Au	1.036048e+01	7.635831e+00	8.656320e+00
Au	1.032754e+01	2.539577e+00	8.656886e+00
Au	5.910191e+00	-3.093628e-02	8.659130e+00
Au	8.923186e+00	1.018883e+01	8.659206e+00
Au	2.930675e+00	-3.359885e-02	8.660110e+00
Au	1.181375e+01	5.085356e+00	8.664448e+00
Au	0.000000e+00	0.000000e+00	8.667649e+00
Au	-5.857548e+00	1.019327e+01	8.669458e+00
Au	-7.347709e+00	1.273674e+01	8.672075e+00
Au	1.464042e+00	1.274784e+01	8.673470e+00
Au	-4.396835e+00	7.643348e+00	8.678622e+00
Au	-4.406358e+00	1.274234e+01	8.681769e+00
Au	-2.938271e+00	5.091074e+00	8.684454e+00
Au	-1.472768e+00	2.545343e+00	8.685515e+00
Au	-1.464838e+00	1.273812e+01	8.695538e+00
Au	1.455116e+00	2.543837e+00	8.696697e+00
Au	-2.938494e+00	1.019827e+01	8.697064e+00
Au	1.436330e+00	7.645044e+00	8.712599e+00
Au	-2.930885e-02	5.088477e+00	8.724946e+00
Au	-1.479428e+00	7.641917e+00	8.729052e+00
Au	-2.334753e-02	1.019493e+01	8.730375e+00
O	4.703162e+00	1.082538e+01	1.027931e+01
O	4.671065e+00	4.359900e+00	1.029404e+01
O	3.420700e+00	8.923548e+00	1.138888e+01
S	4.612175e+00	9.949000e+00	1.152393e+01
S	4.645580e+00	5.217137e+00	1.155099e+01
O	3.329286e+00	6.080798e+00	1.155130e+01
O	5.889231e+00	6.173828e+00	1.160887e+01
O	5.944791e+00	9.153586e+00	1.173576e+01
O	1.211693e+00	7.634593e+00	1.233841e+01
K	1.496577e+00	1.050022e+01	1.252792e+01
O	4.324688e+00	1.095896e+01	1.264367e+01
O	-1.268744e+00	7.654037e+00	1.265449e+01
O	4.676915e+00	4.186988e+00	1.268886e+01
O	7.979795e+00	7.515841e+00	1.271664e+01
K	1.417983e+00	4.570735e+00	1.273457e+01
K	7.653480e+00	4.633919e+00	1.295686e+01
Pt	3.285910e+00	7.579009e+00	1.303272e+01
K	7.763645e+00	1.058638e+01	1.308655e+01
Pt	5.842234e+00	7.595442e+00	1.318071e+01
O	1.042385e+01	7.493697e+00	1.326768e+01
S	2.561537e-02	7.638300e+00	1.336558e+01
S	9.065684e+00	7.512641e+00	1.384396e+01
O	1.568718e-01	6.345288e+00	1.427100e+01
O	1.764304e-01	8.919783e+00	1.428682e+01
O	2.960284e+00	9.080696e+00	1.434960e+01
O	2.974409e+00	6.095186e+00	1.436939e+01
O	6.019490e+00	9.082814e+00	1.452199e+01
O	6.022691e+00	6.096389e+00	1.453501e+01
O	3.078901e+00	3.301075e+00	1.459331e+01
O	2.978773e+00	1.181945e+01	1.460041e+01

O	5.875103e+00	1.186867e+01	1.468364e+01
Pt	4.458748e+00	1.037725e+01	1.468845e+01
Pt	4.493994e+00	4.797928e+00	1.471726e+01
O	8.852701e+00	8.810527e+00	1.472829e+01
O	8.828945e+00	6.236951e+00	1.475388e+01
O	5.969607e+00	3.351660e+00	1.477011e+01
O	9.737375e-01	4.259978e+00	1.563738e+01
O	1.435081e+00	1.799938e+00	1.576288e+01
S	2.050156e+00	3.140520e+00	1.578974e+01
Pt	1.565837e+00	6.334906e+00	1.579131e+01
Pt	1.581929e+00	8.865458e+00	1.580639e+01
O	1.505745e+00	1.341124e+01	1.589727e+01
S	2.105070e+00	1.206334e+01	1.590050e+01
O	7.888173e+00	1.091891e+01	1.591285e+01
O	7.421786e+00	1.338089e+01	1.595440e+01
O	1.008907e+00	1.095644e+01	1.595479e+01
S	6.805207e+00	1.204029e+01	1.596182e+01
Pt	7.291644e+00	8.835405e+00	1.607783e+01
Pt	7.276858e+00	6.303520e+00	1.611505e+01
S	6.739147e+00	3.110972e+00	1.613629e+01
O	7.335267e+00	1.762171e+00	1.617211e+01
O	7.834333e+00	4.215694e+00	1.627116e+01
O	1.824431e-02	8.865598e+00	1.715412e+01
O	2.817542e+00	3.383306e+00	1.715767e+01
O	2.652717e-02	6.292625e+00	1.717017e+01
O	3.032936e+00	1.188978e+01	1.717965e+01
Pt	4.434448e+00	1.038644e+01	1.718856e+01
Pt	4.319588e+00	4.804294e+00	1.720805e+01
O	5.931785e+00	1.181548e+01	1.726496e+01
O	5.719550e+00	3.296083e+00	1.733766e+01
O	2.863413e+00	9.102221e+00	1.735639e+01
O	2.810661e+00	6.122190e+00	1.738087e+01
O	5.863298e+00	6.084866e+00	1.753390e+01
O	5.920223e+00	9.079373e+00	1.753843e+01
O	8.698480e+00	8.883415e+00	1.759082e+01
O	8.697585e+00	6.309076e+00	1.762893e+01
S	-1.985426e-01	7.584037e+00	1.805981e+01
S	8.840785e+00	7.607276e+00	1.852328e+01
O	-1.553139e+00	7.586097e+00	1.864621e+01
Pt	3.024686e+00	7.629033e+00	1.871949e+01
K	1.135449e+00	1.062792e+01	1.877932e+01
Pt	5.580402e+00	7.582832e+00	1.885587e+01
K	1.190437e+00	4.706883e+00	1.901159e+01
K	7.442341e+00	4.545479e+00	1.915312e+01
O	8.896091e-01	7.597147e+00	1.918655e+01
O	1.013762e+01	7.625806e+00	1.923003e+01
O	4.579492e+00	1.098199e+01	1.923284e+01
O	4.058184e+00	4.215687e+00	1.924195e+01
K	7.402888e+00	1.049059e+01	1.934266e+01
O	7.656685e+00	7.623086e+00	1.955120e+01
O	2.940261e+00	9.194390e+00	2.014607e+01
O	5.534129e+00	6.063890e+00	2.030584e+01
O	2.981742e+00	6.254691e+00	2.034535e+01
S	4.281516e+00	9.978917e+00	2.035561e+01
S	4.187557e+00	5.250672e+00	2.036866e+01
O	5.463107e+00	8.942738e+00	2.049071e+01
O	4.173146e+00	1.085382e+01	2.159897e+01
O	4.204418e+00	4.407723e+00	2.163925e+01
Au	-4.899944e-01	1.024276e+01	2.320291e+01
Au	-1.946074e+00	7.689739e+00	2.320423e+01
Au	-4.959557e-01	5.136300e+00	2.320834e+01
Au	9.696828e-01	7.692867e+00	2.322068e+01

Au	-3.405141e+00	1.024609e+01	2.323622e+01
Au	9.884689e-01	2.591659e+00	2.323659e+01
Au	-1.931485e+00	1.278595e+01	2.323775e+01
Au	-1.939414e+00	2.593166e+00	2.324777e+01
Au	-3.404918e+00	5.138896e+00	2.324883e+01
Au	-4.873005e+00	1.279017e+01	2.325151e+01
Au	-4.863481e+00	7.691170e+00	2.325466e+01
Au	9.973950e-01	1.279566e+01	2.325981e+01
Au	-7.814356e+00	1.278456e+01	2.326121e+01
Au	-6.324194e+00	1.024110e+01	2.326383e+01
Au	-4.666468e-01	4.782247e-02	2.326564e+01
Au	1.134710e+01	5.133178e+00	2.326884e+01
Au	2.464028e+00	1.422362e-02	2.327317e+01
Au	8.456539e+00	1.023666e+01	2.327408e+01
Au	5.443544e+00	1.688619e-02	2.327415e+01
Au	9.860893e+00	2.587399e+00	2.327640e+01
Au	9.893831e+00	7.683653e+00	2.327696e+01
Au	1.279933e+01	2.592731e+00	2.327806e+01
Au	8.378696e+00	3.559152e-02	2.327810e+01
Au	8.449256e+00	5.135405e+00	2.328026e+01
Au	1.131555e+01	5.621098e-02	2.328985e+01
Au	1.427096e+01	5.632728e-02	2.329636e+01
Au	3.961610e+00	7.698743e+00	2.329693e+01
Au	6.928435e+00	2.583104e+00	2.330293e+01
Au	6.990238e+00	7.694369e+00	2.330473e+01
Au	6.931472e+00	1.280090e+01	2.331383e+01
Au	2.408248e+00	5.158786e+00	2.333731e+01
Au	2.408246e+00	1.023121e+01	2.335942e+01
Au	3.946701e+00	2.517754e+00	2.336531e+01
Au	3.957627e+00	1.284752e+01	2.338913e+01
Au	5.579369e+00	5.200662e+00	2.345889e+01
Au	5.594734e+00	1.019240e+01	2.349796e+01
Au	-4.666468e-01	1.748158e+00	2.567027e+01
Au	2.478421e+00	1.748158e+00	2.567027e+01
Au	5.423489e+00	1.748158e+00	2.567027e+01
Au	8.368557e+00	1.748158e+00	2.567027e+01
Au	1.131363e+01	1.748158e+00	2.567027e+01
Au	1.425869e+01	1.748158e+00	2.567027e+01
Au	-1.939181e+00	4.298662e+00	2.567027e+01
Au	1.005887e+00	4.298662e+00	2.567027e+01
Au	3.950955e+00	4.298662e+00	2.567027e+01
Au	6.896023e+00	4.298662e+00	2.567027e+01
Au	9.841091e+00	4.298662e+00	2.567027e+01
Au	1.278616e+01	4.298662e+00	2.567027e+01
Au	-3.411715e+00	6.849166e+00	2.567027e+01
Au	-4.666468e-01	6.849166e+00	2.567027e+01
Au	2.478421e+00	6.849166e+00	2.567027e+01
Au	5.423489e+00	6.849166e+00	2.567027e+01
Au	8.368557e+00	6.849166e+00	2.567027e+01
Au	1.131363e+01	6.849166e+00	2.567027e+01
Au	-4.884249e+00	9.399669e+00	2.567027e+01
Au	-1.939181e+00	9.399669e+00	2.567027e+01
Au	1.005887e+00	9.399669e+00	2.567027e+01
Au	3.950955e+00	9.399669e+00	2.567027e+01
Au	6.896023e+00	9.399669e+00	2.567027e+01
Au	9.841091e+00	9.399669e+00	2.567027e+01
Au	-6.356783e+00	1.195017e+01	2.567027e+01
Au	-3.411715e+00	1.195017e+01	2.567027e+01
Au	-4.666468e-01	1.195017e+01	2.567027e+01
Au	2.478421e+00	1.195017e+01	2.567027e+01
Au	5.423489e+00	1.195017e+01	2.567027e+01
Au	8.368557e+00	1.195017e+01	2.567027e+01

Au	-7.829317e+00	1.450068e+01	2.567027e+01
Au	-4.884249e+00	1.450068e+01	2.567027e+01
Au	-1.939181e+00	1.450068e+01	2.567027e+01
Au	1.005887e+00	1.450068e+01	2.567027e+01
Au	3.950955e+00	1.450068e+01	2.567027e+01
Au	6.896023e+00	1.450068e+01	2.567027e+01
Au	1.005887e+00	8.979904e-01	2.807491e+01
Au	3.950955e+00	8.979904e-01	2.807491e+01
Au	6.896023e+00	8.979904e-01	2.807491e+01
Au	9.841091e+00	8.979904e-01	2.807491e+01
Au	1.278616e+01	8.979904e-01	2.807491e+01
Au	1.573123e+01	8.979904e-01	2.807491e+01
Au	-4.666468e-01	3.448494e+00	2.807491e+01
Au	2.478421e+00	3.448494e+00	2.807491e+01
Au	5.423489e+00	3.448494e+00	2.807491e+01
Au	8.368557e+00	3.448494e+00	2.807491e+01
Au	1.131363e+01	3.448494e+00	2.807491e+01
Au	1.425869e+01	3.448494e+00	2.807491e+01
Au	-1.939181e+00	5.998998e+00	2.807491e+01
Au	1.005887e+00	5.998998e+00	2.807491e+01
Au	3.950955e+00	5.998998e+00	2.807491e+01
Au	6.896023e+00	5.998998e+00	2.807491e+01
Au	9.841091e+00	5.998998e+00	2.807491e+01
Au	1.278616e+01	5.998998e+00	2.807491e+01
Au	-3.411715e+00	8.549501e+00	2.807491e+01
Au	-4.666468e-01	8.549501e+00	2.807491e+01
Au	2.478421e+00	8.549501e+00	2.807491e+01
Au	5.423489e+00	8.549501e+00	2.807491e+01
Au	8.368557e+00	8.549501e+00	2.807491e+01
Au	1.131363e+01	8.549501e+00	2.807491e+01
Au	-4.884249e+00	1.110001e+01	2.807491e+01
Au	-1.939181e+00	1.110001e+01	2.807491e+01
Au	1.005887e+00	1.110001e+01	2.807491e+01
Au	3.950955e+00	1.110001e+01	2.807491e+01
Au	6.896023e+00	1.110001e+01	2.807491e+01
Au	9.841091e+00	1.110001e+01	2.807491e+01
Au	-6.356783e+00	1.365051e+01	2.807491e+01
Au	-3.411715e+00	1.365051e+01	2.807491e+01
Au	-4.666468e-01	1.365051e+01	2.807491e+01
Au	2.478421e+00	1.365051e+01	2.807491e+01
Au	5.423489e+00	1.365051e+01	2.807491e+01
Au	8.368557e+00	1.365051e+01	2.807491e+01
Au	-4.666468e-01	4.782247e-02	3.047955e+01
Au	2.478421e+00	4.782247e-02	3.047955e+01
Au	5.423489e+00	4.782247e-02	3.047955e+01
Au	8.368557e+00	4.782247e-02	3.047955e+01
Au	1.131363e+01	4.782247e-02	3.047955e+01
Au	1.425869e+01	4.782247e-02	3.047955e+01
Au	-1.939181e+00	2.598326e+00	3.047955e+01
Au	1.005887e+00	2.598326e+00	3.047955e+01
Au	3.950955e+00	2.598326e+00	3.047955e+01
Au	6.896023e+00	2.598326e+00	3.047955e+01
Au	9.841091e+00	2.598326e+00	3.047955e+01
Au	1.278616e+01	2.598326e+00	3.047955e+01
Au	-3.411715e+00	5.148830e+00	3.047955e+01
Au	-4.666468e-01	5.148830e+00	3.047955e+01
Au	2.478421e+00	5.148830e+00	3.047955e+01
Au	5.423489e+00	5.148830e+00	3.047955e+01
Au	8.368557e+00	5.148830e+00	3.047955e+01
Au	1.131363e+01	5.148830e+00	3.047955e+01
Au	-4.884249e+00	7.699334e+00	3.047955e+01
Au	-1.939181e+00	7.699334e+00	3.047955e+01

Au	1.005887e+00	7.699334e+00	3.047955e+01
Au	3.950955e+00	7.699334e+00	3.047955e+01
Au	6.896023e+00	7.699334e+00	3.047955e+01
Au	9.841091e+00	7.699334e+00	3.047955e+01
Au	-6.356783e+00	1.024984e+01	3.047955e+01
Au	-3.411715e+00	1.024984e+01	3.047955e+01
Au	-4.666468e-01	1.024984e+01	3.047955e+01
Au	2.478421e+00	1.024984e+01	3.047955e+01
Au	5.423489e+00	1.024984e+01	3.047955e+01
Au	8.368557e+00	1.024984e+01	3.047955e+01
Au	-7.829317e+00	1.280034e+01	3.047955e+01
Au	-4.884249e+00	1.280034e+01	3.047955e+01
Au	-1.939181e+00	1.280034e+01	3.047955e+01
Au	1.005887e+00	1.280034e+01	3.047955e+01
Au	3.950955e+00	1.280034e+01	3.047955e+01
Au	6.896023e+00	1.280034e+01	3.047955e+01

References:

1 C. Simms, A. Kondinski and T. N. Parac-Vogt, *Eur. J. Inorg. Chem.*, 2020, 2559–2572.

2 A. Kondinski, *Chem. Model.*, 2021, **16**, 39–71.

3 A. Kondinski, *Nanoscale*, 2021, DOI:10.1039/D1NR02357H.

4 V. Stavila, A. A. Talin and M. D. Allendorf, *Chem. Soc. Rev.*, 2014, **43**, 5994–6010.

5 M. Wang, R. Dong and X. Feng, *Chem. Soc. Rev.*, 2021, **50**, 2764–2793.

6 J. Waser and E. D. McClanahan, *J. Chem. Phys.*, 1951, **19**, 413–416.

7 M. Pley and M. S. Wickleder, *Angew. Chemie Int. Ed.*, 2004, **43**, 4168–4170.
